# Evidence that autosomal recessive spastic cerebral palsy-1 (CPSQ1) is caused by a missense variant in *HPDL*

**DOI:** 10.1093/braincomms/fcab002

**Published:** 2021-01-28

**Authors:** Neil V Morgan, Bryndis Yngvadottir, Mary O’Driscoll, Graeme R Clark, Diana Walsh, Ezequiel Martin, Louise Tee, Evan Reid, Hannah L Titheradge, Eamonn R Maher

**Affiliations:** 1 Institute of Cardiovascular Sciences, College of Medical and Dental Sciences, University of Birmingham, Birmingham B15 2TT, UK; 2 Department of Medical Genetics, University of Cambridge and NIHR Cambridge Biomedical Research Centre, Cambridge CB2 0QQ, UK; 3 West Midlands Regional Genetics Service and Birmingham Health Partners, Birmingham Women’s and Children’s NHS Trust, Birmingham B15 2TG, UK; 4 West Midlands Regional Genetics Laboratory, Birmingham Women’s and Children’s NHS Trust, Birmingham B15 2TG, UK; 5 Oncology Department, Cancer Molecular Diagnostics Laboratory, University of Cambridge, Cambridge CB2 0XZ, UK; 6 Institute of Cancer and Genomic Sciences, College of Medical and Dental Sciences, University of Birmingham, Birmingham B15 2TT, UK; 7 Cambridge Institute of Medical Research, University of Cambridge, Cambridge CB2 0XY, UK

**Keywords:** autosomal recessive, cerebral palsy, inherited

## Abstract

A subset of individuals diagnosed with cerebral palsy will have an underlying genetic diagnosis. Previously, a missense variant in *GAD1* was described as a candidate mutation in a single family diagnosed with autosomal recessive spastic cerebral palsy-1 (CPSQ1; OMIM 603513). Following the ascertainment of a further branch of the CPSQ1 kindred, we found that the previously reported *GAD1* variant did not segregate with the neurological disease phenotype in the recently ascertained branch of the kindred. Following genetic linkage studies to map autozygous regions and whole-exome sequencing, a missense variant (c.527 T > C; p. Leu176Pro, rs773333490) in the *HPDL* gene was detected and found to segregate with disease status in both branches of the kindred. *HPDL* encodes a 371-amino acid protein (4-Hydroxyphenylpyruvate Dioxygenase Like) that localizes to mitochondria but whose function is uncertain. Recently, biallelic loss of function variants and missense substitution-causing variants in *HPDL* were reported to cause a childhood onset progressive spastic movement disorder with a variable presentation. These findings suggest that *HPDL-*related neurological disease may mimic spastic cerebral palsy and that *GAD1* should not be included in diagnostic gene panels for inherited cerebral palsy.

## Teaser

Morgan *et al.* report that a novel candidate mutation in the *HPDL* gene (but not *GAD1*) segregates with disease status within a kindred in which a locus for autosomal recessive spastic cerebral palsy-1 (CPSQ1; OMIM 603513, *GAD1*) was mapped previously (in part of the kindred).

## Graphical Abstract

**Graphical Abstract:**
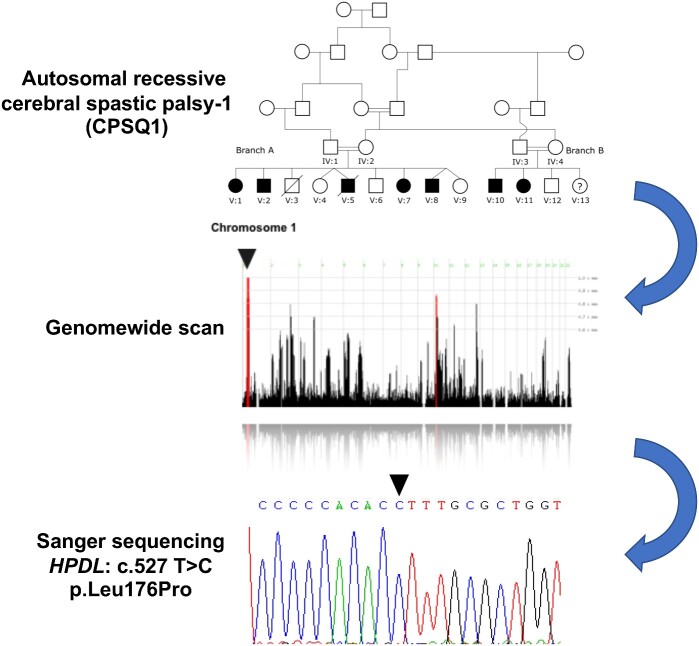

## Introduction

Cerebral palsy is a common cause of childhood neurodisability that is characterized clinically by permanent abnormalities of motor activity and posture. It typically includes spasticity and often intellectual disability and epilepsy, and results from non-progressive disturbances to the fetal or infantile brain (MacLennan *et al.*, 2019; Pearson *et al.*, 2019). In most cases, initial symptoms occur in infancy and the diagnosis of cerebral palsy is made at the age of 2 years (Pearson *et al.*, 2019). Cerebral palsy is an umbrella term, covering a heterogeneous group of conditions (MacLennan *et al.*, 2019). Traditionally, cerebral palsy was often attributed to acute hypoxia at birth but this is now believed to account for only a minority of cases. Instead, pre-natal insults are a more prominent cause, although in 25–50% of cases no cause is identified (Michael-Asalu *et al.*, 2019). However, there has been an increasing realization that inherited disorders can present as cerebral palsy. Both copy-number abnormalities and pathogenic variants in single genes have been described as causes of a cerebral palsy phenotype and inherited causes of apparent cerebral palsy may be transmitted in an autosomal dominant, autosomal recessive, X-linked or mitochondrial inheritance fashion (McMichael *et al.*, 2015; Oskoui *et al.*, 2015; Takezawa *et al.*, 2018; Michael-Asalu *et al.*, 2019). Autosomal recessively inherited disorders are a common cause of familial cerebral palsy and consanguinity has been identified as a risk factor for cerebral palsy (Erkin *et al.*, 2008; Moreno-De-Luca *et al*., 2012).

Autosomal recessively inherited disorders are an important cause of mortality and morbidity, particularly in communities with parental consanguinity and/or common founder mutations. In the United Kingdom, the overall frequency of consanguinity is <1% but it is much higher in some communities (Bundey *et al.*, 1991; Sheridan *et al.*, 2013). In one UK centre, consanguinity was associated with an increased risk of perinatal mortality from lethal malformations attributed to autosomal recessive disorders and in another centre parental-relatedness was associated with a ∼2.2-fold increased risk for congenital anomaly (Bundey *et al.*, 1991; Sheridan *et al.*, 2013). For families in which the pathogenic alleles causing a recessive disorder are characterized, carrier parents can be provided with counselling regarding their reproductive options and their at-risk relatives can be tested to determine their carrier risk. Strategies for pre-conception genetic testing have been developed to offer prospective parents the opportunity to determine their carrier status for autosomal recessive disease mutations (Bell *et al.*, 2011, Capalbo *et al.*, 2019).

The availability of genetic testing for recessive disorders is based on the reliable identification of the relevant disease gene. Lander and Botstein (1987) proposed that mapping regions of homozygosity by descent (autozygosity) in the affected children of consanguineous marriages could provide a highly efficient strategy for mapping recessive traits. With improvements in genetic maps and identification of polymorphic DNA markers, the strategy of homozygosity/autozygosity mapping was widely adopted to localize recessive genes even in the presence of locus heterogeneity (Mueller and Bishop, 1993; Sheffield *et al.*, 1995). Though for a child whose parents are first cousins, ∼6% (1/16) of the genome should be homozygous/autozygous, in communities in which consanguinity has been practiced over multiple generations, more extensive autozygosity often occurs. In consanguineous families with an autosomal recessive disorder, Woods *et al.* (2006) reported that children with first cousin parents had an average of 11% homozygosity with 20 homozygous segments (>3 cm). Additionally, the chance of the longest segment of homozygosity harbouring the disease gene was estimated at ∼1 in 6 (Woods *et al.*, 2006). In older autozygosity mapping studies, candidate genes in an autozygous region would be sequenced serially until a candidate pathogenic variant was identified (Hartley *et al.*, 2010). However, the advent of exome sequencing (Bolze *et al.*, 2010; Dang *et al.*, 2016) enabled simultaneous sequencing of all genes in the linked region and interrogation of genes in smaller autozygous segments. This is helpful because many autosomal recessive disease families with parental consanguinity that are recruited to research studies will be from non-white ethnicities and therefore will be less represented in global databases of genetic variation, making the interpretation of the pathogenicity of rare genetic variants in such cases challenging (Manolio, 2019).

More than 20 years ago, Mitchell and Bundey (1997) described seven families with parental consanguinity in which children presented with a spastic cerebral palsy-like syndrome. There was clinical heterogeneity between the families (e.g. variable degrees of learning disability and microcephaly in some cases), suggesting different underlying diagnoses but the presence of symmetrical lower limb spasticity was noted in all cases. The authors concluded that the symmetry was consistent with an underlying, probably autosomal recessively inherited, genetic origin (Mitchell and Bundey, 1997). Subsequently, genetic linkage studies were reported in a some of these families using 290 polymorphic DNA markers and a common region of autozygosity was identified at chromosome 2q24-q25 (interval, ∼5 cm) in three families, suggesting the presence of a locus for autosomal recessive symmetrical spastic cerebral palsy (McHale *et al.*, 1999). One of the families studied (Family 4 in Mitchell and Bundey (1997) and McHale *et al.*, 1999) contained four affected children and further investigations identified a rare homozygous missense substitution (c. 36 G > C; p. Ser12Cys) in the *GAD1* gene (Lynex *et al.*, 2004). This suggested that autosomal recessive spastic cerebral palsy-1 (CPSQ1; OMIM 603513) resulted from mutation in the glutamate decarboxylate-1 gene (*GAD1;* OMIM 605363). Here, we provide further genetic evidence to demonstrate that CPSQ1 is not caused by *GAD1* but by a missense substitution in a recently described gene, *HPDL*.

## Methods

### Patients

**Figure 1:**
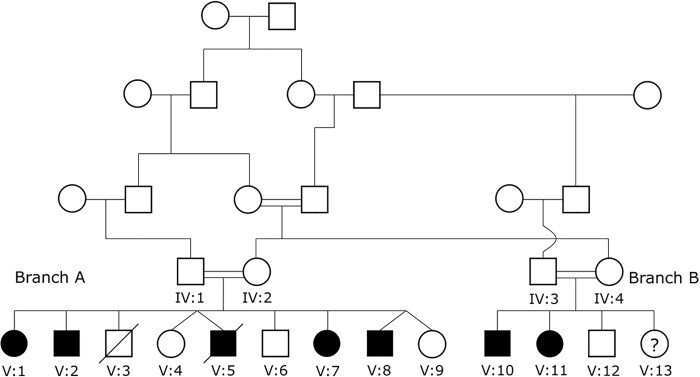
**Family structure.** A schematic representation of how two branches of the kindred were found to be connected (Branch A previously reported and Branch B subsequently ascertained, see text for details).

Molecular genetic studies were undertaken in 15 individuals from a large consanguineous British Pakistani kindred that was initially ascertained as two distinct families but later established to be part of a larger kindred. One of the families (Branch A) was originally described in the clinical report by Mitchell and Bundey (1997) (referred to as Family 4) and in molecular genetic studies reported by McHale *et al.*, (1999) (Family 4) and Lynex *et al.*, (2004) (Family B). Subsequently, a family with a similar phenotype (herein referred to as Branch B) was identified independently and then shown to have shared ancestors with Branch A (Fig. 1). All individuals gave written informed consent. The study was approved by the South Birmingham Research Ethics Committee and performed in accordance with the ethical standards laid down in the 1964 Declaration of Helsinki. Genomic DNA was extracted from the buffy coat of all individuals where possible.

### Molecular genetic analysis

#### Linkage analysis

Linkage to the chromosome 2 and *GAD1* candidate region reported in Lynex *et al.* (2004) was further investigated here in 15 individuals (Supplementary Fig. 1) from branches A and B of the kindred using microsatellite markers in the previously identified candidate region at chromosome 2q24-q31 and Sanger sequencing of the *GAD1* missense substitution was performed. Subsequently, a genome-wide linkage scan was carried out using Affymetrix Genome-Wide Human SNP 5.0 microarrays in a total of six affected individuals [five affected individuals from Branch A (V: 1, V: 2, V: 5, V: 7, V: 8) and one affected individual from Branch B (V: 10)]. Homozygosity mapping analysis was then performed and homozygous regions were identified using HomozygosityMapper (http://homozygositymapper.org) (Seelow *et al.*, 2009).

#### Exome sequencing

Exome sequencing was performed in two stages. Initially, Patient V: 5 was subjected to whole-exome sequencing using the SureSelect human All Exon 50 Mb kit (Agilent Technologies) and sequencing on the HiSeq 2500 (Illumina) with 100 bp paired-end reads. The sequences were aligned to the reference genome (GRCh37), with Novoalign (Novocraft Technologies Sdn Bhd). Duplicate reads, resulting from PCR clonality or optical duplicates, and reads mapping to multiple locations were excluded from downstream analysis. Depth and breadth of sequence coverage were calculated with custom scripts and the BedTools package. Single-nucleotide variants and small insertions and deletions (InDels) were identified and quality filtered within the SamTools software package and in-house software tools. All calls with a read coverage of <4 and a phred-scaled SNP quality of <20 were filtered out. Variants were annotated with respect to genes and transcripts with the Annovar tool (Wang *et al*., 2010). Subsequently, exome sequencing was performed in five individuals (IV: 3; IV: 4; V: 10; V: 11; V: 12) using Illumina Nextera assays. The samples were sequenced on Illumina’s HiSeq 4000 platform with 150 bp paired end reads. Raw Illumina BCL files were de-multiplexed and converted to fastq format using Illumina’s bcl2fastq 2.19, which also trimmed from the reads the indexes and the adaptors used for sequencing. All sample pairs were aligned to the hg38 version of the reference human genome using bwa 0.7.15 in alt contig aware mode as described by the authors (Li and Durbin, 2009). Further details of the bioinformatic pipeline are detailed in Supplementary Methods.

#### Sanger sequencing

Amplification of genomic DNA for Sanger sequencing for three individuals (IV: 2; V: 1; V: 5) was performed by standard PCR methods. PCR clean-up was performed with MicroClean^TM^. Sanger sequencing was then performed using the same primers, primer sequences are available on request. Sanger sequencing was performed using BigDye Terminator Cycle Sequencing Kit, version 3.1 (Applied Biosystems) and analysed on an ABI 3130XL DNA analyser (Applied Biosystems).

### Data availability

The authors confirm that all the data supporting the findings of this study are available within the article and readily available upon request.

## Results

### Clinical features

The kindred pedigree is shown in Fig. 1. The sibship-labelled Branch A was described previously (Mitchell and Bundey, 1997; McHale *et al.*, 1999; Lynex *et al.*, 2004). Subsequently, the sibship-labelled Branch B was ascertained independently and found to have a common ancestor with Branch A (Fig. 1). The clinical details of the affected individuals from both branches are summarized in Table 1. The details for four affected individuals (V: 1; V: 2; V: 5; V: 7) in Branch A were described previously (Mitchell and Bundey, 1997; McHale et al., 1999; Lynex *et al.*, 2004) but since the last report (Lynex *et al.*, 2004) a further younger male sibling (V: 8) had been diagnosed with spastic cerebral palsy. Branch A included two sets of dizygotic twins with one affected twin (both male) in each case. Branch B comprised four siblings. At the time of ascertainment, two of the siblings were clinically affected (V: 10; V: 11), one sibling was asymptomatic and judged to be unaffected (V: 12) and the clinical status of the youngest sibling had not been established (V: 13). After clinical assessment of V: 10 and V: 11 and review of the clinical descriptions of affected individuals in Family A, the working diagnosis was that both branches of the family were affected by the same neurological disorder.

**Table 1:** Summary of clinical features of affected individuals with a large consanguineous kindred comprising Branch A and Branch B

Pedigree	**Branch A (Family 4 in Mitchell and Bundey** 1997 **)**					Branch B	
Individual	V: 1	V: 2	V: 5	V: 7	V: 8	V: 10	V: 11
Sex	F	M	M	F	M	M	F
Age at last assessment (years)	14	12	9	22	12	18	19
Age signs first noticed (years)	1^a^	0.5^a^	1^a^	1^a^	2	Mid-childhood with increased falls	With delayed walking
Severe learning disability (LD)		+^a^	+^a^		–	–	–
Mild/mod LD	+^a^			+^a^	–	Mild	+
Epilepsy	No	No	No	No	No	No	No
Microcephaly	–		–	–	–	N/A	OFC 53.4 cm at 19 years
Predominantly lower limbs	+^a^	+^a^	+^a^	+^a^		+	+
Upper limb ataxia	+	+^b^	+^b^			+	+ (Mild)
Ataxic gait					Severe	+	+
Reflexes	Increased LL reflexes	Increased LL reflexes and tone	Increased LL reflexes and tone	Increased LL reflexes and tone	Increased LL reflexes and tone	Increased LL reflexes and tone	? Increased LL reflexes and tone
Plantar response	Upgoing	Equivocal	Upgoing	Upgoing	Upgoing	Upgoing	Downgoing
Other features	Bilat dislocated hips^a^ (CDH); fixed flexion deformities both knees	Thoracolumbar scoliosis^a^; fixed flexion deformities both knees and extension at hips; distal muscle wasting in LL		Age 22: dysarthria, fixed flexion at the knees; upper and lower limb wasting	muscle wasting in LL	Pes planus; poor muscle bulk; hypotonia changing to hypertonia later Weight gain	Short 4th and 5th metacarpals (unilateral); mild hyper-telorism, relative small stature to family. Flexed hips
Metabolic investigations				Plasma and urine amino acids normal^a^; Plasma lactate normal^a^	Normal plasma Very long-chain fatty acids, plasma hexosaminidase, chitotriosidase, acyl-carcitine, amino acids and lactate, Normal urinary organic acids, glycosaminoglycans and oligosaccharides	N/A	Normal plasma transferrin, lactate and plasma amino acids. Normal cholestanol
Prenatal	FTND	FTND		FTND	FTND	Prenatal growth retardation; LSCS for fetal distress; Birth weight, 5.5 lb	Normal delivery Birth weight 6.5 lb
Motor development	Never walked independently but mobilities with frame at 2.5 years; wheelchair bound after surgery for CDH at age 4 years	Crawled at 2.5 years	Crawled at 2.0 years;		Walked at 2.5 years	Facial hypotonia; Walked at 1.5 years	Late sitting; First steps at 2.5 years; Facial hypotonia
Progression	Became wheelchair dependent	Became wheelchair dependent		Became wheelchair dependent	Partially wheelchair bound age, 22 years	Reported well until 11.5 years when started falling and weight loss. Botox age 13 years associated with decreased mobility and at 22 years unable to stand and needs help to transfer	? Delayed walking at 2.5–3 years, max mobility age 10 years with deterioration from 13 years. Mainstream school with significant support
Eyes	Convergent squint^a^;			Congenital convergent squint	Horizontal ophthalmoplegia; ERG: substantially reduced responses on right, absent on left	No concerns	Esotropia. Nystagmus—jerk in horizontal plane and gaze-evoked upbeat in vertical plane; dysarthria, normal jaw jerk and upper limb co-ordination. Normal optic discs
Imaging					Normal brain and spine MRI scan (age, 9 years)	MRI scan of brain and spine reported normal (age, 8 years); MRI brain age 13 years reported to show demyelination in medulla	MRI scan brain and spine age 20 years showed no abnormality (though sub-optimal images because of movement)
Investigations					Normal nerve conduction studies, EMG and creatinine kinase normal	Normal CPK and lactate, amino acids (blood and urine), organic acids, ferritin and caeruloplasmin	Normal nerve conduction studies, EMG and creatinine kinase normal Normal microarray, Friedreich ataxia and HSP panel testing incl. SPAST/ATL1/REEP1/SPG7 MLPA

CDH, congenital dislocation of the hips; LL, lower limbs; FTND, full-term normal delivery; OFC, occipital frontal circumference.

a As reported in Mitchell and Bundey (1997).

### Molecular genetic studies

Previous linkage studies and sequencing analysis in Branch A (McHale *et al.*, 1999; Lynex *et al.*, 2004) had identified a common region of homozygosity at chromosome 2q24-q25 in four affected individuals and a rare homozygous missense substitution within *GAD1* as a candidate mutation (c. 36 G > C; p. Ser12Cys). Following the ascertainment of Branch B, we performed extended segregation analysis using microsatellite markers mapping to 2q24-q31 and genotyped the c.36G>C variant across both branches. This confirmed the finding that c. 36 G > C variant segregated with disease status in Branch A (including the newly diagnosed sibling V: 8) but that in Branch B two affected individuals (V: 10 and V: 11) were either heterozygous (V: 10) or homozygous wild type (V: 11) and the father (IV: 3) was homozygous wild type (Supplementary Fig. 1). Unless the autosomal recessive neurological disorder diagnosed in Branch A and Branch B were different, this excluded *GAD1* as the cause of the disease in this kindred.

**Figure 2:**
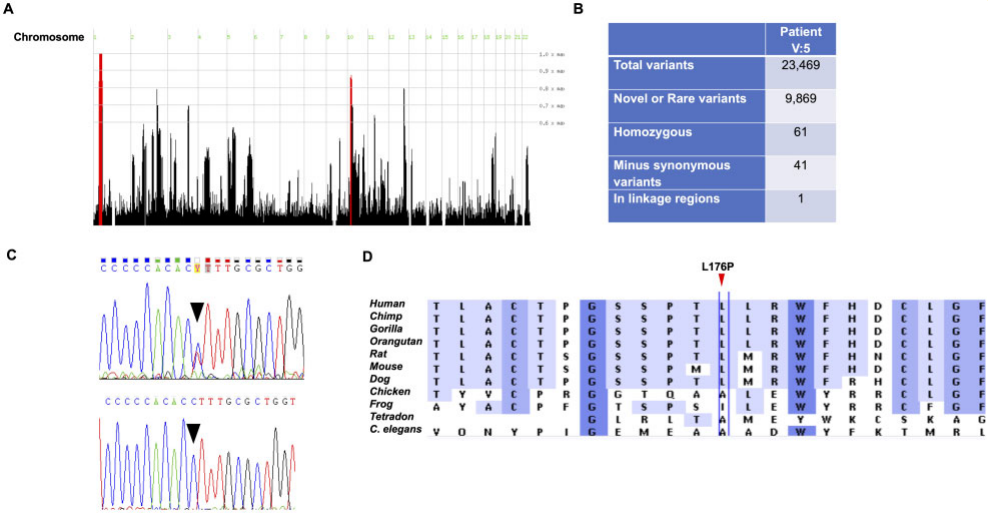
**Mapping and sequence identification of a missense substitution in *HPDL*.** (**A**) Genomewide SNP arrays performed in six affected individuals from family Branches A and B shows areas of extended homozygosity that are shared between all affected individuals and highlighted as red peaks on chromosome 1 (containing *HPDL*) and a smaller region on chromosome 10. (**B**) Initial whole-exome sequencing analysis in patient V: 5 revealed 41 candidate homozygous rare and novel sequence variants through filtering steps and only one of these variants mapped to the chromosome 1 candidate region (c.527T>C, p. Leu176Pro in *HPDL*). (**C**) Sanger sequencing traces showing the *HPDL* missense substitution. Upper panel shows mother of Branch A (IV: 2) in a heterozygous state (T/C) and lower panel the affected individual V: 1 in a homozygous state (C/C). (**D**) A comparison of sequence alignments of *HPDL* in 11 species showing the conservation of the p. Leu176 amino acid position where the variant p. Leu176Pro occurs.

To investigate the evidence of a single genetic cause for the autosomal recessive spastic cerebral palsy segregating in Branches A and B, we performed autozygosity mapping using Affymetrix SNP gene arrays in six affected individuals (V: 1, V: 2, V: 5, V: 7, V: 8) from Branch A and V: 10 from Branch B. Analysis using homozygosityMapper revealed two areas of extended homozygosity shared between all affected individuals. A 551 761-bp region on chromosome 10 between SNPs rs2036919 (chr10:23,636,106, GRCh37) and rs11013778 (chr10:24,187,867, GRCh37) and a much larger 17 596 867 bp region on chromosome 1 between SNP markers rs1046988 (chr1:40,219,065, GRCh37) and rs6687842 (chr1:57,815,932, GRCh37) (Fig. 2A).

To identify a causative pathogenic variant, we proceeded with exome sequencing in one affected individual (V: 5) from Branch A. Exome sequencing revealed 23 469 variations of which 9869 were novel or rare (<0.01) sequence changes. At the time of initial analysis (May 2011; https://etheses.bham.ac.uk//id/eprint/3068/) comparisons with dbSNP build 134, the 1000 Genomes Project database (The 1000 Genomes Project Consortium), Exome Variant Server (http://evs.gs.washington.edu/EVS/) and our in-house dataset, identified 61 homozygous variants in the affected individual (Fig. 2B). Of these, one homozygous non-synonymous missense variant (c.527 T > C; p. Leu176Pro, rs773333490) mapped to one of the identified regions of extended homozygosity on chromosome 1p34.1 in the *HPDL* gene (Fig. 2B). To verify this candidate mutation, Sanger sequencing/exome sequencing was performed on the DNA of three available members of the family from Branch A (IV: 2; V: 1; V: 5) and was found to segregate with the disease (Fig. 2C and Supplementary Table 1). The unaffected parent (IV: 2) was heterozygous for the variant (T/C) and the two affected individuals (V: 1 and V: 5) were homozygous for the mutant allele (C/C). Interrogation of genomic databases at the time of the initial exome sequencing (May 2011) did not identify homozygous individuals in any control data sets (see above). Subsequently, further exome sequencing undertaken in two affected individuals from Branch B (V: 10 and V: 11) and three unaffected family members (IV: 3, IV: 4 and V: 12) demonstrated that V: 10 and V: 11 were homozygous for the mutant allele and the unaffected parents and sibling were heterozygous for the mutant allele either directly (IV: 4), or inferred from flanking SNPs (IV: 3 and V: 12). Finally, three affected individuals (V: 2; V: 7; V: 8) from Branch A in whom DNA was not available for sequencing were inferred (from flanking homozygous SNPs) to be homozygous for the mutant allele (C/C) from the SNP array data (Supplementary Tables 1 and Table 3). Overall, these findings were consistent with the candidate pathogenic *HPDL* variant segregating with the disease in both branches of the family. Subsequent to the initial exome sequencing analysis and identification of the *HPDL* variant ((c.527 T > C; p. Leu176Pro, rs773333490), independent analysis of the exome-sequencing results for two affected individuals (V: 10 and V: 11) in Branch B also prioritized this variant as the best candidate mutation within the autozygous segment.

We inferred identity by descent to estimate familial relationships and calculated pairwise PIHAT between six family members who underwent exome sequencing (Supplementary Methods). This confirmed parental relatedness between IV: 3 and IV: 4 (PIHAT 0.15) and that V: 5 from Branch A was closely related to the two affected individuals in Branch B (V: 10 and V: 11; PIHAT 0.28 and 0.26, respectively).

*In silico* variant pathogenicity prediction tools categorized the missense substitution as possibly_damaging in Polyphen (http://genetics.bwh.harvard.edu/pph2/) and deleterious in SIFT (https://sift.bii.a-star.edu.sg). The affected Leucine residue (ancestral T allele) is conserved across multiple species (Fig. 2D). The variant was heterozygous in one individual of South Asian origin in gnomAD (https://gnomad.broadinstitute.org, giving an allele frequency of 1 in 244 766, and never reported in a homozygous state). Variant classification according to ACMG criteria and assigning moderate evidence for pathogenicity for the results of segregation of the c.527 T > C; p. Leu176Pro substitution in both branches of the family resulted in a likely pathogenic classification for this variant (Evidence for pathogenicity PM1 PM2 PP1; Intervar, Li and Wang, 2017).

## Discussion

The identification of a genetic cause for cerebral palsy has important implications, enabling clarification of the recurrence risk and risks to relatives, provides parents with an explanation for their child’s disorder and may occasionally identify a treatable condition (Moreno-De-Luca et al., 2012). Genetic causes of cerebral palsy are extremely heterogeneous and may overlap with those from other neurological disorders. Clinically, cerebral palsy has been sub-divided into sub-types including spastic, athetoid, ataxic and mixed types but the clinical diagnosis of spastic cerebral palsy and hereditary spastic parapareis (HSP) or of ataxic cerebral palsy and hereditary cerebellar ataxias may be blurred and both HSP and hereditary cerebellar ataxias are highly genetically heterogeneous (Fahey *et al.*, 2017; Parodi *et al.*, 2018). The key clinical distinction between spastic diplegic cerebral palsy and HSP is the progressive nature of the latter, but this may not be obvious without long-term follow-up and there are previous examples of a molecular diagnosis of HSP being made in children who were initially labelled as having cerebral palsy (Rainier *et al*., 2006).

Previously, a homozygous *GAD1* missense substitution (c. 36 G > C; p. Ser12Cys) was identified in four affected siblings with symmetrical spastic cerebral palsy (Lynex *et al.*, 2004). At that time, the variant was not present in 100 control individuals. Recently (accessed August 2020), this variant was found to have a frequency of 6 in 251 302 alleles (allele frequency = 0.00002388) in gnomAD with no homozygotes reported [in South Asians the allele frequency was 0.0001633 (5 in 30 616)]. However, the *GAD1* variant did not segregate with disease status in Branch B of the large consanguineous kindred in which the *GAD1* variant was identified in Branch A. Therefore, unless there were two separate genetic diagnoses in the kindred, *GAD1* was not the cause of the autosomal recessively inherited spastic cerebral palsy disorder within the kindred. To date, though *GAD1* mutation has been widely cited as a cause of autosomal recessively inherited cerebral palsy (Lynex *et al.*, 2004), this kindred is the only reported example of CPSQ1 (OMIM 603513).

To search for other candidate pathogenic variants, we undertook whole-exome sequencing and identified a single-nucleotide variants, leading to a missense substitution (c.527T>C, p. Leu176Pro) in *HPDL*, which encodes a 371 amino acid protein of unknown function. When we initially detected this variant, the *HPDL* gene had not been linked to human disease but recently, Husain *et al.* (2020) described biallelic variants in *HPDL* in a childhood onset progressive spastic movement disorder with a variable presentation. They reported 13 families containing 17 affected individuals (five kindreds with parental consanguinity) with biallelic *HPDL* mutations (loss of function variants in five individuals from four kindreds). The rare c.527T>C, p. Leu176Pro variant detected in both branches of the family reported here has not been reported previously but other candidate pathogenic missense variants described by Husain *et al.* (2020) were p. Gly50Asp (detected in three kindreds); p. Trp157Arg; p. Leu217Pro; p. Gly250Glu; p. His251Gln; p. Ile266Thr and pTyr287His. *HPDL* encodes the 4-Hydroxyphenylpyruvate Dioxygenase-Like protein that does not currently have a known function. *HPDL* transcript levels are high in the central and peripheral nervous system, a mitochondrial localization signal was predicted in the first 37 amino acids and exogenous HPDL was found to co-localize with mitochondria (Husain *et al.*, 2020). However, fibroblasts and muscle from affected individuals did not show clear evidence of aberrant mitochondrial function or morphology (Husain *et al.*, 2020), and thus further evidence is required before HPDL can be unequivocally placed in the group of HSP genes that affect mitochondrial function (Hensiek *et al.*, 2015).

Though we report a single kindred, the large number of affected individuals (*n* = 7) adds significantly to the number of subjects with HPDL-associated disease currently in the literature (*n* = 17). In the original descriptions of affected children in Branch A, Mitchell and Bundey (1997) described how, after a normal pregnancy, the four affected individuals showed signs in the first year of life and all had a non-progressive symmetrical paraparesis (upper limbs affected in some cases). A degree of learning disability was present in all cases (severe in two) but upper limb ataxia was variably present. By the third decade, affected individuals were wheelchair-dependent with fixed flexion deformities. The additional individuals (V: 8 from Branch A and V: 10 and V: 11 from Branch B) who were diagnosed since Lynex *et al.* (2004) generally showed similar features though prenatal growth retardation was present in one individual. The affected individuals had not been extensively investigated but, when performed, blood lactate levels were normal and brain MRI scans in three individuals were mostly unremarkable though one scan was reported to show demyelination in the medulla. In the series of patients (*n* = 17) reported by  Husain *et al*. (2020), the clinical phenotype was more variable ranging from a severe spastic neurological disorder to uncomplicated hereditary spastic paraparesis. Features present in more than a half of patients included progression of neurological signs, intellectual disability, developmental motor delay, microcephaly and strabismus (which were present in our series except for microcephaly). A sub-group of their cohort presented in the neonatal period with severe disease and acute respiratory failure. In many cases, blood lactate levels were increased (at the time of neurological deterioration but not at routine follow-ups) and MRI scans showed deficiency in myelination in most cases (present in one of our series). Thus, the phenotype of autosomal recessive HPDL-related neurological disorder is variable and ranges from a severe congenital-onset disorder without neurological development to a milder later-onset hereditary spastic paraparesis phenotype, and the clinical characteristics of the affected individuals in Branch A and Branch B would appear to fall within this spectrum. Though the *GAD1* (c. 36 G > C; p. Ser12Cys) detected previously in Branch A could not account for the disease phenotype in both branches of the kindred, we note that mice homozygous null for *Gad1* die at birth with cleft palate and hypoxia, with omphalocele in about 50% (Asada *et al.*, 1997; Condie *et al.*, 1997; Saito *et al.*, 2010). Furthermore, Chatron *et al*. (2020) have recently reported that biallelic pathogenic (mostly loss of function) variants in *GAD1* were associated with a phenotype of early onset epileptic encephalopathy in 11 individuals from six independent kindreds. Additional, variable, clinical features included cleft palate (64%), omphalocele (18%) and joint contractures (55%). Though it is possible that *GAD1* variant status might modify clinical presentation within the two branches of the family described here, we note that these clinical features, including, epilepsy were not reported within the family.

## Conclusion

In summary, we propose that the evidence for an autosomal recessive spastic cerebral palsy-1 (CPSQ1; OMIM 603513) locus at 2q31.1 resulting from a mutation in the *GAD1* gene is insecure and that the single family reported to have a *GAD1-*related neurological disorder are most likely to have a *HPDL*-related autosomal recessive disorder. Our findings support the contention that biallelic mutations in *HPDL* can present with a variety of clinical phenotypes and the neurological phenotype is progressive. They also underscore the need to consider a genetic etiology in patients labelled as having cerebral palsy.

## Supplementary material

Supplementary material is available at *Brain Communications* online.

## Supplementary Material

fcab002_Supplementary_Data

## Abbreviations

CPSQ1 =: locus for autosomal recessive spastic cerebral palsy-1 (OMIM 603513)
GAD1 =: glutamate decarboxylate-1
HPDL =: 4-Hydroxyphenylpyruvate Dioxygenase Like
HSP =: hereditary spastic paraparesis
OMIM =: Mendelian Inheritance in Man
