# Supplementary material for: Evidence that autosomal recessive spastic cerebral palsy-1 (CPSQ1) is caused by a missense variant in HPDL
Source: Brain Commun. 2021 Jan 28;3(1):fcab002. doi: 10.1093/braincomms/fcab002 (PMC7892364; doi:10.1093/braincomms/fcab002)
Supplement: fcab002_Supplementary_Data [file fcab002_supplementary_data.zip › Supplementary_Table_4.pdf]

Supplemetary Table 4

CHROM	POS	ID	REF	ALT	QUAL	Consequence	IMPACT	SYMBOL
chr1	44806175	rs759039462	GGGCTG	G	6255.13	downstream_gene_variant	MODIFIER	PLK3
chr1	45206192	rs768634679	G	T	8826.54	synonymous_variant	LOW	ZSWIM5
chr1	45327675	rs773333490	T	C	4090.95	missense_variant	MODERATE	HPDL
chr1	45729597	rs768635994	T	C	9100.54	intron_variant	MODIFIER	IPP
chr1	46191982	rs528667376	C	G	3633.66	intron_variant	MODIFIER	POMGNT1
chr1	46643263	rs763191064	A	G	8689.54	synonymous_variant	LOW	ATPAF1
chr1	51825345	rs370502514	C	T	10346.5	synonymous_variant	LOW	NRDC
chr1	56886031	rs147513844	G	T	18991.8	missense_variant	MODERATE	C8A
chr1	120814790	chrchr1bp12081	C	T	9890.93	intron_variant	MODIFIER	NBPF26
chr1	150230711	rs782288239	G	GA	7673.89	intron_variant	MODIFIER	ANP32E
chr10	17849701	rs606231248	G	A	37253.9	missense_variant	MODERATE	MRC1
chr10	50210866	chrchr10bp5021	T	C	37082.9	synonymous_variant	LOW	ASAH2
chr11	36271570	rs543326317	G	GACTT	20957	downstream_gene_variant	MODIFIER	COMMD9
chr11	46317341	rs577462367	C	T	3885.01	intron_variant	MODIFIER	CREB3L1
chr11	46726097	rs138260543	C	T	11664	synonymous_variant	LOW	F2
chr11	55665389	rs201388016	G	A	10138	missense_variant	MODERATE	OR4C6
chr11	55827767	rs374078582	T	C	7663.01	synonymous_variant	LOW	OR5L2
chr11	61342488	rs200763152	G	A	13041	missense_variant	MODERATE	TKFC
chr11	63095960	rs184268615	T	A	4177.17	intron_variant	MODIFIER	SLC22A24
chr11	63671373	rs528988637	AGAG	A	5656.05	5_prime_UTR_variant	MODIFIER	ATL3
chr11	65035224	rs761464534	G	A	1165.01	intron_variant	MODIFIER	SNX15
chr11	66334581	rs780118610	C	A	4516.16	synonymous_variant	LOW	RIN1
chr11	66704877	rs144746828	C	T	14035	missense_variant	MODERATE	SPTBN2
chr11	68780769	rs545149834	A	G	11691	intron_variant	MODIFIER	CPT1A
chr11	69054039	rs150671549	C	T	5615.01	missense_variant	MODERATE	TPCN2
chr11	69773347	rs752416500	G	C	10430	missense_variant	MODERATE	FGF4
chr12	95062595	rs149368820	C	T	16630	synonymous_variant	LOW	NR2C1
chr19	39891610	rs2260134	A	T	9890.93	intron_variant	MODIFIER	FCGBP
chr19	39891613	rs78638893	C	T	9666.15	intron_variant	MODIFIER	FCGBP

chr19	39906217	chrchr19bp3990	T	C	4204.93	synonymous_variant	LOW	FCGBP
chr19	39906221	rs782328768	C	T	4024.93	missense_variant	MODERATE	FCGBP
chr19	39906229	chrchr19bp3990	C	T	3394.93	synonymous_variant	LOW	FCGBP
chr19	39906242	rs782103783	T	C	9912.93	missense_variant	MODERATE	FCGBP
chr4	186628707	rs568366597	C	T	17546	synonymous_variant	LOW	FAT1

HGVSc	HGVSp	MAX_AF	MAX_AF_P	V:10		V:11	
				GT	DP	GT	DP
		0.000318	gnomAD_S	1/1	83	1/1	100
ENST00000359600.5:c.159C>A	ENSP00000352614.5:p.Val53%3D	3.46E-05	gnomAD_S	1/1	136	1/1	189
ENST00000334815.4:c.527T>C	ENSP00000335060.3:p.Leu176Pro	3.28E-05	gnomAD_S	1/1	54	1/1	62
ENST00000396478.3:c.880+17A>G		7.23E-05	gnomAD_S	1/1	31	1/1	53
ENST00000371992.1:c.1539+116G>C		0.001	EUR&SAS	1/1	57	1/1	51
ENST00000576409.5:c.792T>C	ENSP00000460964.1:p.Tyr264%3D	0.000163	gnomAD_S	1/1	80	1/1	127
ENST00000354831.11:c.1182G>A	ENSP00000346890.7:p.Lys394%3D	0.002813	gnomAD_S	1/1	79	1/1	88
ENST00000361249.3:c.960G>T	ENSP00000354458.3:p.Glu320Asp	0.006205	gnomAD_S	1/1	187	1/1	234
ENST00000620612.5:c.1878-39C>T		0.004572	gnomAD_E	1/1	55	1/1	118
ENST00000583931.5:c.205-19dup		0.000235	AA	1/1	40	1/1	49
ENST00000569591.2:c.1186G>A	ENSP00000455897.1:p.Gly396Ser	0.000138	gnomAD_A	1/1	109	1/1	139
ENST00000395526.8:c.1371A>G	ENSP00000378897.3:p.Ala457%3D	0.000129	gnomAD_S	1/1	214	1/1	231
		0.0051	SAS	1/1	126	1/1	154
ENST00000621158.4:c.1132-20C>T		0.0041	SAS	1/1	47	1/1	45
ENST00000311907.9:c.798C>T	ENSP00000308541.5:p.Asp266%3D	0.0051	SAS	1/1	98	1/1	144
ENST00000641251.1:c.223G>A	ENSP00000493334.1:p.Val75Ile	0.0051	SAS	1/1	102	1/1	107
ENST00000378397.1:c.549T>C	ENSP00000367650.1:p.Pro183%3D	0.0051	SAS	1/1	55	1/1	64
ENST00000394900.7:c.683G>A	ENSP00000378360.3:p.Arg228Gln	0.003	EUR	1/1	129	1/1	167
ENST00000612278.4:c.1070+31A>T		0.003392	gnomAD_S	1/1	48	1/1	74
ENST00000398868.7:c.-41_-39del		0.0072	SAS	1/1	36	1/1	55
ENST00000377244.7:c.520+18G>A		0.000265	gnomAD_S	1/1	16	1/1	13
ENST00000311320.8:c.1218G>T	ENSP00000310406.4:p.Leu406%3D	0.000272	gnomAD_S	1/1	41	1/1	59
ENST00000533211.5:c.2399G>A	ENSP00000432568.1:p.Arg800Gln	0.000233	EA	1/1	121	1/1	157
ENST00000265641.9:c.1353-24T>C		0.0031	SAS	1/1	142	1/1	175
ENST00000294309.7:c.116C>T	ENSP00000294309.3:p.Ala39Val	0.00871	gnomAD_S	1/1	63	1/1	56
ENST00000168712.2:c.583C>G	ENSP00000168712.1:p.Pro195Ala	0.000195	gnomAD_S	1/1	76	1/1	141
ENST00000333003.9:c.198G>A	ENSP00000333275.4:p.Pro66%3D	0.003092	gnomAD_N	1/1	197	1/1	249
ENST00000616721.5:c.3603+33T>A		0.005882	gnomAD_C	1/1	31	1/1	37
ENST00000616721.5:c.3603+30G>A		0.005373	gnomAD_C	1/1	36	1/1	41

ENST00000616721.5:c.107A>G	ENSP00000481056.2:p.Glu36%3D	0.000154	gnomAD_A	1/1	11	1/1	12
ENST00000616721.5:c.103G>A	ENSP00000481056.2:p.Arg35His	0.007639	gnomAD_N	1/1	10	1/1	11
ENST00000616721.5:c.95G>A	ENSP00000481056.2:p.Ser32%3D	0.000501	gnomAD_E	1/1	9	1/1	9
ENST00000616721.5:c.82A>G	ENSP00000481056.2:p.Glu28Gly	9.88E-05	gnomAD_E	1/1	36	1/1	46
ENST00000441802.6:c.4380G>A	ENSP00000406229.2:p.Lys1460%3D	0.001	SAS	1/1	273	1/1	279
