# Supplementary material for: Evidence that autosomal recessive spastic cerebral palsy-1 (CPSQ1) is caused by a missense variant in HPDL
Source: Brain Commun. 2021 Jan 28;3(1):fcab002. doi: 10.1093/braincomms/fcab002 (PMC7892364; doi:10.1093/braincomms/fcab002)
Supplement: fcab002_Supplementary_Data [file fcab002_supplementary_data.zip › Supplementary_Material.docx]

**Supplementary Material**

**Evidence that autosomal recessive spastic cerebral palsy-1 (CPSQ1) is caused by a missense variant in *HPDL***

Neil V Morgan (1), Bryndis Yngvadottir (2), Mary O’Driscoll (3), Graeme R Clark (2), Diana Walsh (4), Ezequiel Martin (2,5), Louise Tee (6), Evan A Reid (2, 7), Hannah V Titheradge (3), Eamonn R Maher (2)

**Contents**

1. Supplementary Methods
2. Supplementary Table 1
3. Supplementary Figure 1
4. Supplementary Figure 2
5. Supplementary Table 2
6. Supplementary Table 3
7. Supplementary Table 4

Supplementary Methods

**Exome Sequencing and Bioinformatics Analysis for Branch B participants**

Exome sequencing was performed in 5 individuals (IV:3; IV:4; V:10; V:11; V:12), using the Nextera Rapid Capture Enrichment assay for V:10 and V:11 and the Nextera DNA Exome Enrichment assay for V:3, V:4 and V:12. The samples were sequenced on Illumina’s HiSeq 4000 platform with 150bp paired end reads. Raw Illumina BCL files were demultiplexed and converted to fastq format using Illumina’s bcl2fastq 2.19, which also trimmed from the reads the indexes and the adaptors used for sequencing. All sample pairs were aligned to the hg38 version of the reference human genome using bwa 0.7.15 in alt contig aware mode as described by the authors (Li and Durbin, 2009). The generated SAM file was compressed into a BAM file and sorted by genomic position using samtools 1.3.1 (Li *et al.*, 2009). The sorted BAM files were subject to Base Quality Score Recalibration and Indel Realignment followed by variant calling using the Haplotype Caller algorithm as specified in the Genome-Analysis Toolkit (GATK version 3.8 (McKenna *et al.*, 2010)) best practices (DePristo *et al.*, 2001; Auwera *et al.,* 2014). The only default parameter from best practices that was altered was the threshold for mapping quality (mapQ) during variant calling which was set to 30. The resulting VCF files were filtered for a minimum depth of 20 reads and a Genotype Quality (GQ) of 30 using vcftools 0.1.15 (Danecek *et al.*, 2011). VCF files were then annotated with ANNOVAR (Wang et al., 2010) using all default databases available for hg38.

Variants were then filtered based on the following exclusion criteria. A rare variant cut off or minor allele frequency (MAF) of <0.01 in each dataset was used and synonymous and intron variants +/- 5 base pairs away from the exon-intron boundaries were excluded. Non-shared variants between the same affected family members were also eliminated. Following exclusion of variants based on these criteria*, in silico* pathogenicity prediction tools were employed for further analysis.

**Identity by descent (IBD)**

We used PLINK (version 1.90, http://pngu.mgh.harvard.edu/purcell/plink/, Purcell et al (2007)) to infer familial relationships (IBD) by calculating pairwise PIHAT values between all individuals in Branch B (IV:3, IV:4, V:10, V:11 and V:12) and one individual in Branch A (V:5). To this end we filtered out variants with missing call rates above 0.01, as well variants which have a Hardy-Weinberg equilibrium exact test p-value below 0.000005 and variants with minor allele frequency below 0.05.

**References**

Auwera, G. A. Van Der et al. From FastQ data to high confidence varant calls: the Genome Analysis Toolkit best practices pipeline. Curr Protoc Bioinformatics vol. 11 (2014). PMID: 25431634

Danecek, P. et al. The variant call format and VCFtools. Bioinformatics 27, 2156–2158 (2011). PMID: 21653522

DePristo, M. a. et al. A framework for variation discovery and genotyping using next- generation DNA sequencing data. Nat Genet 43, 491–498 (2011). PMID: 21478889

Li, H. & Durbin, R. Fast and accurate short read alignment with Burrows-Wheeler transform. Bioinformatics 25, 1754–1760 (2009). PMID: 20080505

Li, H. et al. The Sequence Alignment/Map format and SAMtools. Bioinformatics 25, 2078–2079 (2009). PMID: 19505943

McKenna, A. et al. The Genome Analysis Toolkit: A MapReduce framework for analyzing next-generation DNA sequencing data. Genome Res. 20, 1297–1303 (2010). PMID: 20644199

Purcell, S. et al. PLINK: a toolset for whole-genome association and population-based linkage analysis. American Journal of Human Genetics. 81(3): 559–575. (2007) PMID: 17701901

Wang K, Li M, & Hakonarson H. ANNOVAR: functional annotation of genetic variants from high-throughput sequencing data. Nucleic Acids Res; 38(16):e164. (2010) PMID: 20601685

**Supplementary Table 1:** Summary of genotyping results for HPDL variant (c.527 T>C; p.Leu176Pro, rs773333490) in family members in who sufficient DNA for testing was available

|  | Branch A | | | | | | | Branch B | | | | |
| --- | --- | --- | --- | --- | --- | --- | --- | --- | --- | --- | --- | --- |
|  | Father | Mother | Child 1 | Child 2 | Child 5 | Child 7 | Child 8 | Father | Mother | Child 1 | Child 2 | Child 3 |
| Individual ID | IV:1 | IV:2 | V:1 | V:2 | V:5 | V:7 | V:8 | IV:3 | IV:4 | V:10 | V:11 | V:12 |
| Clinical Status | Unaffected | Unaffected | Affected | Affected | Affected | Affected | Affected | Unaffected | Unaffected | Affected | Affected | Unaffected |
| SNP Array results^1^ |  |  | Predicted homozygous for *HPDL* c.527 T>C by SNP array data | Predicted homozygous for *HPDL* c.527 T>C by SNP array data | Predicted homozygous for *HPDL* c.527 T>C by SNP array data | Predicted homozygous for *HPDL*  c.527 T>C by SNP array data | Predicted homozygous for *HPDL* c.527 T>C by SNP array data |  |  | Predicted homozygous for *HPDL* c.527 T>C by SNP array data |  |  |
| Exome sequencing ^2^ |  |  |  |  | Homozygous for *HPDL*  c.527 T>C by exome sequencing |  |  | Heterozygous by SNPs flanking the HPDL gene by exome sequencing (poor coverage of the *HPDL*  c.527 T>C variant) | Heterozygous *HPDL* c.527 T>C by exome sequencing | Homozygous *HPDL*  c.527 T>C by exome sequencing; Predicted homozygous by SNP array data | Homozygous *HPDL*  c.527 T>C by exome sequencing; Predicted homozygous by SNP array data | Heterozygous by SNPs flanking the HPDL gene by exome sequencing (poor coverage of the *HPDL* c.527 T>C variant) |
| Sanger requencing results |  | Heterozygous for *HPDL* c.527 T>C by Sanger sequencing | Homozygous for *HPDL*  c.527 T>C by Sanger sequencing |  | Homozygous for *HPDL*  c.527 T>C by Sanger sequencing |  |  |  |  |  |  |  |

^1^ :see supplementary figure 2

^2^ :see supplementary table 2

**Supplementary Figure 1:** results of microsatellite genotyping and *GAD1* variant analysis in Branch A and Branch B

**Supplementary Figure 2**: Results of Affymetrix SNP 5 array genotyping showing the boundaries of the homozygous extended region on chromosome 1 containing *HPDL* for 6 affected individuals from Branch A (V:1, V:2, V:5, V:7, V:8) and B (V:10). The minimal candidate region of overlap between the 6 genotyped affected patients is shown, a 17,596,867 bp region on chromosome 1 between SNP markers rs1046988 (chr1:40,219,065 , GRCh37) (panel A) and rs6687842 (chr1:57,815,932, GRCh37) (panel B), both highlighted in red. Homozygous SNP calls are shaded in red, heterozygous SNP calls in pink or blue and no calls shown in grey. Homozygous (minimal candidate region) is shown by borderlines. Larger homozygous region is not shown due to size but contains the *HPDL* gene at genomic position 45,792,545 - 45,794,347 bp. Genomic build shown is version 37.


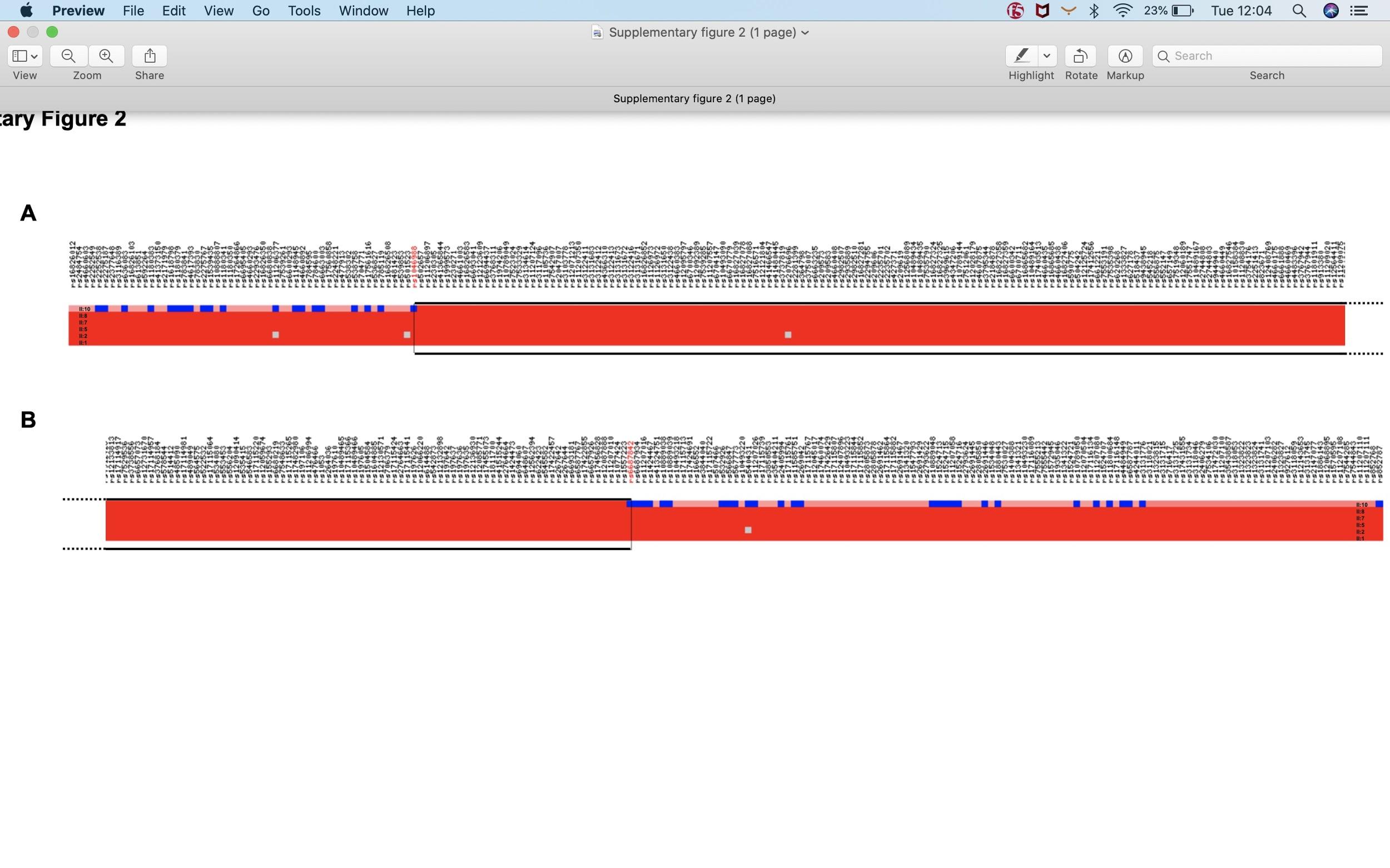


**Supplementary Table 2:** This table contains the genotypes called from whole exome sequencing of three affected individuals (V:5, V:10, V:11), and three unaffected individuals (IV:3, IV:4 (parents of V:10, V:11 and V:12) and V:12 (sibling of V:10 and V:11) for informative (IV:3 and IV:4 both heterozygous) SNPs flanking the HPDL variant of interest ((rs773333490, highlighted in yellow in the table). We filtered out variants with missing data in any individual in Branch B. Grey cells depict missing data in V:5 (from Branch A). Green cells depict SNPs where affected individuals (V:5, V:10 and V:11) were homozygous and unaffected individuals were heterozygous (IV:3, IV:4 and IV:12). A shared minimal region of autozygosity (5,753,368 bp between markers rs117337557 and rs785467 (shaded blue)) common to the three affected individuals is apparent and this region includes the causative variant (yelow) which has inadequate coverage for IV:3 and IV:12. However the flanking SNP genotypes are consistent with the IV:3 being homozygous and IV:12 being heterozygous for the HPDL c.527 T>C; p.Leu176Pro, rs773333490 variant.

Key:

Genotypes (GT) are reported for each sample along with the read depth at each position for the sample (DP).

GT are coded as 0/0 (homozygous for the reference allele), 1/1 (homozygous for the alternative allele), 0/1 (heterozygous), and ./. (missing).

**Supplementary Table 3:** This table contains the genotypes from the genomewide SNP array v5.0 of six affected individuals from Branch A (V:1, V:2, V:5, V:7, V:8) and Branch B (V:10),  in the autozygous region on chromosome 1 containing the *HPDL* gene. Genotypes are indicated as homozygous (AA, BB (highlighted in green)) or heterozygous (AB). The minimal autozygous region is shaded in blue as determined by individual V:10. The number of consecutive homozygous SNPs is shown in brackets after the SNP genotype. The *HPDL* gene at genomic position 45,792,545 - 45,794,347 bp. Genomic build shown is version 37.

**Supplementary Table 4:** This table contains all rare variants that were homozygous for the alternative allele (1/1) in both affected individuals V:10 and V:11 identified by exome sequencing (WES). We filtered out variants with that had a maximum allele frequency (MAX-AF) higher than 0.01, which is based on the highest reported allele frequency observed in any population from 1000 genomes, ESP or gnomAD. Variants in the autozygous region containing the HPDL gene and the causative variant (rs773333490) are highlighted in pink in the table. Genotypes (GT) are reported for each sample along with the read depth at each position for the sample (DP). Biallelic germline mutations in C8A (encoding complement component 8, alpha subunit) are associated with complement component 8 deficiency type I which typically presents with recurrent neisserial infections (particularly meningococcus) after age 10 years. There was no evidence to suggest this diagnosis in the family and the *C8A* variant was also homozygous (1/1) in the unaffected sibling (V:12) leaving the HPDL as the best candidate gene.
