# Supplementary material for: Evidence that autosomal recessive spastic cerebral palsy-1 (CPSQ1) is caused by a missense variant in HPDL
Source: Brain Commun. 2021 Jan 28;3(1):fcab002. doi: 10.1093/braincomms/fcab002 (PMC7892364; doi:10.1093/braincomms/fcab002)
Supplement: fcab002_Supplementary_Data [file fcab002_supplementary_data.zip › Supplementary_Table_2.pdf]

CHROM	POS	ID	REF	ALT	QUAL	V:5		V:10		V:11		V:12		IV:4		IV:3	
						GT	DP	GT	DP	GT	DP	GT	DP	GT	DP	GT	DP
chr1	39753393	rs1046988	C	T	1324.2	./.	29	0/1	48	0/1	47	0/0	20	0/1	30	0/1	48
chr1	39760374	rs12094291	G	A	781.03	0/0	34	0/1	18	0/1	17	0/0	13	0/1	20	0/1	28
chr1	39763696	rs2463260	A	G	2882.03	0/0	44	0/1	127	0/1	148	0/0	33	0/1	47	0/1	52
chr1	40302519	rs117337557	G	A	1617.17	./.	0	1/1	15	1/1	32	0/1	14	0/1	20	0/1	19
chr1	40303513	rs74366749	C	G	9791.54	1/1	15	1/1	89	1/1	156	0/1	147	0/1	119	0/1	164
chr1	40311052	rs202027418	C	T	11952.5	1/1	37	1/1	93	1/1	130	0/1	169	0/1	169	0/1	159
chr1	40495777	rs3795348	T	C	9972.54	1/1	56	1/1	106	1/1	74	0/1	102	0/1	122	0/1	139
chr1	40515558	rs3795344	G	A	12691.5	1/1	34	1/1	83	1/1	99	0/1	236	0/1	273	0/1	247
chr1	40817247	rs13374844	C	T	17163.5	1/1	9	1/1	70	1/1	116	0/1	180	0/1	190	0/1	205
chr1	40817249	rs13374845	C	T	18923.5	1/1	11	1/1	72	1/1	117	0/1	180	0/1	190	0/1	205
chr1	40819913	rs12117176	G	A	33481.5	1/1	88	1/1	184	1/1	261	0/1	144	0/1	209	0/1	236
chr1	40819919	rs12143503	A	G	33152.5	1/1	79	1/1	187	1/1	246	0/1	131	0/1	185	0/1	226
chr1	41382624	rs4359027	G	A	9904.66	./.	5	1/1	169	1/1	179	0/1	24	0/1	26	0/1	38
chr1	42449856	rs12142723	C	A	2974.54	1/1	30	1/1	22	1/1	26	0/1	8	0/1	14	0/1	13
chr1	42450056	rs12047095	C	G	1375.54	0/0	54	0/0	33	0/0	43	0/1	46	0/1	52	0/1	78
chr1	42450082	rs11210647	T	C	780.54	0/0	54	0/0	33	0/0	43	0/1	19	0/1	36	0/1	49
chr1	42556139	rs12038786	C	T	485.54	0/0	53	0/0	11	0/0	30	0/1	35	0/1	33	0/1	29
chr1	42558042	rs71798684	G	GA	209.13	./.	0	0/0	37	0/0	37	0/1	12	0/1	9	0/1	6
chr1	42577032	rs12049046	C	T	1327.54	0/0	42	0/0	37	0/0	34	0/1	41	0/1	55	0/1	44
chr1	42581386	rs12037518	A	G	1134.54	0/0	48	0/0	35	0/0	36	0/1	36	0/1	43	0/1	35
chr1	42747432	rs3738498	A	C	3681.54	1/1	45	1/1	18	1/1	27	0/1	13	0/1	22	0/1	27
chr1	42750259	rs11581921	C	T	13512.5	1/1	28	1/1	138	1/1	197	0/1	134	0/1	120	0/1	155
chr1	42755516	rs3738502	C	T	14564.5	1/1	50	1/1	135	1/1	166	0/1	103	0/1	127	0/1	149
chr1	42837300	rs1466548	C	G	3128.54	1/1	24	1/1	21	1/1	30	0/1	11	0/1	15	0/1	13
chr1	42840082	rs12034000	G	A	28975.5	1/1	212	1/1	278	1/1	363	0/1	107	0/1	148	0/1	163
chr1	42927574	rs11282849	G	GGGT	3177.49	0/0	26	0/0	35	0/0	36	0/1	66	0/1	60	0/1	80
chr1	43569801	rs943513	G	A	4057.54	1/1	19	1/1	38	1/1	62	0/1	13	0/1	13	0/1	14
chr1	43605020	rs17371903	A	G	5561.54	1/1	47	1/1	34	1/1	56	0/1	31	0/1	29	0/1	39
chr1	43605550	rs631248	G	A	7254.54	1/1	16	1/1	71	1/1	91	0/1	111	0/1	78	0/1	141
chr1	43612713	rs499257	T	C	9488.54	1/1	38	1/1	111	1/1	114	0/1	62	0/1	56	0/1	68
chr1	43613740	rs603542	T	C	8743.54	1/1	41	1/1	73	1/1	128	0/1	73	0/1	70	0/1	54

chr1	43617439	rs571862	C	T	7994.54	1/1	71	1/1	47	1/1	62	0/1	61	0/1	73	0/1	79
chr1	43617836	rs641365	T	C	5656.54	1/1	30	1/1	36	1/1	85	0/1	55	0/1	53	0/1	82
chr1	43617848	rs641351	G	A	5629.54	1/1	33	1/1	33	1/1	81	0/1	53	0/1	51	0/1	81
chr1	43619068	rs1143701	C	T	7562.54	1/1	60	1/1	72	1/1	72	0/1	40	0/1	51	0/1	41
chr1	43621160	rs1143702	C	T	7275.54	1/1	21	1/1	74	1/1	61	0/1	81	0/1	67	0/1	81
chr1	44130707	rs34057178	CCAA	C	7112.5	0/0	80	0/0	34	0/0	34	0/1	150	0/1	125	0/1	151
chr1	44220650	rs12419	T	C	2309.54	0/0	19	0/0	41	0/0	33	0/1	80	0/1	115	0/1	89
chr1	44775702	rs3806398	G	A	2742.54	1/1	23	1/1	16	1/1	32	0/1	18	0/1	13	0/1	23
chr1	44802710	rs12404160	G	A	2402.54	1/1	27	1/1	10	1/1	22	0/1	16	0/1	16	0/1	16
chr1	44827194	rs2295996	G	A	18191.5	1/1	44	1/1	141	1/1	221	0/1	239	0/1	250	0/1	319
chr1	44827846	rs7525308	A	G	8670.54	1/1	17	1/1	108	1/1	137	0/1	51	0/1	53	0/1	67
chr1	44828484	rs2273940	T	C	9084.54	1/1	16	1/1	102	1/1	115	0/1	66	0/1	104	0/1	98
chr1	44841834	rs3795720	G	A	11686.5	1/1	55	1/1	93	1/1	111	0/1	162	0/1	155	0/1	184
chr1	44874610	rs9988451	C	T	3247.55	1/1	26	1/1	34	1/1	29	0/1	6	0/1	17	0/1	6
chr1	44879774	rs3738248	T	A	13883.5	1/1	30	1/1	138	1/1	195	0/1	142	0/1	117	0/1	142
chr1	45003483	rs11548990	C	T	5572.54	1/1	33	1/1	44	1/1	66	0/1	37	0/1	62	0/1	54
chr1	45003914	rs41312020	G	A	3805.54	1/1	31	1/1	30	1/1	44	0/1	23	0/1	19	0/1	42
chr1	45013827	rs2234480	G	C	13317.5	1/1	62	1/1	103	1/1	171	0/1	96	0/1	107	0/1	148
chr1	45014037	rs2228084	A	G	21636.5	1/1	50	1/1	185	1/1	200	0/1	245	0/1	326	0/1	374
chr1	45327675	rs773333490	T	C	4090.95	1/1	13	1/1	54	1/1	62	./.	4	0/1	15	./.	8
chr1	45330597	rs3219493	G	C	3262.66	./.	6	0/0	36	0/0	33	0/1	128	0/1	129	0/1	143
chr1	45332883	rs3219487	T	C	8631.54	0/0	59	0/0	36	0/0	37	0/1	304	0/1	315	0/1	363
chr1	45499668	rs7546268	T	C	879.14	./.	1	0/0	31	0/0	39	0/1	24	0/1	21	0/1	24
chr1	45613182	rs2230658	A	G	8527.54	1/1	46	1/1	78	1/1	106	0/1	81	0/1	94	0/1	108
chr1	45613212	rs2230659	C	T	9267.54	1/1	49	1/1	85	1/1	110	0/1	100	0/1	111	0/1	134
chr1	45616288	rs2253862	C	T	6541.54	1/1	46	1/1	73	1/1	75	0/1	35	0/1	42	0/1	49
chr1	45616759	rs2991988	C	A	6165.54	1/1	44	1/1	64	1/1	58	0/1	31	0/1	22	0/1	45
chr1	45618034	rs41310432	C	G	5630	./.	3	1/1	69	1/1	106	0/1	50	0/1	58	0/1	61
chr1	45620405	rs3014246	C	T	4438.54	1/1	60	1/1	33	1/1	20	0/1	27	0/1	24	0/1	18
chr1	45620657	rs11590549	C	T	3027.54	1/1	25	1/1	17	1/1	28	0/1	29	0/1	28	0/1	6
chr1	45621905	rs3014242	G	A	3528.54	1/1	17	1/1	33	1/1	55	0/1	26	0/1	16	0/1	37
chr1	45622207	rs3014241	A	G	6135.54	1/1	27	1/1	14	1/1	20	0/1	110	0/1	104	0/1	120
chr1	45623553	rs3014240	C	G	5325.78	./.	4	1/1	63	1/1	72	0/1	42	0/1	80	0/1	71

chr1	45628355	rs1250	A	G	3867.54	0/0	35	0/0	35	0/0	34	0/1	84	0/1	137	0/1	120
chr1	45629600	rs1135812	T	C	4240.54	0/0	42	0/0	40	0/0	39	0/1	157	0/1	159	0/1	202
chr1	45694588	rs6658700	C	T	715.54	0/0	12	0/0	35	0/0	38	0/1	22	0/1	21	0/1	29
chr1	45729539	rs139068713	TAAC	T	2138.5	0/0	39	0/0	10	0/0	25	0/1	30	0/1	40	0/1	34
chr1	45729597	rs768635994	T	C	9100.54	1/1	97	1/1	31	1/1	53	0/1	70	0/1	102	0/1	81
chr1	45729703	rs28375469	T	C	2757.54	0/0	47	0/0	33	0/0	43	0/1	97	0/1	104	0/1	123
chr1	45913846	rs4256763	A	G	1004.17	./.	0	0/0	36	0/0	37	0/1	47	0/1	18	0/1	45
chr1	46008230	rs3922886	A	T	745.54	0/0	12	0/0	49	0/0	38	0/1	21	0/1	27	0/1	18
chr1	46010915	rs11211247	T	G	2962.54	0/0	50	0/0	33	0/0	36	0/1	68	0/1	83	0/1	87
chr1	46032703	rs1622208	A	G	5349.54	1/1	35	1/1	51	1/1	62	0/1	29	0/1	48	0/1	53
chr1	46034941	rs17855317	T	C	8911.54	1/1	83	1/1	62	1/1	70	0/1	41	0/1	62	0/1	75
chr1	46055845	rs785468	G	A	13044.5	1/1	123	1/1	72	1/1	99	0/1	80	0/1	99	0/1	119
chr1	46055887	rs785467	A	T	14137.5	1/1	83	1/1	108	1/1	147	0/1	94	0/1	115	0/1	162
chr1	46131824	rs751868703	CT	C	375.02	0/0	124	0/0	44	0/1	68	0/1	33	0/1	30	0/1	34
chr1	46511165	rs1886116	C	T	9463.54	1/1	24	1/1	102	1/1	124	0/1	129	0/1	124	0/1	131
chr1	46594828	rs183190014	T	C	3063.17	./.	0	1/1	45	1/1	55	0/1	31	0/1	18	0/1	28
chr1	46643263	rs763191064	A	G	8689.54	1/1	27	1/1	80	1/1	127	0/1	71	0/1	65	0/1	100
chr1	46813503	rs4646487	C	T	11437.5	1/1	42	1/1	91	1/1	96	0/1	181	0/1	176	0/1	188
chr1	46813886	rs4646489	CCCT	C	7722.5	1/1	79	1/1	32	1/1	31	0/1	32	0/1	21	0/1	31
chr1	46818854	rs3766196	C	A	2949.54	1/1	31	1/1	23	1/1	18	0/1	13	0/1	12	0/1	14
chr1	46932824	rs1126742	A	G	9072.54	1/1	105	1/1	69	1/1	83	0/1	73	0/1	98	0/1	132
chr1	47029933	rs17102977	A	G	4491.54	1/1	42	1/1	33	1/1	35	0/1	11	0/1	16	0/1	21
chr1	47031365	rs11211420	G	A	9379.54	1/1	138	1/1	36	1/1	47	0/1	23	0/1	29	0/1	37
chr1	47106230	rs4926802	C	T	8175	1/1	81	0/1	41	0/1	45	0/1	67	0/1	56	0/1	92
chr1	47106233	rs28463559	C	T	8154	1/1	78	0/1	40	0/1	45	0/1	65	0/1	48	0/1	91
chr1	47140798	rs56258513	C	A	11611.5	1/1	85	1/1	89	1/1	105	0/1	99	0/1	104	0/1	136
chr1	47141609	rs12564525	C	T	10920.5	1/1	72	1/1	103	1/1	164	0/1	44	0/1	52	0/1	38
chr1	47142090	rs2056901	T	C	5219.54	1/1	42	1/1	27	1/1	54	0/1	41	0/1	44	0/1	55
chr1	47142113	rs2056900	G	A	7188.54	1/1	49	1/1	42	1/1	71	0/1	68	0/1	65	0/1	93
chr1	47143311	rs4926581	G	T	5130.54	1/1	19	1/1	49	1/1	69	0/1	48	0/1	48	0/1	57
chr1	47143817	rs10789501	T	C	7609.54	1/1	88	1/1	43	1/1	45	0/1	69	0/1	37	0/1	62
chr1	47144393	rs6661132	A	C	9129.54	1/1	59	1/1	62	1/1	76	0/1	110	0/1	94	0/1	132
chr1	47145926	rs2405599	T	C	14617.5	1/1	227	1/1	42	1/1	52	0/1	67	0/1	80	0/1	124

chr1	47148762	rs4926600	C	T	7202.54	1/1	43	1/1	74	1/1	61	0/1	59	0/1	63	0/1	67
chr1	47189927	rs11801079	C	T	645.54	0/0	14	0/0	38	0/0	40	0/1	26	0/1	29	0/1	51
chr1	48232079	rs61997212	C	T	6921.54	1/1	41	1/1	65	1/1	77	0/1	64	0/1	77	0/1	94
chr1	48298747	rs1056042	C	T	12608.5	1/1	161	1/1	72	1/1	72	0/1	59	0/1	74	0/1	68
chr1	48472019	rs2275309	A	T	1729	./.	3	1/1	11	1/1	35	0/1	8	0/1	14	0/1	20
chr1	50491837	rs1149790	A	T	563.54	0/0	38	0/0	34	0/0	40	0/1	24	0/1	22	0/1	36
chr1	50655526	rs11205753	T	C	8191.54	1/1	62	1/1	67	1/1	87	0/1	37	0/1	56	0/1	70
chr1	51302240	rs375305601	GC	G	469.41	./.	28	0/1	91	0/1	80	0/1	98	0/1	81	0/1	94
chr1	51361249	rs17567	T	C	15398.5	1/1	84	1/1	154	1/1	180	0/1	125	0/1	105	0/1	130
chr1	51402391	rs202067917	C	CT	3424.5	./.	9	1/1	58	1/1	45	0/1	6	0/1	22	0/1	12
chr1	51447088	rs34704431	T	G	14656.5	1/1	94	1/1	153	1/1	203	0/1	78	0/1	59	0/1	71
chr1	51458554	rs6672300	C	T	2489.17	./.	0	1/1	36	1/1	44	0/1	31	0/1	29	0/1	32
chr1	51463832	rs5774101	TA	T	6347.5	1/1	43	1/1	37	1/1	73	0/1	13	0/1	26	0/1	14
chr1	51825345	rs370502514	C	T	10346.5	1/1	69	1/1	79	1/1	88	0/1	58	0/1	95	0/1	83
chr1	52340059	chrchr1bp523	C	T	6231.54	1/1	15	1/1	47	1/1	59	0/1	78	0/1	92	0/1	116
chr1	52784978	rs6588443	G	A	1880.76	0/0	46	0/0	62	0/0	37	0/0	33	0/1	110	0/1	131
chr1	52805553	rs487453	T	C	1117.42	./.	0	0/0	37	0/0	40	0/0	39	0/1	41	0/1	60
chr1	52987968	rs7528565	A	G	284.76	0/0	18	0/0	34	0/0	35	0/0	14	0/1	22	0/1	19
chr1	52988011	rs7516939	C	T	1158.76	0/0	34	0/0	34	0/0	35	0/0	33	0/1	67	0/1	70
chr1	52988042	rs7538935	G	T	2023.76	0/0	43	0/0	34	0/0	35	0/0	33	0/1	117	0/1	121
chr1	53047751	rs11206079	C	T	305.76	./.	9	0/0	35	0/0	41	0/0	10	0/1	12	0/1	21
chr1	53069827	rs17107831	C	T	581.37	./.	2	0/0	26	0/0	24	0/0	20	0/1	16	0/1	42
chr1	53210776	rs1799821	G	A	1755.76	0/0	52	0/0	35	0/0	35	0/0	33	0/1	92	0/1	102
chr1	53247055	rs5174	C	T	1031.76	0/0	47	0/0	34	0/0	34	0/0	36	0/1	55	0/1	65
chr1	53250744	rs3737983	G	A	4883.76	0/0	59	0/0	35	0/0	36	0/0	37	0/1	210	0/1	254
chr1	53266643	rs2297660	G	T	679.76	0/0	68	0/0	36	0/0	35	0/0	44	0/1	56	0/1	45
chr1	53464878	rs943514	G	A	16359.8	1/1	53	1/1	124	1/1	173	1/1	146	0/1	172	0/1	198
chr1	53878609	rs2272928	C	A	7849.76	1/1	42	1/1	44	1/1	67	1/1	65	0/1	66	0/1	73
chr1	53904591	rs2294511	A	T	250.76	0/0	83	0/0	12	0/0	22	0/0	18	0/1	11	0/1	17
chr1	54594551	rs1755591	G	A	4694.76	1/1	20	1/1	43	1/1	52	1/1	29	0/1	34	0/1	32
chr1	54594689	rs1702003	G	A	4351.76	1/1	32	1/1	31	1/1	35	1/1	21	0/1	25	0/1	30
chr1	54597287	rs2304305	G	A	11621.8	1/1	25	1/1	85	1/1	78	1/1	156	0/1	159	0/1	179
chr1	54610440	rs4926650	G	A	10993.8	1/1	97	1/1	61	1/1	69	1/1	77	0/1	74	0/1	106

chr1	54653471	rs11206407	A	G	11881.8	1/1	80	1/1	102	1/1	135	1/1	62	0/1	56	0/1	57
chr1	54653616	rs9332417	G	C	25034.8	1/1	72	1/1	210	1/1	219	1/1	236	0/1	291	0/1	339
chr1	54653842	rs1655519	T	G	2789.76	0/0	78	0/0	51	0/0	69	0/0	84	0/1	139	0/1	156
chr1	54665258	rs2078273	C	T	9914.76	1/1	44	1/1	86	1/1	127	1/1	54	0/1	53	0/1	70
chr1	54665273	rs6703113	G	C	7471.76	1/1	38	1/1	55	1/1	91	1/1	40	0/1	34	0/1	46
chr1	54668814	rs478513	G	A	780.76	0/0	51	0/0	19	0/0	27	0/0	35	0/1	43	0/1	43
chr1	54670856	rs480963	T	C	1871.76	0/0	15	0/0	34	0/0	33	0/0	38	0/1	94	0/1	121
chr1	54716630	rs145266091	A	G	27128.8	1/1	63	1/1	212	1/1	219	1/1	81	0/1	65	0/1	69
chr1	54721252	rs3737825	G	A	1183.76	0/0	33	0/0	45	0/0	40	0/0	38	0/1	48	0/1	62
chr1	54722709	rs11550481	C	T	1960.76	0/0	46	0/0	34	0/0	33	0/0	35	0/1	90	0/1	115
chr1	54722819	rs201524669	CAATT	C	1311.71	0/0	46	0/0	34	0/0	33	0/0	35	0/1	30	0/1	37
chr1	54731476	rs3815226	T	C	6665.76	1/1	31	1/1	35	1/1	60	1/1	69	0/1	42	0/1	66
chr1	54741543	rs41297863	T	C	594.76	0/0	53	0/0	40	0/0	34	0/0	34	0/1	48	0/1	38
chr1	54801172	rs1147984	C	T	7903.76	1/1	11	1/1	31	1/1	31	1/1	111	0/1	129	0/1	142
chr1	54807907	rs9782980	G	T	8621.76	1/1	101	1/1	67	1/1	54	1/1	18	0/1	27	0/1	30
chr1	55005149	rs41297879	T	G	6145.76	1/1	17	1/1	56	1/1	65	1/1	35	0/1	62	0/1	63
chr1	55052188	rs613855	C	G	551.76	0/0	58	0/0	35	0/0	34	0/0	38	0/1	38	0/1	40
chr1	55052210	rs624612	C	G	1929.76	0/0	62	0/0	35	0/0	34	0/0	38	0/1	81	0/1	97
chr1	55052794	rs2495477	A	G	11328.8	1/1	19	1/1	72	1/1	76	1/1	147	0/1	164	0/1	177
chr1	55052855	rs494198	C	A	1891.76	0/0	15	0/0	29	0/0	36	0/0	60	0/1	74	0/1	109
chr1	56753958	rs11404547	G	GA	1455.71	0/0	40	0/0	34	0/0	33	0/0	48	0/1	72	0/1	84
chr1	56755880	rs1774808	G	T	3001.76	0/0	47	0/0	33	0/0	34	0/0	35	0/1	55	0/1	83
chr1	56755881	rs1738401	A	G	3001.76	0/0	47	0/0	33	0/0	34	0/0	35	0/1	55	0/1	83
chr1	56758787	rs857103	G	C	662.76	0/0	14	0/0	33	0/0	33	0/0	35	0/1	42	0/1	64
chr1	56875054	rs652785	C	A	23419.8	1/1	18	1/1	218	1/1	321	1/1	186	0/1	170	0/1	261
chr1	56883734	rs6694643	A	T	1026.76	0/0	27	0/0	32	0/0	25	0/0	33	0/1	33	0/1	59
chr1	56886031	rs147513844	G	T	18991.8	1/1	64	1/1	187	1/1	234	1/1	113	0/1	109	0/1	136
chr1	56951960	rs138791380	G	GGGA	1211.71	0/0	32	0/0	35	0/0	34	0/0	39	0/1	29	0/1	33
chr1	57033602	rs17417626	A	G	17564.8	1/1	36	1/1	163	1/1	208	1/1	138	0/1	106	0/1	127
