# Supplementary material for: Evidence that autosomal recessive spastic cerebral palsy-1 (CPSQ1) is caused by a missense variant in HPDL
Source: Brain Commun. 2021 Jan 28;3(1):fcab002. doi: 10.1093/braincomms/fcab002 (PMC7892364; doi:10.1093/braincomms/fcab002)
Supplement: fcab002_Supplementary_Data [file fcab002_supplementary_data.zip › Supplementary_Table_3.pdf]

Supplementary Table 3

dbsnp	pos on chr 1	**V:10**	**V:8**	**V:7**	**V:5**	**V:2**	**V:1**
rs16826012	39719519	BB (4)	BB (3236)	BB (3236)	BB (3236)	BB (3290)	BB (2800)
rs2484754	39728964	AA (4)	AA (3236)	AA (3236)	AA (3236)	AA (3290)	AA (2800)
rs4660603	39736334	BB (4)	BB (3236)	BB (3236)	BB (3236)	BB (3290)	BB (2800)
rs2252549	39743712	AA (4)	AA (3236)	AA (3236)	AA (3236)	AA (3290)	AA (2800)
rs2252538	39743929	AB (0)	AA (3236)	AA (3236)	AA (3236)	AA (3290)	AA (2800)
rs2275187	39763242	AB (0)	AA (3236)	AA (3236)	AA (3236)	AA (3290)	AA (2800)
rs2377648	39780802	AA (2)	AA (3236)	AA (3236)	AA (3236)	AA (3290)	AA (2800)
rs3116389	39785641	BB (2)	BB (3236)	BB (3236)	BB (3236)	BB (3290)	BB (2800)
rs636083	39821681	AB (0)	BB (3236)	BB (3236)	BB (3236)	BB (3290)	BB (2800)
rs16826103	39821705	AA (3)	AA (3236)	AA (3236)	AA (3236)	AA (3290)	AA (2800)
rs613851	39823866	AA (3)	AA (3236)	AA (3236)	AA (3236)	AA (3290)	AA (2800)
rs592264	39845622	BB (3)	BB (3236)	BB (3236)	BB (3236)	BB (3290)	BB (2800)
rs1180383	39846267	AB (0)	BB (3236)	BB (3236)	BB (3236)	BB (3290)	BB (2800)
rs41373150	39859900	AA (2)	AA (3236)	AA (3236)	AA (3236)	AA (3290)	AA (2800)
rs2171979	39885977	BB (2)	BB (3236)	BB (3236)	BB (3236)	BB (3290)	BB (2800)
rs1618798	39886940	AB (0)	AA (3236)	AA (3236)	AA (3236)	AA (3290)	AA (2800)
rs1180379	39915035	AB (0)	BB (3236)	BB (3236)	BB (3236)	BB (3290)	BB (2800)
rs783841	39922303	AB (0)	AA (3236)	AA (3236)	AA (3236)	AA (3290)	AA (2800)
rs4617393	39940223	AB (0)	AA (3236)	AA (3236)	AA (3236)	AA (3290)	AA (2800)
rs783830	39945177	AA (1)	AA (3236)	AA (3236)	AA (3236)	AA (3290)	AA (2800)
rs2275767	39945297	AB (0)	AA (3236)	AA (3236)	AA (3236)	AA (3290)	AA (2800)
rs1539435	39968824	AB (0)	AA (3236)	AA (3236)	AA (3236)	AA (3290)	AA (2800)
rs10888807	39970705	AA (1)	AA (3236)	AA (3236)	AA (3236)	AA (3290)	AA (2800)
rs1180341	39992808	AB (0)	AA (3236)	AA (3236)	AA (3236)	AA (3290)	AA (2800)
rs1180350	40007013	BB (7)	BB (3236)	BB (3236)	BB (3236)	BB (3290)	BB (2800)
rs17264866	40022777	AA (7)	AA (3236)	AA (3236)	AA (3236)	AA (3290)	AA (2800)
rs6695405	40025080	AA (7)	AA (3236)	AA (3236)	AA (3236)	AA (3290)	AA (2800)
rs4660293	40028180	BB (7)	BB (3236)	BB (3236)	BB (3236)	BB (3290)	BB (2800)
rs2293476	40036847	BB (7)	BB (3236)	BB (3236)	BB (3236)	BB (3290)	BB (2800)
rs16826350	40039611	BB (7)	BB (3236)	BB (3236)	BB (3236)	BB (3290)	BB (2800)
rs6680838	40040635	BB (7)	BB (3236)	BB (3236)	BB (3236)	BB (3290)	BB (2800)
rs11206377	40049104	AB (0)	BB (3236)	BB (3236)	BB (3236)	-3290	BB (2800)
rs7539261	40050503	BB (2)	BB (3236)	BB (3236)	BB (3236)	BB (3290)	BB (2800)
rs6600293	40083819	AA (2)	AA (3236)	AA (3236)	AA (3236)	AA (3290)	AA (2800)
rs1148945	40096053	AB (0)	AA (3236)	AA (3236)	AA (3236)	AA (3290)	AA (2800)
rs4660892	40104584	AB (0)	BB (3236)	BB (3236)	BB (3236)	BB (3290)	BB (2800)
rs784595	40135017	BB (1)	BB (3236)	BB (3236)	BB (3236)	BB (3290)	BB (2800)
rs784600	40139553	AB (0)	BB (3236)	BB (3236)	BB (3236)	BB (3290)	BB (2800)
rs6663003	40143327	AB (0)	BB (3236)	BB (3236)	BB (3236)	BB (3290)	BB (2800)
rs17560858	40145850	AA (4)	AA (3236)	AA (3236)	AA (3236)	AA (3290)	AA (2800)
rs2248221	40152117	AA (4)	AA (3236)	AA (3236)	AA (3236)	AA (3290)	AA (2800)
rs477913	40153141	BB (4)	BB (3236)	BB (3236)	BB (3236)	BB (3290)	BB (2800)
rs538302	40153748	AA (4)	AA (3236)	AA (3236)	AA (3236)	AA (3290)	AA (2800)
rs538758	40155188	AB (0)	AA (3236)	AA (3236)	AA (3236)	AA (3290)	AA (2800)
rs704771	40161013	AA (1)	AA (3236)	AA (3236)	AA (3236)	AA (3290)	AA (2800)
rs17561616	40168865	AB (0)	AA (3236)	AA (3236)	AA (3236)	AA (3290)	AA (2800)
rs536827	40169724	BB (1)	BB (3236)	BB (3236)	BB (3236)	BB (3290)	BB (2800)

rs785119	40178268	AB (0)	AA (3236)	AA (3236)	AA (3236)	AA (3290)	AA (2800)
rs16826508	40179431	AA (4)	AA (3236)	AA (3236)	AA (3236)	AA (3290)	AA (2800)
rs481423	40183922	AA (4)	AA (3236)	AA (3236)	AA (3236)	AA (3290)	AA (2800)
rs539853	40194903	BB (4)	BB (3236)	BB (3236)	BB (3236)	BB (3290)	BB (2800)
rs501523	40216993	BB (4)	BB (3236)	BB (3236)	BB (3236)	-3290	BB (2800)
rs1046988	40219065	AB (0)	BB (3236)	BB (3236)	BB (3236)	BB (3290)	BB (2800)
rs502657	40221855	BB (2385)	BB (3236)	BB (3236)	BB (3236)	BB (3290)	BB (2800)
rs11206897	40264146	AA (2385)	AA (3236)	AA (3236)	AA (3236)	AA (3290)	AA (2800)
rs230305	40283051	AA (2385)	AA (3236)	AA (3236)	AA (3236)	AA (3290)	AA (2800)
rs41365844	40284187	BB (2385)	BB (3236)	BB (3236)	BB (3236)	BB (3290)	BB (2800)
rs1999573	40289812	AA (2385)	AA (3236)	AA (3236)	AA (3236)	AA (3290)	AA (2800)
rs230271	40290597	BB (2385)	BB (3236)	BB (3236)	BB (3236)	BB (3290)	BB (2800)
rs4661003	40290696	AA (2385)	AA (3236)	AA (3236)	AA (3236)	AA (3290)	AA (2800)
rs16826583	40291192	BB (2385)	BB (3236)	BB (3236)	BB (3236)	BB (3290)	BB (2800)
rs6693041	40302463	BB (2385)	BB (3236)	BB (3236)	BB (3236)	BB (3290)	BB (2800)
rs12123609	40303627	AA (2385)	AA (3236)	AA (3236)	AA (3236)	AA (3290)	AA (2800)
rs6694437	40304647	AA (2385)	AA (3236)	AA (3236)	AA (3236)	AA (3290)	AA (2800)
rs3768311	40308017	AA (2385)	AA (3236)	AA (3236)	AA (3236)	AA (3290)	AA (2800)
rs1974206	40308918	BB (2385)	BB (3236)	BB (3236)	BB (3236)	BB (3290)	BB (2800)
rs10789049	40343432	AA (2385)	AA (3236)	AA (3236)	AA (3236)	AA (3290)	AA (2800)
rs7523024	40346001	AA (2385)	AA (3236)	AA (3236)	AA (3236)	AA (3290)	AA (2800)
rs7533329	40346129	AA (2385)	AA (3236)	AA (3236)	AA (3236)	AA (3290)	AA (2800)
rs3134614	40363054	AA (2385)	AA (3236)	AA (3236)	AA (3236)	AA (3290)	AA (2800)
rs11207124	40381453	AA (2385)	AA (3236)	AA (3236)	AA (3236)	AA (3290)	AA (2800)
rs3117096	40389966	BB (2385)	BB (3236)	BB (3236)	BB (3236)	BB (3290)	BB (2800)
rs1105736	40390532	BB (2385)	BB (3236)	BB (3236)	BB (3236)	BB (3290)	BB (2800)
rs7542907	40391752	BB (2385)	BB (3236)	BB (3236)	BB (3236)	BB (3290)	BB (2800)
rs2047007	40427975	BB (2385)	BB (3236)	BB (3236)	BB (3236)	BB (3290)	BB (2800)
rs3103778	40433771	AA (2385)	AA (3236)	AA (3236)	AA (3236)	AA (3290)	AA (2800)
rs12071213	40445667	BB (2385)	BB (3236)	BB (3236)	BB (3236)	BB (3290)	BB (2800)
rs11207350	40491765	AA (2385)	AA (3236)	AA (3236)	AA (3236)	AA (3290)	AA (2800)
rs3122411	40503779	BB (2385)	BB (3236)	BB (3236)	BB (3236)	BB (3290)	BB (2800)
rs3131683	40513710	BB (2385)	BB (3236)	BB (3236)	BB (3236)	BB (3290)	BB (2800)
rs3122412	40515277	BB (2385)	BB (3236)	BB (3236)	BB (3236)	BB (3290)	BB (2800)
rs4366310	40515346	BB (2385)	BB (3236)	BB (3236)	BB (3236)	BB (3290)	BB (2800)
rs3122413	40519523	BB (2385)	BB (3236)	BB (3236)	BB (3236)	BB (3290)	BB (2800)
rs3131673	40522971	BB (2385)	BB (3236)	BB (3236)	BB (3236)	BB (3290)	BB (2800)
rs3131672	40523157	BB (2385)	BB (3236)	BB (3236)	BB (3236)	BB (3290)	BB (2800)
rs3122416	40523328	BB (2385)	BB (3236)	BB (3236)	BB (3236)	BB (3290)	BB (2800)
rs3131671	40524690	BB (2385)	BB (3236)	BB (3236)	BB (3236)	BB (3290)	BB (2800)
rs16826852	40531094	BB (2385)	BB (3236)	BB (3236)	BB (3236)	BB (3290)	BB (2800)
rs1126973	40539076	BB (2385)	BB (3236)	BB (3236)	BB (3236)	BB (3290)	BB (2800)
rs1126972	40539228	AA (2385)	AA (3236)	AA (3236)	AA (3236)	AA (3290)	AA (2800)
rs3131650	40555723	AA (2385)	AA (3236)	AA (3236)	AA (3236)	AA (3290)	AA (2800)
rs3122438	40555802	AA (2385)	AA (3236)	AA (3236)	AA (3236)	AA (3290)	AA (2800)
rs4660383	40559248	AA (2385)	AA (3236)	AA (3236)	AA (3236)	AA (3290)	AA (2800)
rs12095797	40588288	AA (2385)	AA (3236)	AA (3236)	AA (3236)	AA (3290)	AA (2800)
rs6700346	40588548	AA (2385)	AA (3236)	AA (3236)	AA (3236)	AA (3290)	AA (2800)
rs12092389	40593127	BB (2385)	BB (3236)	BB (3236)	BB (3236)	BB (3290)	BB (2800)
rs7539285	40594472	BB (2385)	BB (3236)	BB (3236)	BB (3236)	BB (3290)	BB (2800)

rs11207557	40595283	BB (2385)	BB (3236)	BB (3236)	BB (3236)	BB (3290)	BB (2800)
rs6704147	40595353	AA (2385)	AA (3236)	AA (3236)	AA (3236)	AA (3290)	AA (2800)
rs10493090	40626918	BB (2385)	BB (3236)	BB (3236)	BB (3236)	BB (3290)	BB (2800)
rs6670779	40657449	AA (2385)	AA (3236)	AA (3236)	AA (3236)	AA (3290)	AA (2800)
rs16827039	40665502	AA (2385)	AA (3236)	AA (3236)	AA (3236)	AA (3290)	AA (2800)
rs16827078	40702570	AA (2385)	AA (3236)	AA (3236)	AA (3236)	AA (3290)	AA (2800)
rs16827088	40714034	AA (2385)	AA (3236)	AA (3236)	AA (3236)	AA (3290)	AA (2800)
rs7516571	40733658	BB (2385)	BB (3236)	BB (3236)	BB (3236)	BB (3290)	BB (2800)
rs12121804	40761848	BB (2385)	BB (3236)	BB (3236)	BB (3236)	BB (3290)	BB (2800)
rs41466547	40766542	AA (2385)	AA (3236)	AA (3236)	AA (3236)	AA (3290)	AA (2800)
rs41480445	40766618	BB (2385)	BB (3236)	BB (3236)	BB (3236)	BB (3290)	BB (2800)
rs3737819	40768625	BB (2385)	BB (3236)	BB (3236)	BB (3236)	BB (3290)	BB (2800)
rs2076696	40771973	AA (2385)	AA (3236)	AA (3236)	AA (3236)	-3290	AA (2800)
rs2281399	40771994	BB (2385)	BB (3236)	BB (3236)	BB (3236)	BB (3290)	BB (2800)
rs364798	40799427	AA (2385)	AA (3236)	AA (3236)	AA (3236)	AA (3290)	AA (2800)
rs375007	40799989	AA (2385)	AA (3236)	AA (3236)	AA (3236)	AA (3290)	AA (2800)
rs6663235	40803833	AA (2385)	AA (3236)	AA (3236)	AA (3236)	AA (3290)	AA (2800)
rs209573	40838636	BB (2385)	BB (3236)	BB (3236)	BB (3236)	BB (3290)	BB (2800)
rs209583	40847491	AA (2385)	AA (3236)	AA (3236)	AA (3236)	AA (3290)	AA (2800)
rs4660408	40864296	BB (2385)	BB (3236)	BB (3236)	BB (3236)	BB (3290)	BB (2800)
rs2982507	40865801	AA (2385)	AA (3236)	AA (3236)	AA (3236)	AA (3290)	AA (2800)
rs2935889	40872356	BB (2385)	BB (3236)	BB (3236)	BB (3236)	BB (3290)	BB (2800)
rs2982510	40872623	BB (2385)	BB (3236)	BB (3236)	BB (3236)	BB (3290)	BB (2800)
rs16827281	40876284	BB (2385)	BB (3236)	BB (3236)	BB (3236)	BB (3290)	BB (2800)
rs2294755	40880804	AA (2385)	AA (3236)	AA (3236)	AA (3236)	AA (3290)	AA (2800)
rs209606	40881041	AA (2385)	AA (3236)	AA (3236)	AA (3236)	AA (3290)	AA (2800)
rs2235701	40883095	BB (2385)	BB (3236)	BB (3236)	BB (3236)	BB (3290)	BB (2800)
rs2235702	40883132	BB (2385)	BB (3236)	BB (3236)	BB (3236)	BB (3290)	BB (2800)
rs427371	40883266	AA (2385)	AA (3236)	AA (3236)	AA (3236)	AA (3290)	AA (2800)
rs209619	40904757	AA (2385)	AA (3236)	AA (3236)	AA (3236)	AA (3290)	AA (2800)
rs12568089	40921238	BB (2385)	BB (3236)	BB (3236)	BB (3236)	BB (3290)	BB (2800)
rs10489434	40922062	AA (2385)	AA (3236)	AA (3236)	AA (3236)	AA (3290)	AA (2800)
rs10489435	40922377	AA (2385)	AA (3236)	AA (3236)	AA (3236)	AA (3290)	AA (2800)
rs7535790	40924019	AA (2385)	AA (3236)	AA (3236)	AA (3236)	AA (3290)	AA (2800)
rs16827324	40927959	AA (2385)	AA (3236)	AA (3236)	AA (3236)	AA (3290)	AA (2800)
rs16827325	40930155	BB (2385)	BB (3236)	BB (3236)	BB (3236)	BB (3290)	BB (2800)
rs3969615	40930890	AA (2385)	AA (3236)	AA (3236)	AA (3236)	AA (3290)	AA (2800)
rs4347206	40938751	AA (2385)	AA (3236)	AA (3236)	AA (3236)	AA (3290)	AA (2800)
rs10789144	40955156	AA (2385)	AA (3236)	AA (3236)	AA (3236)	AA (3290)	AA (2800)
rs7512061	40966183	BB (2385)	BB (3236)	BB (3236)	BB (3236)	BB (3290)	BB (2800)
rs12028179	40966458	BB (2385)	BB (3236)	BB (3236)	BB (3236)	BB (3290)	BB (2800)
rs4607875	40975260	BB (2385)	BB (3236)	BB (3236)	BB (3236)	BB (3290)	BB (2800)
rs3795344	40981230	AA (2385)	AA (3236)	AA (3236)	AA (3236)	AA (3290)	AA (2800)
rs2184878	40994050	AA (2385)	AA (3236)	AA (3236)	AA (3236)	AA (3290)	AA (2800)
rs16827358	40999475	AA (2385)	AA (3236)	AA (3236)	AA (3236)	AA (3290)	AA (2800)
rs16827359	41001438	AA (2385)	AA (3236)	AA (3236)	AA (3236)	AA (3290)	AA (2800)
rs6600342	41004021	AA (2385)	AA (3236)	AA (3236)	AA (3236)	AA (3290)	AA (2800)
rs6700711	41005205	BB (2385)	BB (3236)	BB (3236)	BB (3236)	BB (3290)	BB (2800)
rs16865682	41006154	AA (2385)	AA (3236)	AA (3236)	AA (3236)	AA (3290)	AA (2800)
rs10489164	41011140	BB (2385)	BB (3236)	BB (3236)	BB (3236)	BB (3290)	BB (2800)

rs17403513	41030464	AA (2385)	AA (3236)	AA (3236)	AA (3236)	AA (3290)	AA (2800)
rs4660435	41030487	BB (2385)	BB (3236)	BB (3236)	BB (3236)	BB (3290)	BB (2800)
rs16865685	41049437	BB (2385)	BB (3236)	BB (3236)	BB (3236)	BB (3290)	BB (2800)
rs4660438	41050952	BB (2385)	BB (3236)	BB (3236)	BB (3236)	BB (3290)	BB (2800)
rs16827406	41059499	AA (2385)	AA (3236)	AA (3236)	AA (3236)	AA (3290)	AA (2800)
rs590775	41065744	BB (2385)	BB (3236)	BB (3236)	BB (3236)	BB (3290)	BB (2800)
rs504242	41081354	BB (2385)	BB (3236)	BB (3236)	BB (3236)	BB (3290)	BB (2800)
rs12125724	41086224	BB (2385)	BB (3236)	BB (3236)	BB (3236)	BB (3290)	BB (2800)
rs17412556	41088577	AA (2385)	AA (3236)	AA (3236)	AA (3236)	AA (3290)	AA (2800)
rs661221	41093830	BB (2385)	BB (3236)	BB (3236)	BB (3236)	BB (3290)	BB (2800)
rs7553191	41093908	AA (2385)	AA (3236)	AA (3236)	AA (3236)	AA (3290)	AA (2800)
rs7535398	41107239	BB (2385)	BB (3236)	BB (3236)	BB (3236)	BB (3290)	BB (2800)
rs620268	41111071	BB (2385)	BB (3236)	BB (3236)	BB (3236)	BB (3290)	BB (2800)
rs1333827	41111970	BB (2385)	BB (3236)	BB (3236)	BB (3236)	BB (3290)	BB (2800)
rs627178	41127976	BB (2385)	BB (3236)	BB (3236)	BB (3236)	BB (3290)	BB (2800)
rs518437	41132175	AA (2385)	AA (3236)	AA (3236)	AA (3236)	AA (3290)	AA (2800)
rs9438945	41139584	BB (2385)	BB (3236)	BB (3236)	BB (3236)	BB (3290)	BB (2800)
rs545218	41140985	AA (2385)	AA (3236)	AA (3236)	AA (3236)	AA (3290)	AA (2800)
rs556875	41154243	BB (2385)	BB (3236)	BB (3236)	BB (3236)	BB (3290)	BB (2800)
rs552147	41154813	BB (2385)	BB (3236)	BB (3236)	BB (3236)	BB (3290)	BB (2800)
rs657149	41158158	AA (2385)	AA (3236)	AA (3236)	AA (3236)	AA (3290)	AA (2800)
rs7519348	41160204	BB (2385)	BB (3236)	BB (3236)	BB (3236)	BB (3290)	BB (2800)
rs12060189	41160281	BB (2385)	BB (3236)	BB (3236)	BB (3236)	BB (3290)	BB (2800)
rs7551747	41165079	AA (2385)	AA (3236)	AA (3236)	AA (3236)	AA (3290)	AA (2800)
rs10489167	41176466	BB (2385)	BB (3236)	BB (3236)	BB (3236)	BB (3290)	BB (2800)
rs2744808	41177943	BB (2385)	BB (3236)	BB (3236)	BB (3236)	BB (3290)	BB (2800)
rs2744803	41183105	AA (2385)	AA (3236)	AA (3236)	AA (3236)	AA (3290)	AA (2800)
rs944940	41205276	BB (2385)	BB (3236)	BB (3236)	BB (3236)	BB (3290)	BB (2800)
rs4660449	41205452	AA (2385)	AA (3236)	AA (3236)	AA (3236)	AA (3290)	AA (2800)
rs16827546	41222889	AA (2385)	AA (3236)	AA (3236)	AA (3236)	AA (3290)	AA (2800)
rs10158384	41225520	AA (2385)	AA (3236)	AA (3236)	AA (3236)	AA (3290)	AA (2800)
rs11208830	41226602	AA (2385)	AA (3236)	AA (3236)	AA (3236)	AA (3290)	AA (2800)
rs3754176	41235946	BB (2385)	BB (3236)	BB (3236)	BB (3236)	BB (3290)	BB (2800)
rs2251413	41245859	AA (2385)	AA (3236)	AA (3236)	AA (3236)	AA (3290)	AA (2800)
rs823671	41255120	AA (2385)	AA (3236)	AA (3236)	AA (3236)	AA (3290)	AA (2800)
rs12408769	41270163	AA (2385)	AA (3236)	AA (3236)	AA (3236)	AA (3290)	AA (2800)
rs4660175	41274422	BB (2385)	BB (3236)	BB (3236)	BB (3236)	BB (3290)	BB (2800)
rs6661888	41281595	AA (2385)	AA (3236)	AA (3236)	AA (3236)	AA (3290)	AA (2800)
rs4660469	41286353	AA (2385)	AA (3236)	AA (3236)	AA (3236)	AA (3290)	AA (2800)
rs4483396	41287448	BB (2385)	BB (3236)	BB (3236)	BB (3236)	BB (3290)	BB (2800)
rs3767942	41291531	AA (2385)	AA (3236)	AA (3236)	AA (3236)	AA (3290)	AA (2800)
rs3767944	41293489	BB (2385)	BB (3236)	BB (3236)	BB (3236)	BB (3290)	BB (2800)
rs11801911	41293857	AA (2385)	AA (3236)	AA (3236)	AA (3236)	AA (3290)	AA (2800)
rs913380	41314358	AA (2385)	AA (3236)	AA (3236)	AA (3236)	AA (3290)	AA (2800)
rs11209020	41317020	BB (2385)	BB (3236)	BB (3236)	BB (3236)	BB (3290)	BB (2800)
rs12564411	41319052	AA (2385)	AA (3236)	AA (3236)	AA (3236)	AA (3290)	AA (2800)
rs11209075	41343061	BB (2385)	BB (3236)	BB (3236)	BB (3236)	BB (3290)	BB (2800)
rs35809276	41346428	AA (2385)	AA (3236)	AA (3236)	AA (3236)	AA (3290)	AA (2800)
rs11209104	41349252	AA (2385)	AA (3236)	AA (3236)	AA (3236)	AA (3290)	AA (2800)
rs12751530	41356780	AA (2385)	AA (3236)	AA (3236)	AA (3236)	AA (3290)	AA (2800)

rs6684543	41371339	BB (2385)	BB (3236)	BB (3236)	BB (3236)	BB (3290)	BB (2800)
rs11209145	41372237	BB (2385)	BB (3236)	BB (3236)	BB (3236)	BB (3290)	BB (2800)
rs3856250	41374443	AA (2385)	AA (3236)	AA (3236)	AA (3236)	AA (3290)	AA (2800)
rs16827770	41383724	BB (2385)	BB (3236)	BB (3236)	BB (3236)	BB (3290)	BB (2800)
rs6698251	41383838	BB (2385)	BB (3236)	BB (3236)	BB (3236)	BB (3290)	BB (2800)
rs7515340	41386871	AA (2385)	AA (3236)	AA (3236)	AA (3236)	AA (3290)	AA (2800)
rs7552587	41386888	AA (2385)	AA (3236)	AA (3236)	AA (3236)	AA (3290)	AA (2800)
rs17357836	41389957	AA (2385)	AA (3236)	AA (3236)	AA (3236)	AA (3290)	AA (2800)
rs12569014	41391350	AA (2385)	AA (3236)	AA (3236)	AA (3236)	AA (3290)	AA (2800)
rs1887542	41395072	BB (2385)	BB (3236)	BB (3236)	BB (3236)	BB (3290)	BB (2800)
rs6600358	41397716	AA (2385)	AA (3236)	AA (3236)	AA (3236)	AA (3290)	AA (2800)
rs12403975	41421510	AA (2385)	AA (3236)	AA (3236)	AA (3236)	AA (3290)	AA (2800)
rs12066993	41427973	BB (2385)	BB (3236)	BB (3236)	BB (3236)	BB (3290)	BB (2800)
rs7533657	41433484	BB (2385)	BB (3236)	BB (3236)	BB (3236)	BB (3290)	BB (2800)
rs10789262	41437417	AA (2385)	AA (3236)	AA (3236)	AA (3236)	AA (3290)	AA (2800)
rs7546898	41447165	BB (2385)	BB (3236)	BB (3236)	BB (3236)	BB (3290)	BB (2800)
rs10889792	41490885	AA (2385)	AA (3236)	AA (3236)	AA (3236)	AA (3290)	AA (2800)
rs7531344	41507431	BB (2385)	BB (3236)	BB (3236)	BB (3236)	BB (3290)	BB (2800)
rs9439033	41526023	AA (2385)	AA (3236)	AA (3236)	AA (3236)	AA (3290)	AA (2800)
rs6686842	41530871	BB (2385)	BB (3236)	BB (3236)	BB (3236)	BB (3290)	BB (2800)
rs12142006	41537825	BB (2385)	BB (3236)	BB (3236)	BB (3236)	BB (3290)	BB (2800)
rs2300650	41538787	AA (2385)	AA (3236)	AA (3236)	AA (3236)	AA (3290)	AA (2800)
rs6696524	41539494	BB (2385)	BB (3236)	BB (3236)	BB (3236)	BB (3290)	BB (2800)
rs2268677	41542504	BB (2385)	BB (3236)	BB (3236)	BB (3236)	BB (3290)	BB (2800)
rs3819842	41563086	BB (2385)	BB (3236)	BB (3236)	BB (3236)	BB (3290)	BB (2800)
rs721651	41563329	BB (2385)	BB (3236)	BB (3236)	BB (3236)	BB (3290)	BB (2800)
rs3766321	41565854	BB (2385)	BB (3236)	BB (3236)	BB (3236)	BB (3290)	BB (2800)
rs3766322	41565928	BB (2385)	BB (3236)	BB (3236)	BB (3236)	BB (3290)	BB (2800)
rs3766323	41566096	BB (2385)	BB (3236)	BB (3236)	BB (3236)	BB (3290)	BB (2800)
rs2300648	41569488	AA (2385)	AA (3236)	AA (3236)	AA (3236)	AA (3290)	AA (2800)
rs11209531	41571021	AA (2385)	AA (3236)	AA (3236)	AA (3236)	AA (3290)	AA (2800)
rs2363098	41573096	AA (2385)	AA (3236)	AA (3236)	AA (3236)	AA (3290)	AA (2800)
rs213751	41609886	BB (2385)	BB (3236)	BB (3236)	BB (3236)	BB (3290)	BB (2800)
rs213744	41619894	AA (2385)	AA (3236)	AA (3236)	AA (3236)	AA (3290)	AA (2800)
rs16827938	41620316	AA (2385)	AA (3236)	AA (3236)	AA (3236)	AA (3290)	AA (2800)
rs16827976	41657655	AA (2385)	AA (3236)	AA (3236)	AA (3236)	AB (3290)	AA (2800)
rs6672508	41666571	AA (2385)	AA (3236)	AA (3236)	AA (3236)	AA (3290)	AA (2800)
rs6672914	41666929	BB (2385)	BB (3236)	BB (3236)	BB (3236)	BB (3290)	BB (2800)
rs12121121	41684456	BB (2385)	BB (3236)	BB (3236)	BB (3236)	BB (3290)	BB (2800)
rs8179465	41701747	AA (2385)	AA (3236)	AA (3236)	AA (3236)	AA (3290)	AA (2800)
rs10493093	41702186	BB (2385)	BB (3236)	BB (3236)	BB (3236)	BB (3290)	BB (2800)
rs12123384	41705018	BB (2385)	BB (3236)	BB (3236)	BB (3236)	BB (3290)	BB (2800)
rs1472570	41714084	AA (2385)	AA (3236)	AA (3236)	AA (3236)	AA (3290)	AA (2800)
rs10157966	41722576	AA (2385)	AA (3236)	AA (3236)	AA (3236)	AA (3290)	AA (2800)
rs12035913	41757057	BB (2385)	BB (3236)	BB (3236)	BB (3236)	BB (3290)	BB (2800)
rs2364535	41766236	AA (2385)	AA (3236)	AA (3236)	AA (3236)	AA (3290)	AA (2800)
rs12042464	41786854	BB (2385)	BB (3236)	BB (3236)	BB (3236)	BB (3290)	BB (2800)
rs7528407	41787999	AA (2385)	AA (3236)	AA (3236)	AA (3236)	AA (3290)	AA (2800)
rs12043581	41788265	AA (2385)	AA (3236)	AA (3236)	AA (3236)	AA (3290)	AA (2800)
rs12037129	41794301	AA (2385)	AA (3236)	AA (3236)	AA (3236)	AA (3290)	AA (2800)

rs17362424	41794403		BB (2385)	BB (3236)	BB (3236)	BB (3236)	BB (3290)	BB (2800)
rs16828117	41794518		BB (2385)	BB (3236)	BB (3236)	BB (3236)	BB (3290)	BB (2800)
rs16828119	41797098		BB (2385)	BB (3236)	BB (3236)	BB (3236)	BB (3290)	BB (2800)
rs7355092	41800596		BB (2385)	BB (3236)	BB (3236)	BB (3236)	BB (3290)	BB (2800)
rs4660522	41804234		BB (2385)	BB (3236)	BB (3236)	BB (3236)	BB (3290)	BB (2800)
rs7531976	41808945		BB (2385)	BB (3236)	BB (3236)	BB (3236)	BB (3290)	BB (2800)
rs12757611	41808972		AA (2385)	AA (3236)	AA (3236)	AA (3236)	AA (3290)	AA (2800)
rs12047439	41809640		AA (2385)	AA (3236)	AA (3236)	AA (3236)	AA (3290)	AA (2800)
rs4609415	41812194		AA (2385)	AA (3236)	AA (3236)	AA (3236)	AA (3290)	AA (2800)
rs6660265	41818676		BB (2385)	BB (3236)	BB (3236)	BB (3236)	BB (3290)	BB (2800)
rs4660527	41821779		BB (2385)	BB (3236)	BB (3236)	BB (3236)	BB (3290)	BB (2800)
rs7349069	41822753		AA (2385)	AA (3236)	AA (3236)	AA (3236)	AA (3290)	AA (2800)
rs1317557	41839342		BB (2385)	BB (3236)	BB (3236)	BB (3236)	BB (3290)	BB (2800)
rs11209971	41844293		BB (2385)	BB (3236)	BB (3236)	BB (3236)	BB (3290)	BB (2800)
rs10749805	41850182		BB (2385)	BB (3236)	BB (3236)	BB (3236)	BB (3290)	BB (2800)
rs2886109	41854217		BB (2385)	BB (3236)	BB (3236)	BB (3236)	BB (3290)	BB (2800)
rs4660533	41854557		AA (2385)	AA (3236)	AA (3236)	AA (3236)	AA (3290)	AA (2800)
rs2226263	41854946		BB (2385)	BB (3236)	BB (3236)	BB (3236)	BB (3290)	BB (2800)
rs10889961	41855174		BB (2385)	BB (3236)	BB (3236)	BB (3236)	BB (3290)	BB (2800)
rs12041455	41856548		AA (2385)	AA (3236)	AA (3236)	AA (3236)	AA (3290)	AA (2800)
rs7538090	41858283		AA (2385)	AA (3236)	AA (3236)	AA (3236)	AA (3290)	AA (2800)
rs10889978	41862125		AA (2385)	AA (3236)	AA (3236)	AA (3236)	AA (3290)	AA (2800)
rs11210026	41864971		BB (2385)	BB (3236)	BB (3236)	BB (3236)	BB (3290)	BB (2800)
rs2810569	41895497		BB (2385)	BB (3236)	BB (3236)	BB (3236)	BB (3290)	BB (2800)
rs12086029	41903038		AA (2385)	AA (3236)	AA (3236)	AA (3236)	AA (3290)	AA (2800)
rs4262533	41904847		AA (2385)	AA (3236)	AA (3236)	AA (3236)	AA (3290)	AA (2800)
rs12408331	41913567		BB (2385)	BB (3236)	BB (3236)	BB (3236)	BB (3290)	BB (2800)
rs17358725	41915883		AA (2385)	AA (3236)	AA (3236)	AA (3236)	AA (3290)	AA (2800)
rs2483692	41925050		BB (2385)	BB (3236)	BB (3236)	BB (3236)	BB (3290)	BB (2800)
rs11590973	41926652		AA (2385)	AA (3236)	AA (3236)	AA (3236)	AA (3290)	AA (2800)
rs2781248	41929252		BB (2385)	BB (3236)	BB (3236)	BB (3236)	BB (3290)	BB (2800)
rs2810577	41930476		BB (2385)	BB (3236)	BB (3236)	BB (3236)	BB (3290)	BB (2800)
rs2759257	41949628		AA (2385)	AA (3236)	AA (3236)	AA (3236)	AA (3290)	AA (2800)
rs2991345	41969327		AA (2385)	AA (3236)	AA (3236)	AA (3236)	AA (3290)	AA (2800)
rs11210344	41972880		BB (2385)	BB (3236)	BB (3236)	BB (3236)	BB (3290)	BB (2800)
rs3738577	41973349		AA (2385)	AA (3236)	AA (3236)	AA (3236)	AA (3290)	AA (2800)
rs2151674	41974258		BB (2385)	BB (3236)	BB (3236)	BB (3236)	BB (3290)	BB (2800)
rs2810547	41983912		BB (2385)	BB (3236)	BB (3236)	BB (3236)	BB (3290)	BB (2800)
rs633297	41985453		AA (2385)	AA (3236)	AA (3236)	AA (3236)	AA (3290)	AA (2800)
rs646228	41986066		AA (2385)	AA (3236)	AA (3236)	AA (3236)	AA (3290)	AA (2800)
rs679449	41988634		BB (2385)	BB (3236)	BB (3236)	BB (3236)	BB (3290)	BB (2800)
rs662999	42001160		AA (2385)	AA (3236)	AA (3236)	AA (3236)	AA (3290)	AA (2800)
rs10890105	42001301		AA (2385)	AA (3236)	AA (3236)	AA (3236)	AA (3290)	AA (2800)
rs638859	42001444		AA (2385)	AA (3236)	AA (3236)	AA (3236)	AA (3290)	AA (2800)
rs12121881	42002738		AA (2385)	AA (3236)	AA (3236)	AA (3236)	AA (3290)	AA (2800)
rs6664458	42005041		BB (2385)	BB (3236)	BB (3236)	BB (3236)	BB (3290)	BB (2800)
rs2475839	42006005		BB (2385)	BB (3236)	BB (3236)	BB (3236)	BB (3290)	BB (2800)
rs10493102	42008521		BB (2385)	BB (3236)	BB (3236)	BB (3236)	BB (3290)	BB (2800)
rs636850	42009004		BB (2385)	BB (3236)	BB (3236)	BB (3236)	BB (3290)	BB (2800)
rs10789394	42026023		AA (2385)	AA (3236)	AA (3236)	AA (3236)	AA (3290)	AA (2800)

rs4509569	42037875		AA (2385)	AA (3236)	AA (3236)	AA (3236)	AA (3290)	AA (2800)
rs2759251	42041749		BB (2385)	BB (3236)	BB (3236)	BB (3236)	BB (3290)	BB (2800)
rs10749832	42041806		AA (2385)	AA (3236)	AA (3236)	AA (3236)	AA (3290)	AA (2800)
rs1570355	42042317		AA (2385)	AA (3236)	AA (3236)	AA (3236)	AA (3290)	AA (2800)
rs6600380	42054814		BB (2385)	BB (3236)	BB (3236)	BB (3236)	BB (3290)	BB (2800)
rs6679348	42057729		AA (2385)	AA (3236)	AA (3236)	AA (3236)	AA (3290)	AA (2800)
rs1073197	42059822		BB (2385)	BB (3236)	BB (3236)	BB (3236)	BB (3290)	BB (2800)
rs12731266	42062084		AA (2385)	AA (3236)	AA (3236)	AA (3236)	AA (3290)	AA (2800)
rs7515066	42076915		BB (2385)	BB (3236)	BB (3236)	BB (3236)	BB (3290)	BB (2800)
rs4083497	42079867		BB (2385)	BB (3236)	BB (3236)	BB (3236)	BB (3290)	BB (2800)
rs7519983	42083369		AA (2385)	AA (3236)	AA (3236)	AA (3236)	AA (3290)	AA (2800)
rs16828500	42085778		BB (2385)	BB (3236)	BB (3236)	BB (3236)	BB (3290)	BB (2800)
rs4660549	42088102		AA (2385)	AA (3236)	AA (3236)	AA (3236)	AA (3290)	AA (2800)
rs6658500	42088344		BB (2385)	BB (3236)	BB (3236)	BB (3236)	BB (3290)	BB (2800)
rs10890147	42089725		AA (2385)	AA (3236)	AA (3236)	AA (3236)	AA (3290)	AA (2800)
rs11210502	42093712		AA (2385)	AA (3236)	AA (3236)	AA (3236)	AA (3290)	AA (2800)
rs2038977	42094659		AA (2385)	AA (3236)	AA (3236)	AA (3236)	AA (3290)	AA (2800)
rs11807949	42102877		AA (2385)	AA (3236)	AA (3236)	AA (3236)	AA (3290)	AA (2800)
rs10789406	42105182		BB (2385)	BB (3236)	BB (3236)	BB (3236)	BB (3290)	BB (2800)
rs11210509	42109311		AA (2385)	AA (3236)	AA (3236)	AA (3236)	AA (3290)	AA (2800)
rs1123483	42114198		BB (2385)	BB (3236)	BB (3236)	BB (3236)	BB (3290)	BB (2800)
rs6701758	42117371		BB (2385)	BB (3236)	BB (3236)	BB (3236)	BB (3290)	BB (2800)
rs2148636	42119844		BB (2385)	BB (3236)	BB (3236)	BB (3236)	BB (3290)	BB (2800)
rs17372813	42139393		AA (2385)	AA (3236)	AA (3236)	AA (3236)	AA (3290)	AA (2800)
rs6700172	42142389		BB (2385)	BB (3236)	BB (3236)	BB (3236)	BB (3290)	BB (2800)
rs1105413	42144906		AA (2385)	AA (3236)	AA (3236)	AA (3236)	AA (3290)	AA (2800)
rs12066191	42165131		BB (2385)	BB (3236)	BB (3236)	BB (3236)	BB (3290)	BB (2800)
rs12086915	42174630		BB (2385)	BB (3236)	BB (3236)	BB (3236)	BB (3290)	BB (2800)
rs2036961	42178084		AA (2385)	AA (3236)	AA (3236)	AA (3236)	AA (3290)	AA (2800)
rs2077354	42180082		BB (2385)	BB (3236)	BB (3236)	BB (3236)	BB (3290)	BB (2800)
rs4526604	42182144		BB (2385)	BB (3236)	BB (3236)	BB (3236)	BB (3290)	BB (2800)
rs10493099	42187205		AA (2385)	AA (3236)	AA (3236)	AA (3236)	AA (3290)	AA (2800)
rs12088923	42192360		BB (2385)	BB (3236)	BB (3236)	BB (3236)	BB (3290)	BB (2800)
rs710235	42202714		AA (2385)	AA (3236)	AA (3236)	AA (3236)	AA (3290)	AA (2800)
rs710234	42202857		AA (2385)	AA (3236)	AA (3236)	AA (3236)	AA (3290)	AA (2800)
rs2668939	42206473		AA (2385)	AA (3236)	AA (3236)	AA (3236)	AA (3290)	AA (2800)
rs16828605	42207719		AA (2385)	AA (3236)	AA (3236)	AA (3236)	AA (3290)	AA (2800)
rs782657	42208516		AA (2385)	AA (3236)	AA (3236)	AA (3236)	AA (3290)	AA (2800)
rs4284254	42213076		AA (2385)	AA (3236)	AA (3236)	AA (3236)	AA (3290)	AA (2800)
rs12126740	42223909		AA (2385)	AA (3236)	AA (3236)	AA (3236)	AA (3290)	AA (2800)
rs4660204	42226802		BB (2385)	BB (3236)	BB (3236)	BB (3236)	BB (3290)	BB (2800)
rs2165303	42228019		AA (2385)	AA (3236)	AA (3236)	AA (3236)	AA (3290)	AA (2800)
rs871343	42230685		BB (2385)	BB (3236)	BB (3236)	BB (3236)	BB (3290)	BB (2800)
rs7535437	42236828		AA (2385)	AA (3236)	AA (3236)	AA (3236)	AA (3290)	AA (2800)
rs402379	42247715		BB (2385)	BB (3236)	BB (3236)	BB (3236)	-3290	BB (2800)
rs7517484	42250456		BB (2385)	BB (3236)	BB (3236)	BB (3236)	BB (3290)	BB (2800)
rs239340	42252358		BB (2385)	BB (3236)	BB (3236)	BB (3236)	BB (3290)	BB (2800)
rs349433	42255087		BB (2385)	BB (3236)	BB (3236)	BB (3236)	BB (3290)	BB (2800)
rs239341	42255221		AA (2385)	AA (3236)	AA (3236)	AA (3236)	AA (3290)	AA (2800)
rs349439	42259008		AA (2385)	AA (3236)	AA (3236)	AA (3236)	AA (3290)	AA (2800)

rs17373758	42259133		AA (2385)	AA (3236)	AA (3236)	AA (3236)	AA (3290)	AA (2800)
rs349441	42260686		BB (2385)	BB (3236)	BB (3236)	BB (3236)	-3290	BB (2800)
rs10493097	42262138		AA (2385)	AA (3236)	AA (3236)	AA (3236)	AA (3290)	AA (2800)
rs16828715	42268979		AA (2385)	AA (3236)	AA (3236)	AA (3236)	AA (3290)	AA (2800)
rs349454	42269458		AA (2385)	AA (3236)	AA (3236)	AA (3236)	AA (3290)	AA (2800)
rs16828722	42270666		AA (2385)	AA (3236)	AA (3236)	AA (3236)	AA (3290)	AA (2800)
rs349447	42272924		AA (2385)	AA (3236)	AA (3236)	AA (3236)	AA (3290)	AA (2800)
rs12563071	42273653		BB (2385)	BB (3236)	BB (3236)	BB (3236)	BB (3290)	BB (2800)
rs2492603	42285999		AA (2385)	AA (3236)	AA (3236)	AA (3236)	AA (3290)	AA (2800)
rs972352	42297604		AA (2385)	AA (3236)	AA (3236)	AA (3236)	AA (3290)	AA (2800)
rs41434744	42299640		AA (2385)	AA (3236)	AA (3236)	AA (3236)	AA (3290)	AA (2800)
rs2786484	42299909		AA (2385)	AA (3236)	AA (3236)	AA (3236)	AA (3290)	AA (2800)
rs681511	42301301		AA (2385)	AA (3236)	AA (3236)	AA (3236)	AA (3290)	AA (2800)
rs16828739	42301595		AA (2385)	AA (3236)	AA (3236)	AA (3236)	AA (3290)	AA (2800)
rs509851	42307194		AA (2385)	AA (3236)	AA (3236)	AA (3236)	AA (3290)	AA (2800)
rs661225	42310165		AA (2385)	AA (3236)	AA (3236)	AA (3236)	AA (3290)	AA (2800)
rs661662	42310282		AA (2385)	AA (3236)	AA (3236)	AA (3236)	AA (3290)	AA (2800)
rs1866758	42318868		AA (2385)	AA (3236)	AA (3236)	AA (3236)	AA (3290)	AA (2800)
rs783313	42321295		BB (2385)	BB (3236)	BB (3236)	BB (3236)	BB (3290)	BB (2800)
rs997385	42322052		BB (2385)	BB (3236)	BB (3236)	BB (3236)	BB (3290)	BB (2800)
rs10493096	42325122		BB (2385)	BB (3236)	BB (3236)	BB (3236)	BB (3290)	BB (2800)
rs525382	42329120		BB (2385)	BB (3236)	BB (3236)	BB (3236)	BB (3290)	BB (2800)
rs609572	42329303		AA (2385)	AA (3236)	AA (3236)	AA (3236)	AA (3290)	AA (2800)
rs7535166	42331630		BB (2385)	BB (3236)	BB (3236)	-3236	-3290	BB (2800)
rs783424	42338724		BB (2385)	BB (3236)	BB (3236)	BB (3236)	BB (3290)	BB (2800)
rs2993120	42350837		BB (2385)	BB (3236)	BB (3236)	BB (3236)	BB (3290)	BB (2800)
rs809173	42363381		BB (2385)	BB (3236)	BB (3236)	BB (3236)	BB (3290)	BB (2800)
rs783622	42366988		AA (2385)	AA (3236)	AA (3236)	AA (3236)	AA (3290)	AA (2800)
rs2786485	42378784		BB (2385)	BB (3236)	BB (3236)	BB (3236)	BB (3290)	BB (2800)
rs41513349	42399966		BB (2385)	BB (3236)	BB (3236)	BB (3236)	BB (3290)	BB (2800)
rs1408948	42417706		BB (2385)	BB (3236)	BB (3236)	BB (3236)	BB (3290)	BB (2800)
rs660883	42422757		AA (2385)	AA (3236)	AA (3236)	AA (3236)	AA (3290)	AA (2800)
rs11210556	42423684		AA (2385)	AA (3236)	AA (3236)	AA (3236)	AA (3290)	AA (2800)
rs591088	42438196		BB (2385)	BB (3236)	BB (3236)	BB (3236)	BB (3290)	BB (2800)
rs16828938	42439410		BB (2385)	BB (3236)	BB (3236)	BB (3236)	BB (3290)	BB (2800)
rs3845573	42440758		AA (2385)	AA (3236)	AA (3236)	AA (3236)	AA (3290)	AA (2800)
rs4660590	42441288		BB (2385)	BB (3236)	BB (3236)	BB (3236)	BB (3290)	BB (2800)
rs642112	42453317		AA (2385)	AA (3236)	AA (3236)	AB (3236)	AB (3290)	AA (2800)
rs189426	42465728		AA (2385)	AA (3236)	AA (3236)	AA (3236)	AA (3290)	AA (2800)
rs7553696	42470941		AA (2385)	AA (3236)	AA (3236)	AA (3236)	AA (3290)	AA (2800)
rs784103	42487059		AA (2385)	AA (3236)	AA (3236)	AA (3236)	AA (3290)	AA (2800)
rs12042133	42499365		BB (2385)	BB (3236)	BB (3236)	BB (3236)	BB (3290)	BB (2800)
rs6696291	42500421		BB (2385)	BB (3236)	BB (3236)	BB (3236)	BB (3290)	BB (2800)
rs7550497	42505510		BB (2385)	BB (3236)	BB (3236)	BB (3236)	BB (3290)	BB (2800)
rs12143100	42508951		AA (2385)	AA (3236)	AA (3236)	AA (3236)	AA (3290)	AA (2800)
rs953194	42517167		BB (2385)	BB (3236)	BB (3236)	BB (3236)	BB (3290)	BB (2800)
rs7545779	42568348		AA (2385)	AA (3236)	AA (3236)	AA (3236)	AA (3290)	AA (2800)
rs1016867	42570803		BB (2385)	BB (3236)	BB (3236)	BB (3236)	BB (3290)	BB (2800)
rs11210588	42580503		AA (2385)	AA (3236)	AA (3236)	AA (3236)	AA (3290)	AA (2800)
rs10789415	42591328		BB (2385)	BB (3236)	BB (3236)	BB (3236)	-3290	BB (2800)

rs17367664	42593668		AA (2385)	AA (3236)	AA (3236)	AA (3236)	AA (3290)	AA (2800)
rs6600401	42607625		BB (2385)	BB (3236)	BB (3236)	BB (3236)	AB (3290)	BB (2800)
rs11210604	42623672		AA (2385)	AA (3236)	AA (3236)	AA (3236)	AA (3290)	AA (2800)
rs6665972	42673523		AA (2385)	AA (3236)	AA (3236)	AA (3236)	AA (3290)	AA (2800)
rs693587	42683284		AA (2385)	AA (3236)	AA (3236)	AA (3236)	AA (3290)	AA (2800)
rs592348	42685235		AA (2385)	AA (3236)	AA (3236)	AA (3236)	AA (3290)	AA (2800)
rs624635	42688966		BB (2385)	BB (3236)	BB (3236)	BB (3236)	BB (3290)	BB (2800)
rs343377	42695198		BB (2385)	BB (3236)	BB (3236)	BB (3236)	BB (3290)	BB (2800)
rs343373	42705047		AA (2385)	AA (3236)	AA (3236)	AA (3236)	AA (3290)	AA (2800)
rs343388	42707630		BB (2385)	BB (3236)	BB (3236)	BB (3236)	BB (3290)	BB (2800)
rs7526659	42745975		BB (2385)	BB (3236)	BB (3236)	BB (3236)	BB (3290)	BB (2800)
rs11210617	42764188		BB (2385)	BB (3236)	BB (3236)	BB (3236)	BB (3290)	BB (2800)
rs11210621	42779088		BB (2385)	BB (3236)	BB (3236)	BB (3236)	BB (3290)	BB (2800)
rs2479462	42780261		BB (2385)	BB (3236)	BB (3236)	BB (3236)	BB (3290)	BB (2800)
rs1566584	42797765		BB (2385)	BB (3236)	BB (3236)	BB (3236)	BB (3290)	BB (2800)
rs2494356	42823366		BB (2385)	BB (3236)	BB (3236)	BB (3236)	BB (3290)	BB (2800)
rs1536651	42823768		BB (2385)	BB (3236)	BB (3236)	BB (3236)	BB (3290)	BB (2800)
rs17377828	42824317		BB (2385)	BB (3236)	BB (3236)	BB (3236)	BB (3290)	BB (2800)
rs12408746	42856729		AA (2385)	AA (3236)	AA (3236)	AA (3236)	AA (3290)	AA (2800)
rs17369630	42860217		AA (2385)	AA (3236)	AA (3236)	AA (3236)	AA (3290)	AA (2800)
rs6684354	42861950		AA (2385)	AA (3236)	AA (3236)	AA (3236)	AA (3290)	AA (2800)
rs17369712	42862409		AA (2385)	AA (3236)	AA (3236)	AA (3236)	AA (3290)	AA (2800)
rs1055055	42880516		AA (2385)	AA (3236)	AA (3236)	AA (3236)	AA (3290)	AA (2800)
rs10493109	42886185		BB (2385)	BB (3236)	BB (3236)	BB (3236)	BB (3290)	BB (2800)
rs1110071	42889224		BB (2385)	BB (3236)	BB (3236)	BB (3236)	BB (3290)	BB (2800)
rs11210643	42893164		BB (2385)	BB (3236)	BB (3236)	BB (3236)	BB (3290)	BB (2800)
rs1011810	42898633		AA (2385)	AA (3236)	AA (3236)	AA (3236)	AA (3290)	AA (2800)
rs2494376	42898671		AA (2385)	AA (3236)	AA (3236)	AA (3236)	AA (3290)	AA (2800)
rs1034268	42898843		BB (2385)	BB (3236)	BB (3236)	BB (3236)	BB (3290)	BB (2800)
rs3748845	42901082		AA (2385)	AA (3236)	AA (3236)	AA (3236)	AA (3290)	AA (2800)
rs4660634	42901173		BB (2385)	BB (3236)	BB (3236)	BB (3236)	BB (3290)	BB (2800)
rs10890197	42902906		AA (2385)	AA (3236)	AA (3236)	AA (3236)	AA (3290)	AA (2800)
rs2809664	42908015		BB (2385)	BB (3236)	BB (3236)	BB (3236)	BB (3290)	BB (2800)
rs2809665	42908046		BB (2385)	BB (3236)	BB (3236)	BB (3236)	BB (3290)	BB (2800)
rs10493108	42908102		BB (2385)	BB (3236)	BB (3236)	BB (3236)	BB (3290)	BB (2800)
rs10437062	42908219		AA (2385)	AA (3236)	AA (3236)	AA (3236)	AA (3290)	AA (2800)
rs12047502	42928730		BB (2385)	BB (3236)	BB (3236)	BB (3236)	BB (3290)	BB (2800)
rs11210651	42945714		AA (2385)	AA (3236)	AA (3236)	AA (3236)	AA (3290)	AA (2800)
rs1335578	42950208		BB (2385)	BB (3236)	BB (3236)	BB (3236)	BB (3290)	BB (2800)
rs12405571	42959296		BB (2385)	BB (3236)	BB (3236)	BB (3236)	BB (3290)	BB (2800)
rs6698350	42962221		AA (2385)	AA (3236)	AA (3236)	AA (3236)	AA (3290)	AA (2800)
rs4622104	42978242		AA (2385)	AA (3236)	AA (3236)	AA (3236)	AA (3290)	AA (2800)
rs4607934	42983413		AA (2385)	AA (3236)	AA (3236)	AA (3236)	AA (3290)	AA (2800)
rs10890200	42999787		BB (2385)	BB (3236)	BB (3236)	BB (3236)	BB (3290)	BB (2800)
rs7411957	43008018		AA (2385)	AA (3236)	AA (3236)	AA (3236)	AA (3290)	AA (2800)
rs16829623	43009319		BB (2385)	BB (3236)	-3236	BB (3236)	BB (3290)	BB (2800)
rs16829690	43020694		BB (2385)	BB (3236)	BB (3236)	BB (3236)	BB (3290)	BB (2800)
rs2809658	43050396		AA (2385)	AA (3236)	AA (3236)	AA (3236)	AA (3290)	AA (2800)
rs11210675	43050819		BB (2385)	BB (3236)	BB (3236)	BB (3236)	BB (3290)	BB (2800)
rs16829778	43051832		AA (2385)	AA (3236)	AA (3236)	AA (3236)	AA (3290)	AA (2800)

rs12408987	43052471		AA (2385)	AA (3236)	AA (3236)	AA (3236)	AA (3290)	AA (2800)
rs2762693	43052856		BB (2385)	BB (3236)	BB (3236)	BB (3236)	BB (3290)	BB (2800)
rs2762694	43053367		BB (2385)	BB (3236)	BB (3236)	BB (3236)	BB (3290)	BB (2800)
rs12024669	43073464		BB (2385)	BB (3236)	BB (3236)	BB (3236)	BB (3290)	BB (2800)
rs12025456	43073504		AA (2385)	AA (3236)	AA (3236)	AA (3236)	AA (3290)	AA (2800)
rs41432649	43111721		AA (2385)	AA (3236)	AA (3236)	AA (3236)	AA (3290)	AA (2800)
rs16829834	43120821		BB (2385)	BB (3236)	BB (3236)	BB (3236)	BB (3290)	BB (2800)
rs3768027	43129869		AA (2385)	AA (3236)	AA (3236)	AA (3236)	AA (3290)	AA (2800)
rs3862218	43152610		BB (2385)	BB (3236)	BB (3236)	BB (3236)	BB (3290)	BB (2800)
rs3895305	43154492		BB (2385)	BB (3236)	BB (3236)	BB (3236)	BB (3290)	BB (2800)
rs11210696	43155062		BB (2385)	BB (3236)	BB (3236)	BB (3236)	BB (3290)	BB (2800)
rs12044179	43163546		BB (2385)	BB (3236)	BB (3236)	BB (3236)	BB (3290)	BB (2800)
rs11210709	43209539		BB (2385)	AB (3236)	-3236	-3236	-3290	BB (2800)
rs16829937	43215178		AA (2385)	AA (3236)	AA (3236)	AA (3236)	AA (3290)	AA (2800)
rs2760081	43262673		AA (2385)	AA (3236)	AA (3236)	AA (3236)	AA (3290)	AA (2800)
rs323720	43271033		AA (2385)	AA (3236)	AA (3236)	AA (3236)	AA (3290)	AA (2800)
rs655741	43273753		BB (2385)	BB (3236)	BB (3236)	BB (3236)	BB (3290)	BB (2800)
rs323716	43277312		BB (2385)	BB (3236)	BB (3236)	BB (3236)	BB (3290)	BB (2800)
rs17381767	43281728		AA (2385)	AA (3236)	AA (3236)	AA (3236)	AA (3290)	AA (2800)
rs11210726	43299121		AA (2385)	AA (3236)	AA (3236)	AA (3236)	AA (3290)	AA (2800)
rs7540312	43299536		AA (2385)	AA (3236)	AA (3236)	AA (3236)	AA (3290)	AA (2800)
rs7540604	43299835		AA (2385)	AA (3236)	AA (3236)	AA (3236)	AA (3290)	AA (2800)
rs4660673	43300921		AA (2385)	AA (3236)	AA (3236)	AA (3236)	AA (3290)	AA (2800)
rs1484324	43309548		BB (2385)	BB (3236)	BB (3236)	BB (3236)	BB (3290)	BB (2800)
rs16830020	43316264		AA (2385)	AA (3236)	AA (3236)	AA (3236)	AA (3290)	AA (2800)
rs4660234	43319272		AA (2385)	AA (3236)	AA (3236)	AA (3236)	AA (3290)	AA (2800)
rs6674019	43353134		BB (2385)	BB (3236)	BB (3236)	BB (3236)	BB (3290)	BB (2800)
rs16830037	43357978		BB (2385)	BB (3236)	BB (3236)	BB (3236)	BB (3290)	BB (2800)
rs841860	43366891		AA (2385)	AA (3236)	AA (3236)	AA (3236)	AA (3290)	AA (2800)
rs12564800	43367726		AA (2385)	AA (3236)	AA (3236)	AA (3236)	AA (3290)	AA (2800)
rs900840	43369687		AA (2385)	AA (3236)	AA (3236)	AA (3236)	AA (3290)	AA (2800)
rs6600429	43373531		AA (2385)	AA (3236)	AA (3236)	AA (3236)	AA (3290)	AA (2800)
rs12565855	43373842		BB (2385)	BB (3236)	BB (3236)	BB (3236)	BB (3290)	BB (2800)
rs1601242	43373882		AA (2385)	AA (3236)	AA (3236)	AA (3236)	AA (3290)	AA (2800)
rs3820546	43398085		AA (2385)	AA (3236)	AA (3236)	AA (3236)	AA (3290)	AA (2800)
rs841858	43399167		AA (2385)	AA (3236)	AA (3236)	AA (3236)	AA (3290)	AA (2800)
rs3754219	43399686		BB (2385)	BB (3236)	BB (3236)	BB (3236)	BB (3290)	BB (2800)
rs5031048	43406154		AA (2385)	AA (3236)	AA (3236)	AA (3236)	AA (3290)	AA (2800)
rs12718444	43409179		AA (2385)	AA (3236)	AA (3236)	AA (3236)	AA (3290)	AA (2800)
rs17387886	43419705		BB (2385)	BB (3236)	BB (3236)	BB (3236)	BB (3290)	BB (2800)
rs12406072	43419737		AA (2385)	AA (3236)	AA (3236)	AA (3236)	AA (3290)	AA (2800)
rs3738514	43426591		BB (2385)	BB (3236)	BB (3236)	BB (3236)	BB (3290)	BB (2800)
rs710218	43427218		AA (2385)	AA (3236)	AA (3236)	AA (3236)	AA (3290)	AA (2800)
rs841839	43430084		AA (2385)	AA (3236)	AA (3236)	AA (3236)	AA (3290)	AA (2800)
rs2367159	43432589		BB (2385)	BB (3236)	BB (3236)	BB (3236)	BB (3290)	BB (2800)
rs11210774	43450729		BB (2385)	BB (3236)	BB (3236)	BB (3236)	BB (3290)	BB (2800)
rs1754154	43470766		BB (2385)	BB (3236)	BB (3236)	BB (3236)	BB (3290)	BB (2800)
rs9326132	43471182		AA (2385)	AA (3236)	AA (3236)	AA (3236)	AA (3290)	AA (2800)
rs597479	43486624		AA (2385)	AA (3236)	AA (3236)	AA (3236)	AA (3290)	AA (2800)
rs624969	43494779		AA (2385)	AA (3236)	AA (3236)	AA (3236)	AA (3290)	AA (2800)

rs17140015	43506477		AA (2385)	AA (3236)	AA (3236)	AA (3236)	AA (3290)	AA (2800)
rs4660242	43506623		AA (2385)	AA (3236)	AA (3236)	AA (3236)	AA (3290)	AA (2800)
rs666111	43507898		AA (2385)	AA (3236)	AA (3236)	AA (3236)	AA (3290)	AA (2800)
rs474731	43512213		BB (2385)	BB (3236)	BB (3236)	BB (3236)	BB (3290)	BB (2800)
rs17140030	43512400		BB (2385)	BB (3236)	BB (3236)	BB (3236)	BB (3290)	BB (2800)
rs17140042	43514626		BB (2385)	BB (3236)	BB (3236)	BB (3236)	BB (3290)	BB (2800)
rs16830248	43517953		AA (2385)	AA (3236)	AA (3236)	AA (3236)	AA (3290)	AA (2800)
rs588662	43530154		AA (2385)	AA (3236)	AA (3236)	AA (3236)	AA (3290)	AA (2800)
rs579001	43531531		BB (2385)	BB (3236)	BB (3236)	BB (3236)	BB (3290)	BB (2800)
rs1152771	43537981		BB (2385)	BB (3236)	BB (3236)	BB (3236)	BB (3290)	BB (2800)
rs583116	43548257		AA (2385)	AA (3236)	AA (3236)	AA (3236)	AA (3290)	AA (2800)
rs11210782	43548398		BB (2385)	BB (3236)	BB (3236)	BB (3236)	BB (3290)	BB (2800)
rs583682	43548419		AA (2385)	AA (3236)	AA (3236)	AA (3236)	AA (3290)	AA (2800)
rs16830359	43596384		BB (2385)	BB (3236)	BB (3236)	BB (3236)	BB (3290)	BB (2800)
rs694080	43596841		BB (2385)	BB (3236)	BB (3236)	BB (3236)	BB (3290)	BB (2800)
rs509668	43596933		AA (2385)	AA (3236)	AA (3236)	AA (3236)	AA (3290)	AA (2800)
rs585655	43597014		AA (2385)	AA (3236)	AA (3236)	AA (3236)	AA (3290)	AA (2800)
rs535099	43597392		AA (2385)	AA (3236)	AA (3236)	AA (3236)	AA (3290)	AA (2800)
rs558404	43602321		AA (2385)	AA (3236)	AA (3236)	AA (3236)	AA (3290)	AA (2800)
rs601504	43603715		BB (2385)	BB (3236)	BB (3236)	BB (3236)	BB (3290)	BB (2800)
rs16830399	43610139		AA (2385)	AA (3236)	AA (3236)	AA (3236)	AA (3290)	AA (2800)
rs627263	43611005		AA (2385)	AA (3236)	AA (3236)	AA (3236)	AA (3290)	AA (2800)
rs475093	43616337		AA (2385)	AA (3236)	AA (3236)	AA (3236)	AA (3290)	AA (2800)
rs16830421	43616646		AA (2385)	AA (3236)	AA (3236)	AA (3236)	AA (3290)	AA (2800)
rs610200	43616753		AA (2385)	AA (3236)	AA (3236)	AA (3236)	AA (3290)	AA (2800)
rs12033506	43618969		BB (2385)	BB (3236)	BB (3236)	BB (3236)	BB (3290)	BB (2800)
rs1612595	43620712		BB (2385)	BB (3236)	BB (3236)	BB (3236)	BB (3290)	BB (2800)
rs11210796	43627976		BB (2385)	BB (3236)	BB (3236)	BB (3236)	BB (3290)	BB (2800)
rs1198836	43628449		BB (2385)	BB (3236)	BB (3236)	BB (3236)	BB (3290)	BB (2800)
rs11210802	43640544		AA (2385)	AA (3236)	AA (3236)	AA (3236)	AA (3290)	AA (2800)
rs11210803	43640609		BB (2385)	BB (3236)	BB (3236)	BB (3236)	BB (3290)	BB (2800)
rs2453410	43640647		AA (2385)	AA (3236)	AA (3236)	AA (3236)	AA (3290)	AA (2800)
rs12087752	43641073		AA (2385)	AA (3236)	AA (3236)	AA (3236)	AA (3290)	AA (2800)
rs17390635	43642216		AA (2385)	AA (3236)	AA (3236)	AA (3236)	AA (3290)	AA (2800)
rs2764420	43673645		AA (2385)	AA (3236)	AA (3236)	AA (3236)	AA (3290)	AA (2800)
rs2453414	43674974		BB (2385)	BB (3236)	BB (3236)	BB (3236)	BB (3290)	BB (2800)
rs2453412	43675467		AA (2385)	AA (3236)	AA (3236)	AA (3236)	AA (3290)	AA (2800)
rs1760672	43678677		AA (2385)	AA (3236)	AA (3236)	AA (3236)	AA (3290)	AA (2800)
rs613956	43682461		BB (2385)	BB (3236)	BB (3236)	BB (3236)	BB (3290)	BB (2800)
rs644651	43683366		AA (2385)	AA (3236)	AA (3236)	AA (3236)	AA (3290)	AA (2800)
rs17391130	43683612		BB (2385)	BB (3236)	BB (3236)	BB (3236)	BB (3290)	BB (2800)
rs41509745	43699252		AA (2385)	AA (3236)	AA (3236)	AA (3236)	AA (3290)	AA (2800)
rs1198921	43699343		AA (2385)	AA (3236)	AA (3236)	AA (3236)	AA (3290)	AA (2800)
rs2484714	43699970		AA (2385)	AA (3236)	AA (3236)	AA (3236)	AA (3290)	AA (2800)
rs2483688	43700140		BB (2385)	BB (3236)	BB (3236)	BB (3236)	BB (3290)	BB (2800)
rs3862227	43720994		AA (2385)	AA (3236)	AA (3236)	AA (3236)	AA (3290)	AA (2800)
rs11210819	43721710		BB (2385)	BB (3236)	BB (3236)	BB (3236)	BB (3290)	BB (2800)
rs1760673	43730859		BB (2385)	BB (3236)	BB (3236)	BB (3236)	BB (3290)	BB (2800)
rs12731885	43745829		BB (2385)	BB (3236)	BB (3236)	BB (3236)	BB (3290)	BB (2800)
rs16830668	43745868		AA (2385)	AA (3236)	AA (3236)	AA (3236)	AA (3290)	AA (2800)

rs34783763	43757762		AA (2385)	AA (3236)	AA (3236)	AA (3236)	AA (3290)	AA (2800)
rs3120047	43764084		BB (2385)	BB (3236)	BB (3236)	BB (3236)	BB (3290)	BB (2800)
rs3806402	43764681		BB (2385)	BB (3236)	BB (3236)	BB (3236)	BB (3290)	BB (2800)
rs1467809	43774437		AA (2385)	AA (3236)	AA (3236)	AA (3236)	AA (3290)	AA (2800)
rs1098182	43785768		BB (2385)	BB (3236)	BB (3236)	BB (3236)	BB (3290)	BB (2800)
rs12076751	43792807		AA (2385)	AA (3236)	AA (3236)	AA (3236)	AA (3290)	AA (2800)
rs1199038	43800001		AA (2385)	AA (3236)	AA (3236)	AA (3236)	AB (3290)	AA (2800)
rs11210838	43827190		BB (2385)	BB (3236)	BB (3236)	BB (3236)	BB (3290)	BB (2800)
rs4141740	43842827		AA (2385)	AA (3236)	AA (3236)	AA (3236)	AA (3290)	AA (2800)
rs4141741	43843000		AA (2385)	AA (3236)	AA (3236)	AA (3236)	AA (3290)	AA (2800)
rs2494997	43878712		AA (2385)	AA (3236)	AA (3236)	AA (3236)	AA (3290)	AA (2800)
rs1999674	43881941		BB (2385)	BB (3236)	BB (3236)	BB (3236)	BB (3290)	BB (2800)
rs2842180	43901916		AA (2385)	AA (3236)	AA (3236)	AA (3236)	-3290	AA (2800)
rs2255632	43909265		BB (2385)	BB (3236)	BB (3236)	BB (3236)	BB (3290)	BB (2800)
rs2251804	43917409		BB (2385)	BB (3236)	BB (3236)	BB (3236)	AB (3290)	-2800
rs2782651	43920686		BB (2385)	BB (3236)	BB (3236)	BB (3236)	BB (3290)	BB (2800)
rs1334973	43921384		AA (2385)	AA (3236)	AA (3236)	AA (3236)	AA (3290)	AA (2800)
rs6429605	43957491		BB (2385)	BB (3236)	BB (3236)	BB (3236)	BB (3290)	BB (2800)
rs6687571	43957515		AA (2385)	AA (3236)	AA (3236)	AA (3236)	AA (3290)	AA (2800)
rs2039528	43960530		AA (2385)	AA (3236)	AA (3236)	AA (3236)	AA (3290)	AA (2800)
rs10890251	43976266		AA (2385)	AA (3236)	AA (3236)	AA (3236)	AA (3290)	AA (2800)
rs16830895	43980619		BB (2385)	BB (3236)	BB (3236)	BB (3236)	BB (3290)	BB (2800)
rs2367617	43981961		BB (2385)	BB (3236)	BB (3236)	BB (3236)	BB (3290)	BB (2800)
rs7518769	43987059		AA (2385)	AA (3236)	AA (3236)	AA (3236)	AA (3290)	AA (2800)
rs41341645	43987516		AA (2385)	AA (3236)	AA (3236)	AA (3236)	AA (3290)	AA (2800)
rs11210864	43991832		BB (2385)	BB (3236)	BB (3236)	BB (3236)	BB (3290)	BB (2800)
rs2819331	44004377		AA (2385)	AA (3236)	AA (3236)	AA (3236)	AA (3290)	AA (2800)
rs2782641	44013355		BB (2385)	BB (3236)	BB (3236)	BB (3236)	-3290	BB (2800)
rs11210871	44029353		AA (2385)	AA (3236)	AA (3236)	AA (3236)	AA (3290)	AA (2800)
rs10890257	44038837		BB (2385)	BB (3236)	BB (3236)	BB (3236)	BB (3290)	BB (2800)
rs11210879	44053259		BB (2385)	BB (3236)	BB (3236)	BB (3236)	BB (3290)	BB (2800)
rs3791148	44066730		BB (2385)	BB (3236)	BB (3236)	BB (3236)	BB (3290)	BB (2800)
rs11210886	44071546		BB (2385)	BB (3236)	BB (3236)	BB (3236)	BB (3290)	BB (2800)
rs664271	44075013		BB (2385)	BB (3236)	-3236	BB (3236)	BB (3290)	BB (2800)
rs641365	44083507		BB (2385)	BB (3236)	BB (3236)	BB (3236)	BB (3290)	BB (2800)
rs643445	44089632		AA (2385)	AA (3236)	AA (3236)	AA (3236)	AA (3290)	AA (2800)
rs10789438	44092027		AA (2385)	AA (3236)	AA (3236)	AA (3236)	AA (3290)	AA (2800)
rs11210892	44100084		AA (2385)	AA (3236)	AA (3236)	AA (3236)	AA (3290)	AA (2800)
rs617521	44114199		BB (2385)	BB (3236)	BB (3236)	BB (3236)	BB (3290)	BB (2800)
rs660899	44117006		BB (2385)	BB (3236)	BB (3236)	BB (3236)	BB (3290)	BB (2800)
rs2274465	44121557		AA (2385)	AA (3236)	AA (3236)	AA (3236)	AA (3290)	AA (2800)
rs10789439	44123191		BB (2385)	BB (3236)	BB (3236)	BB (3236)	BB (3290)	BB (2800)
rs489319	44131794		BB (2385)	BB (3236)	BB (3236)	BB (3236)	BB (3290)	BB (2800)
rs607062	44133016		BB (2385)	BB (3236)	BB (3236)	BB (3236)	BB (3290)	BB (2800)
rs10789442	44140075		AA (2385)	AA (3236)	AA (3236)	AA (3236)	AA (3290)	AA (2800)
rs3791035	44154479		BB (2385)	BB (3236)	BB (3236)	BB (3236)	BB (3290)	BB (2800)
rs304302	44170316		AA (2385)	AA (3236)	AA (3236)	AA (3236)	AA (3290)	AA (2800)
rs11210904	44180807		AA (2385)	AA (3236)	AA (3236)	AA (3236)	AA (3290)	AA (2800)
rs7520053	44226657		AA (2385)	AA (3236)	AA (3236)	AA (3236)	AA (3290)	AA (2800)
rs3791045	44230885		AA (2385)	AA (3236)	AA (3236)	AA (3236)	AA (3290)	AA (2800)

rs11590088	44233532		AA (2385)	AA (3236)	AA (3236)	AA (3236)	AA (3290)	AA (2800)
rs7549701	44245777		AA (2385)	AA (3236)	AA (3236)	AA (3236)	AA (3290)	AA (2800)
rs10890281	44245939		BB (2385)	BB (3236)	BB (3236)	BB (3236)	BB (3290)	BB (2800)
rs6691988	44254402		AA (2385)	AA (3236)	AA (3236)	AA (3236)	AA (3290)	AA (2800)
rs6701645	44254514		AA (2385)	AA (3236)	AA (3236)	AA (3236)	AA (3290)	AA (2800)
rs12401813	44263415		AA (2385)	AA (3236)	AA (3236)	AA (3236)	AA (3290)	AA (2800)
rs7515003	44264498		AA (2385)	AA (3236)	AA (3236)	AA (3236)	AA (3290)	AA (2800)
rs12024345	44269187		AA (2385)	AA (3236)	AA (3236)	AA (3236)	AA (3290)	AA (2800)
rs246777	44274434		BB (2385)	BB (3236)	BB (3236)	BB (3236)	BB (3290)	BB (2800)
rs3791062	44279223		AA (2385)	AA (3236)	AA (3236)	AA (3236)	AA (3290)	AA (2800)
rs37468	44280381		AA (2385)	AA (3236)	AA (3236)	AA (3236)	AA (3290)	AA (2800)
rs972444	44282204		AA (2385)	AA (3236)	AA (3236)	AA (3236)	AA (3290)	AA (2800)
rs3791068	44287116		BB (2385)	BB (3236)	BB (3236)	BB (3236)	BB (3290)	BB (2800)
rs3791073	44297673		BB (2385)	BB (3236)	BB (3236)	BB (3236)	BB (3290)	BB (2800)
rs42962	44298564		BB (2385)	BB (3236)	BB (3236)	BB (3236)	BB (3290)	BB (2800)
rs3011217	44303266		AA (2385)	AA (3236)	AA (3236)	AA (3236)	AA (3290)	AA (2800)
rs2906458	44336389		AA (2385)	AA (3236)	AA (3236)	AA (3236)	AA (3290)	AA (2800)
rs2906456	44340920		AA (2385)	AA (3236)	AA (3236)	AA (3236)	AA (3290)	AA (2800)
rs2428853	44343173		AA (2385)	AA (3236)	AA (3236)	AA (3236)	AA (3290)	AA (2800)
rs2788371	44343962		AA (2385)	AA (3236)	AA (3236)	AA (3236)	AA (3290)	AA (2800)
rs16831389	44363143		BB (2385)	BB (3236)	BB (3236)	BB (3236)	BB (3290)	BB (2800)
rs16831390	44363623		BB (2385)	BB (3236)	BB (3236)	BB (3236)	BB (3290)	BB (2800)
rs9326136	44390392		AA (2385)	AA (3236)	AA (3236)	AA (3236)	AA (3290)	AA (2800)
rs12569336	44391288		BB (2385)	BB (3236)	BB (3236)	BB (3236)	BB (3290)	BB (2800)
rs2108202	44395786		AA (2385)	AA (3236)	AA (3236)	AA (3236)	AA (3290)	AA (2800)
rs12561948	44396196		AA (2385)	AA (3236)	AA (3236)	AA (3236)	AA (3290)	AA (2800)
rs1859726	44396215		BB (2385)	BB (3236)	BB (3236)	BB (3236)	BB (3290)	BB (2800)
rs6801	44396646		AA (2385)	AA (3236)	AA (3236)	AA (3236)	AA (3290)	AA (2800)
rs6680421	44408969		AA (2385)	AA (3236)	AA (3236)	AA (3236)	AA (3290)	AA (2800)
rs1990150	44414127		BB (2385)	BB (3236)	BB (3236)	BB (3236)	BB (3290)	BB (2800)
rs1990151	44414292		BB (2385)	BB (3236)	BB (3236)	BB (3236)	BB (3290)	BB (2800)
rs6675620	44417630		BB (2385)	BB (3236)	BB (3236)	BB (3236)	BB (3290)	BB (2800)
rs2486013	44420248		AA (2385)	AA (3236)	AA (3236)	AA (3236)	AA (3290)	AA (2800)
rs1008137	44420754		BB (2385)	BB (3236)	BB (3236)	BB (3236)	BB (3290)	BB (2800)
rs7412307	44433864		AA (2385)	AA (3236)	AA (3236)	AA (3236)	AA (3290)	AA (2800)
rs7161	44438974		AA (2385)	AA (3236)	AA (3236)	AA (3236)	AA (3290)	AA (2800)
rs12410334	44442521		BB (2385)	BB (3236)	BB (3236)	BB (3236)	BB (3290)	BB (2800)
rs16831558	44502163		AA (2385)	AA (3236)	AA (3236)	AA (3236)	AA (3290)	AA (2800)
rs2485992	44502650		AA (2385)	AA (3236)	AA (3236)	AA (3236)	AA (3290)	AA (2800)
rs17413167	44507773		AA (2385)	AA (3236)	AA (3236)	AA (3236)	AA (3290)	AA (2800)
rs17413277	44510128		BB (2385)	BB (3236)	BB (3236)	BB (3236)	BB (3290)	BB (2800)
rs2057968	44515696		BB (2385)	BB (3236)	BB (3236)	BB (3236)	BB (3290)	BB (2800)
rs2158822	44519447		AA (2385)	AA (3236)	AA (3236)	AA (3236)	AA (3290)	AA (2800)
rs6429646	44523706		AA (2385)	AA (3236)	AA (3236)	AA (3236)	AA (3290)	AA (2800)
rs16831607	44534252		AA (2385)	AA (3236)	AA (3236)	AA (3236)	AA (3290)	AA (2800)
rs942755	44537401		BB (2385)	BB (3236)	BB (3236)	BB (3236)	BB (3290)	BB (2800)
rs7531328	44541316		BB (2385)	BB (3236)	BB (3236)	BB (3236)	BB (3290)	BB (2800)
rs6429648	44566221		AA (2385)	AA (3236)	AA (3236)	AA (3236)	AA (3290)	AA (2800)
rs509261	44568322		BB (2385)	BB (3236)	BB (3236)	BB (3236)	BB (3290)	BB (2800)
rs4660776	44568478		AA (2385)	AA (3236)	AA (3236)	AA (3236)	AA (3290)	AA (2800)

rs7527921	44570827	BB (2385)	BB (3236)	BB (3236)	BB (3236)	BB (3290)	BB (2800)
rs1408918	44571031	AA (2385)	AA (3236)	AA (3236)	AA (3236)	AA (3290)	AA (2800)
rs12048838	44571545	-2385	-3236	AB (3236)	-3236	AB (3290)	BB (2800)
rs663818	44578559	BB (2385)	BB (3236)	BB (3236)	BB (3236)	BB (3290)	BB (2800)
rs7513563	44585616	BB (2385)	BB (3236)	BB (3236)	BB (3236)	BB (3290)	BB (2800)
rs2485651	44603745	AA (2385)	AA (3236)	AA (3236)	AA (3236)	AA (3290)	AA (2800)
rs17414232	44623428	AA (2385)	AA (3236)	AA (3236)	AA (3236)	AA (3290)	AA (2800)
rs11210971	44635188	AA (2385)	AA (3236)	AA (3236)	AA (3236)	AA (3290)	AA (2800)
rs1291169	44681113	AA (2385)	AA (3236)	AA (3236)	AA (3236)	AA (3290)	AA (2800)
rs12419	44686322	BB (2385)	BB (3236)	BB (3236)	BB (3236)	BB (3290)	BB (2800)
rs1152030	44697673	AA (2385)	AA (3236)	AA (3236)	AA (3236)	AA (3290)	AA (2800)
rs17414912	44699848	BB (2385)	BB (3236)	BB (3236)	BB (3236)	BB (3290)	BB (2800)
rs325168	44731068	BB (2385)	BB (3236)	BB (3236)	BB (3236)	BB (3290)	BB (2800)
rs369493	44740024	BB (2385)	BB (3236)	BB (3236)	BB (3236)	BB (3290)	BB (2800)
rs10890295	44745450	AA (2385)	AA (3236)	AA (3236)	AA (3236)	AA (3290)	AA (2800)
rs325164	44751390	BB (2385)	BB (3236)	BB (3236)	BB (3236)	BB (3290)	BB (2800)
rs325163	44751710	AA (2385)	AA (3236)	AA (3236)	AA (3236)	AA (3290)	AA (2800)
rs325160	44759392	AA (2385)	AA (3236)	AA (3236)	AA (3236)	AA (3290)	AA (2800)
rs325155	44764547	AA (2385)	AA (3236)	AA (3236)	AA (3236)	AA (3290)	AA (2800)
rs6429535	44766071	AA (2385)	AA (3236)	AA (3236)	AA (3236)	AA (3290)	AA (2800)
rs325150	44767913	BB (2385)	BB (3236)	BB (3236)	BB (3236)	BB (3290)	BB (2800)
rs12026967	44776240	AA (2385)	AA (3236)	AA (3236)	AA (3236)	AA (3290)	AA (2800)
rs161725	44780322	BB (2385)	BB (3236)	BB (3236)	BB (3236)	BB (3290)	BB (2800)
rs161724	44781336	AA (2385)	AA (3236)	AA (3236)	AA (3236)	AA (3290)	AA (2800)
rs161721	44788398	AA (2385)	AA (3236)	AA (3236)	AA (3236)	AA (3290)	AA (2800)
rs161720	44790653	AA (2385)	AA (3236)	AA (3236)	AA (3236)	AA (3290)	AA (2800)
rs16831920	44795461	BB (2385)	BB (3236)	BB (3236)	BB (3236)	BB (3290)	BB (2800)
rs11210984	44804480	AA (2385)	AA (3236)	AA (3236)	AA (3236)	AA (3290)	AA (2800)
rs2524353	44807164	BB (2385)	BB (3236)	BB (3236)	BB (3236)	BB (3290)	BB (2800)
rs226067	44820319	AA (2385)	AA (3236)	AA (3236)	AA (3236)	AA (3290)	AA (2800)
rs6429539	44860224	AA (2385)	AA (3236)	AA (3236)	AA (3236)	AA (3290)	AA (2800)
rs11210992	44878549	BB (2385)	BB (3236)	BB (3236)	BB (3236)	BB (3290)	BB (2800)
rs1340647	44889509	AA (2385)	AA (3236)	AA (3236)	AA (3236)	AA (3290)	AA (2800)
rs12022308	44891488	BB (2385)	BB (3236)	BB (3236)	BB (3236)	BB (3290)	BB (2800)
rs6429540	44902285	AA (2385)	AA (3236)	AA (3236)	AA (3236)	AA (3290)	AA (2800)
rs7518522	44912073	AA (2385)	AA (3236)	AA (3236)	AA (3236)	AA (3290)	AA (2800)
rs7555873	44912147	AA (2385)	AA (3236)	AA (3236)	AA (3236)	AA (3290)	AA (2800)
rs1417371	44920992	BB (2385)	BB (3236)	BB (3236)	BB (3236)	BB (3290)	BB (2800)
rs4660278	44932745	AA (2385)	AA (3236)	AA (3236)	AA (3236)	AA (3290)	AA (2800)
rs7550326	44946187	BB (2385)	BB (3236)	BB (3236)	BB (3236)	BB (3290)	BB (2800)
rs7549585	44947616	BB (2385)	BB (3236)	BB (3236)	BB (3236)	BB (3290)	BB (2800)
rs12072574	44948098	AA (2385)	AA (3236)	AA (3236)	AA (3236)	AA (3290)	AA (2800)
rs2792597	44954342	BB (2385)	BB (3236)	BB (3236)	BB (3236)	BB (3290)	BB (2800)
rs6429541	44965723	AA (2385)	AA (3236)	AA (3236)	AA (3236)	AA (3290)	AA (2800)
rs272531	44988433	BB (2385)	BB (3236)	BB (3236)	BB (3236)	BB (3290)	BB (2800)
rs270724	44991278	AA (2385)	AA (3236)	AA (3236)	AA (3236)	AA (3290)	AA (2800)
rs272537	44992714	BB (2385)	BB (3236)	BB (3236)	BB (3236)	BB (3290)	BB (2800)
rs10890308	44998464	BB (2385)	BB (3236)	BB (3236)	BB (3236)	BB (3290)	BB (2800)
rs746733	44999614	AA (2385)	AA (3236)	AA (3236)	AA (3236)	AA (3290)	AA (2800)
rs7545984	45003893	AA (2385)	AA (3236)	AA (3236)	AA (3236)	AA (3290)	AA (2800)

rs7554123	45004204		AA (2385)	AA (3236)	AA (3236)	AA (3236)	AA (3290)	AA (2800)
rs3896232	45009801		AA (2385)	AA (3236)	AA (3236)	AA (3236)	AA (3290)	AA (2800)
rs41390645	45016280		BB (2385)	BB (3236)	BB (3236)	BB (3236)	BB (3290)	BB (2800)
rs156256	45018915		AA (2385)	AA (3236)	AA (3236)	AA (3236)	AA (3290)	AA (2800)
rs156255	45018937		BB (2385)	BB (3236)	BB (3236)	BB (3236)	BB (3290)	BB (2800)
rs2924869	45019168		BB (2385)	BB (3236)	BB (3236)	BB (3236)	BB (3290)	BB (2800)
rs12568345	45021601		BB (2385)	BB (3236)	BB (3236)	BB (3236)	BB (3290)	BB (2800)
rs69141	45039205		BB (2385)	BB (3236)	BB (3236)	BB (3236)	BB (3290)	BB (2800)
rs11810526	45040118		AA (2385)	AA (3236)	AA (3236)	AA (3236)	AA (3290)	AA (2800)
rs11211015	45042741		AA (2385)	AA (3236)	AA (3236)	AA (3236)	AA (3290)	AA (2800)
rs16832091	45045989		AA (2385)	AA (3236)	AA (3236)	AA (3236)	AA (3290)	AA (2800)
rs270707	45049458		BB (2385)	BB (3236)	BB (3236)	BB (3236)	BB (3290)	BB (2800)
rs270709	45050047		BB (2385)	BB (3236)	BB (3236)	BB (3236)	BB (3290)	BB (2800)
rs270716	45054866		AA (2385)	AA (3236)	AA (3236)	AA (3236)	AA (3290)	AA (2800)
rs156650	45056293		AA (2385)	AA (3236)	AA (3236)	AA (3236)	AA (3290)	AA (2800)
rs17842391	45064759		AA (2385)	AA (3236)	AA (3236)	AA (3236)	AA (3290)	AA (2800)
rs17383073	45070142		AA (2385)	AA (3236)	AA (3236)	AA (3236)	AA (3290)	AA (2800)
rs149844	45070565		BB (2385)	BB (3236)	BB (3236)	BB (3236)	BB (3290)	BB (2800)
rs270738	45071196		AA (2385)	AA (3236)	AA (3236)	AA (3236)	AA (3290)	AA (2800)
rs459160	45080932		BB (2385)	BB (3236)	BB (3236)	BB (3236)	BB (3290)	BB (2800)
rs457397	45081766		BB (2385)	BB (3236)	BB (3236)	BB (3236)	BB (3290)	BB (2800)
rs3768439	45113326		BB (2385)	BB (3236)	BB (3236)	BB (3236)	BB (3290)	BB (2800)
rs41524944	45122025		AA (2385)	AA (3236)	AA (3236)	AA (3236)	AA (3290)	AA (2800)
rs41468751	45127184		BB (2385)	BB (3236)	BB (3236)	BB (3236)	BB (3290)	BB (2800)
rs2224403	45150220		AA (2385)	AA (3236)	AA (3236)	AA (3236)	AA (3290)	AA (2800)
rs1321703	45157738		BB (2385)	BB (3236)	BB (3236)	BB (3236)	BB (3290)	BB (2800)
rs2355710	45166513		BB (2385)	BB (3236)	BB (3236)	BB (3236)	BB (3290)	BB (2800)
rs2180765	45167143		AA (2385)	AA (3236)	AA (3236)	AA (3236)	AA (3290)	AA (2800)
rs2983713	45168726		AA (2385)	AA (3236)	AA (3236)	AA (3236)	AA (3290)	AA (2800)
rs10890313	45175925		AA (2385)	AA (3236)	AA (3236)	AA (3236)	AA (3290)	AA (2800)
rs11211026	45187285		BB (2385)	BB (3236)	BB (3236)	BB (3236)	BB (3290)	BB (2800)
rs6689911	45219952		AA (2385)	AA (3236)	AA (3236)	AA (3236)	AA (3290)	AA (2800)
rs17391430	45234389		BB (2385)	BB (3236)	BB (3236)	BB (3236)	BB (3290)	BB (2800)
rs6676749	45264545		BB (2385)	BB (3236)	BB (3236)	BB (3236)	BB (3290)	BB (2800)
rs11573588	45293090		AA (2385)	AA (3236)	AA (3236)	AA (3236)	AA (3290)	AA (2800)
rs16832263	45299050		AA (2385)	AA (3236)	AA (3236)	AA (3236)	AA (3290)	AA (2800)
rs7517439	45312867		BB (2385)	BB (3236)	BB (3236)	BB (3236)	BB (3290)	BB (2800)
rs11211046	45321563		BB (2385)	BB (3236)	BB (3236)	BB (3236)	BB (3290)	BB (2800)
rs16832301	45327716		AA (2385)	AA (3236)	AA (3236)	AA (3236)	AA (3290)	AA (2800)
rs17390398	45333256		AA (2385)	AA (3236)	AA (3236)	AA (3236)	AA (3290)	AA (2800)
rs17392632	45333719		AA (2385)	AA (3236)	AA (3236)	AA (3236)	AA (3290)	AA (2800)
rs263975	45358527		BB (2385)	BB (3236)	BB (3236)	BB (3236)	BB (3290)	BB (2800)
rs263977	45360287		AA (2385)	AA (3236)	AA (3236)	AA (3236)	AA (3290)	AA (2800)
rs3767490	45360658		BB (2385)	BB (3236)	BB (3236)	BB (3236)	BB (3290)	BB (2800)
rs10890315	45362445		BB (2385)	BB (3236)	BB (3236)	BB (3236)	BB (3290)	BB (2800)
rs263978	45362992		AA (2385)	AA (3236)	AA (3236)	AA (3236)	AA (3290)	AA (2800)
rs608649	45363887		BB (2385)	BB (3236)	BB (3236)	BB (3236)	BB (3290)	BB (2800)
rs7531019	45365834		AA (2385)	AA (3236)	AA (3236)	AA (3236)	AA (3290)	AA (2800)
rs1572379	45369086		BB (2385)	BB (3236)	BB (3236)	BB (3236)	BB (3290)	BB (2800)
rs3903059	45392955		AA (2385)	AA (3236)	AA (3236)	AA (3236)	AA (3290)	AA (2800)

rs12735637	45394369	BB (2385)	BB (3236)	BB (3236)	BB (3236)	BB (3290)	BB (2800)
rs12125367	45396919	AA (2385)	AA (3236)	AA (3236)	AA (3236)	AA (3290)	AA (2800)
rs7529213	45419613	BB (2385)	BB (3236)	BB (3236)	BB (3236)	BB (3290)	BB (2800)
rs264002	45423943	BB (2385)	BB (3236)	BB (3236)	BB (3236)	BB (3290)	BB (2800)
rs11211059	45430218	BB (2385)	BB (3236)	BB (3236)	BB (3236)	BB (3290)	BB (2800)
rs11556200	45444038	AA (2385)	AA (3236)	AA (3236)	AA (3236)	AA (3290)	AA (2800)
rs264025	45444996	BB (2385)	BB (3236)	BB (3236)	BB (3236)	BB (3290)	BB (2800)
rs7535347	45466511	AA (2385)	AA (3236)	AA (3236)	AA (3236)	AA (3290)	AA (2800)
rs7541207	45474008	BB (2385)	BB (3236)	BB (3236)	BB (3236)	BB (3290)	BB (2800)
rs2236576	45477675	BB (2385)	BB (3236)	BB (3236)	BB (3236)	BB (3290)	BB (2800)
rs6678726	45496395	BB (2385)	BB (3236)	BB (3236)	BB (3236)	BB (3290)	BB (2800)
rs346732	45585348	BB (2385)	BB (3236)	BB (3236)	BB (3236)	BB (3290)	BB (2800)
rs1226757	45592886	BB (2385)	BB (3236)	BB (3236)	BB (3236)	BB (3290)	BB (2800)
rs346696	45596447	BB (2385)	BB (3236)	BB (3236)	BB (3236)	BB (3290)	BB (2800)
rs11585488	45617312	BB (2385)	BB (3236)	BB (3236)	BB (3236)	BB (3290)	BB (2800)
rs1698295	45635802	BB (2385)	BB (3236)	BB (3236)	BB (3236)	BB (3290)	BB (2800)
rs2997465	45641454	AA (2385)	AA (3236)	AA (3236)	AA (3236)	AA (3290)	AA (2800)
rs3009973	45665580	AA (2385)	AA (3236)	AA (3236)	AA (3236)	AA (3290)	AA (2800)
rs1938302	45677818	AA (2385)	AA (3236)	AA (3236)	AA (3236)	AA (3290)	AA (2800)
rs34398124	45697068	BB (2385)	BB (3236)	BB (3236)	BB (3236)	BB (3290)	BB (2800)
rs17192059	45699200	AA (2385)	AA (3236)	AA (3236)	AA (3236)	AA (3290)	AA (2800)
rs6688723	45763542	AA (2385)	AA (3236)	AA (3236)	AA (3236)	AA (3290)	AA (2800)
rs7548931	45766192	BB (2385)	BB (3236)	BB (3236)	BB (3236)	BB (3290)	BB (2800)
rs7535487	45791189	AA (2385)	AA (3236)	AA (3236)	AA (3236)	AA (3290)	AA (2800)
rs3219487	45798555	AA (2385)	AA (3236)	AA (3236)	AA (3236)	AA (3290)	AA (2800)
rs9429072	45810091	BB (2385)	BB (3236)	BB (3236)	BB (3236)	BB (3290)	BB (2800)
rs2153608	45813941	BB (2385)	BB (3236)	BB (3236)	BB (3236)	BB (3290)	BB (2800)
rs2185549	45814103	BB (2385)	BB (3236)	BB (3236)	BB (3236)	BB (3290)	BB (2800)
rs4660849	45819872	BB (2385)	BB (3236)	BB (3236)	BB (3236)	BB (3290)	BB (2800)
rs9326141	45828943	BB (2385)	BB (3236)	BB (3236)	BB (3236)	BB (3290)	BB (2800)
rs7543428	45839643	AA (2385)	AA (3236)	AA (3236)	AA (3236)	AA (3290)	AA (2800)
rs11211101	45849311	AA (2385)	AA (3236)	AA (3236)	AA (3236)	AA (3290)	AA (2800)
rs2487442	45857176	BB (2385)	BB (3236)	BB (3236)	BB (3236)	BB (3290)	BB (2800)
rs1826691	45881262	BB (2385)	BB (3236)	BB (3236)	BB (3236)	BB (3290)	BB (2800)
rs1771551	45883643	AA (2385)	AA (3236)	AA (3236)	AA (3236)	AA (3290)	AA (2800)
rs41343546	45908263	AA (2385)	AA (3236)	AA (3236)	AA (3236)	AA (3290)	AA (2800)
rs12743512	45954033	BB (2385)	BB (3236)	BB (3236)	BB (3236)	BB (3290)	BB (2800)
rs2275276	45973928	AA (2385)	AA (3236)	AA (3236)	AA (3236)	AA (3290)	AA (2800)
rs4660306	45978675	BB (2385)	BB (3236)	BB (3236)	BB (3236)	BB (3290)	BB (2800)
rs16832557	45978923	AA (2385)	AA (3236)	AA (3236)	AA (3236)	AA (3290)	AA (2800)
rs6699702	45981834	AA (2385)	AA (3236)	AA (3236)	AA (3236)	AA (3290)	AA (2800)
rs10736426	45984353	BB (2385)	BB (3236)	BB (3236)	BB (3236)	BB (3290)	BB (2800)
rs7522705	45992300	BB (2385)	BB (3236)	BB (3236)	BB (3236)	BB (3290)	BB (2800)
rs945179	45992460	BB (2385)	BB (3236)	BB (3236)	BB (3236)	BB (3290)	BB (2800)
rs6429569	46001330	AA (2385)	AA (3236)	AA (3236)	AA (3236)	AA (3290)	AA (2800)
rs487174	46009536	AA (2385)	AA (3236)	AA (3236)	AA (3236)	AA (3290)	AA (2800)
rs868351	46009818	AA (2385)	AA (3236)	AA (3236)	AA (3236)	AA (3290)	AA (2800)
rs669417	46026737	AA (2385)	AA (3236)	AA (3236)	AA (3236)	AA (3290)	AA (2800)
rs3014216	46035760	BB (2385)	BB (3236)	BB (3236)	BB (3236)	BB (3290)	BB (2800)
rs1084086	46041100	BB (2385)	BB (3236)	BB (3236)	BB (3236)	BB (3290)	BB (2800)

rs3014210	46066608	BB (2385)	BB (3236)	BB (3236)	BB (3236)	BB (3290)	BB (2800)
rs17410308	46078191	BB (2385)	BB (3236)	BB (3236)	BB (3236)	BB (3290)	BB (2800)
rs1972410	46080947	AA (2385)	AA (3236)	AA (3236)	AA (3236)	AA (3290)	AA (2800)
rs2991986	46081282	BB (2385)	BB (3236)	BB (3236)	BB (3236)	BB (3290)	BB (2800)
rs1547924	46085112	AA (2385)	AA (3236)	AA (3236)	AA (3236)	AA (3290)	AA (2800)
rs1547925	46085356	BB (2385)	BB (3236)	BB (3236)	BB (3236)	BB (3290)	BB (2800)
rs2275086	46085879	BB (2385)	BB (3236)	BB (3236)	BB (3236)	BB (3290)	BB (2800)
rs3014245	46086913	BB (2385)	BB (3236)	BB (3236)	BB (3236)	BB (3290)	BB (2800)
rs11211145	46108491	AA (2385)	AA (3236)	AA (3236)	AA (3236)	AA (3290)	AA (2800)
rs5021934	46109304	AA (2385)	AA (3236)	AA (3236)	AA (3236)	AA (3290)	AA (2800)
rs4660313	46110841	BB (2385)	BB (3236)	BB (3236)	BB (3236)	BB (3290)	BB (2800)
rs4660880	46111040	AA (2385)	AA (3236)	AA (3236)	AA (3236)	AA (3290)	AA (2800)
rs11211150	46112693	BB (2385)	BB (3236)	BB (3236)	BB (3236)	BB (3290)	BB (2800)
rs6662999	46119351	BB (2385)	BB (3236)	BB (3236)	BB (3236)	BB (3290)	BB (2800)
rs11211176	46223086	AA (2385)	AA (3236)	AA (3236)	AA (3236)	AA (3290)	AA (2800)
rs6702764	46252717	AA (2385)	AA (3236)	AA (3236)	AA (3236)	AA (3290)	AA (2800)
rs6661163	46255004	AA (2385)	AA (3236)	AA (3236)	AA (3236)	AA (3290)	AA (2800)
rs7539800	46262129	BB (2385)	BB (3236)	BB (3236)	BB (3236)	BB (3290)	BB (2800)
rs4660316	46273183	BB (2385)	BB (3236)	BB (3236)	BB (3236)	BB (3290)	BB (2800)
rs11211200	46288352	AA (2385)	AA (3236)	AA (3236)	AA (3236)	AA (3290)	AA (2800)
rs10789477	46306583	AA (2385)	AA (3236)	AA (3236)	AA (3236)	AA (3290)	AA (2800)
rs10749859	46314170	BB (2385)	BB (3236)	BB (3236)	BB (3236)	BB (3290)	BB (2800)
rs10890361	46346234	AA (2385)	AA (3236)	AA (3236)	AA (3236)	AA (3290)	AA (2800)
rs11211214	46347328	AA (2385)	AA (3236)	AA (3236)	AA (3236)	AA (3290)	AA (2800)
rs12045409	46358720	BB (2385)	BB (3236)	BB (3236)	BB (3236)	BB (3290)	BB (2800)
rs10890365	46359348	AA (2385)	AA (3236)	AA (3236)	AA (3236)	AA (3290)	AA (2800)
rs4545281	46360054	AA (2385)	AA (3236)	AA (3236)	AA (3236)	AA (3290)	AA (2800)
rs7556436	46363880	BB (2385)	BB (3236)	BB (3236)	BB (3236)	BB (3290)	BB (2800)
rs10890370	46389217	AA (2385)	AA (3236)	AA (3236)	AA (3236)	AA (3290)	AA (2800)
rs10890373	46393755	AA (2385)	AA (3236)	AA (3236)	AA (3236)	AA (3290)	AA (2800)
rs10789486	46408667	AA (2385)	AA (3236)	AA (3236)	AA (3236)	AA (3290)	AA (2800)
rs6700322	46409176	AA (2385)	AA (3236)	AA (3236)	AA (3236)	AA (3290)	AA (2800)
rs10890378	46410657	BB (2385)	BB (3236)	BB (3236)	BB (3236)	BB (3290)	BB (2800)
rs11211232	46411146	AA (2385)	AA (3236)	AA (3236)	AA (3236)	AA (3290)	AA (2800)
rs4660905	46461587	BB (2385)	BB (3236)	BB (3236)	BB (3236)	BB (3290)	BB (2800)
rs4660334	46462126	AA (2385)	AA (3236)	AA (3236)	AA (3236)	AA (3290)	AA (2800)
rs4073846	46462881	BB (2385)	BB (3236)	BB (3236)	BB (3236)	BB (3290)	BB (2800)
rs4073847	46463012	BB (2385)	BB (3236)	BB (3236)	BB (3236)	BB (3290)	BB (2800)
rs11579634	46466391	BB (2385)	BB (3236)	BB (3236)	BB (3236)	BB (3290)	BB (2800)
rs6677777	46475836	AA (2385)	AA (3236)	AA (3236)	AA (3236)	AA (3290)	AA (2800)
rs11211247	46476587	BB (2385)	BB (3236)	BB (3236)	BB (3236)	BB (3290)	BB (2800)
rs946527	46485970	AA (2385)	AA (3236)	AA (3236)	AA (3236)	AA (3290)	AA (2800)
rs946526	46487168	BB (2385)	BB (3236)	BB (3236)	BB (3236)	BB (3290)	BB (2800)
rs785480	46492164	AA (2385)	AA (3236)	AA (3236)	AA (3236)	AA (3290)	AA (2800)
rs2236560	46495434	AA (2385)	AA (3236)	AA (3236)	AA (3236)	AA (3290)	AA (2800)
rs1768809	46502836	AA (2385)	AA (3236)	AA (3236)	AA (3236)	AA (3290)	AA (2800)
rs7538978	46505054	BB (2385)	BB (3236)	BB (3236)	BB (3236)	BB (3290)	BB (2800)
rs1707322	46505147	AA (2385)	AA (3236)	AA (3236)	AA (3236)	AA (3290)	AA (2800)
rs1707321	46505309	AA (2385)	AA (3236)	AA (3236)	AA (3236)	AA (3290)	AA (2800)
rs4388641	46522326	BB (2385)	BB (3236)	BB (3236)	BB (3236)	BB (3290)	BB (2800)

rs785465	46522577	AA (2385)	AA (3236)	AA (3236)	AA (3236)	AA (3290)	AA (2800)
rs1613296	46546852	BB (2385)	BB (3236)	BB (3236)	BB (3236)	BB (3290)	BB (2800)
rs785495	46587887	AA (2385)	AA (3236)	AA (3236)	AA (3236)	AA (3290)	AA (2800)
rs41378047	46602011	AA (2385)	AA (3236)	AA (3236)	AA (3236)	AA (3290)	AA (2800)
rs2486445	46605369	AA (2385)	AA (3236)	AA (3236)	AA (3236)	AA (3290)	AA (2800)
rs41401147	46608657	BB (2385)	BB (3236)	BB (3236)	BB (3236)	BB (3290)	BB (2800)
rs1983261	46617981	BB (2385)	BB (3236)	BB (3236)	BB (3236)	BB (3290)	BB (2800)
rs17102023	46618634	AA (2385)	AA (3236)	AA (3236)	AA (3236)	AA (3290)	AA (2800)
rs3855959	46633874	AA (2385)	AA (3236)	AA (3236)	AA (3236)	AA (3290)	AA (2800)
rs3845300	46635149	BB (2385)	BB (3236)	BB (3236)	BB (3236)	BB (3290)	BB (2800)
rs7538871	46671997	BB (2385)	BB (3236)	BB (3236)	BB (3236)	BB (3290)	BB (2800)
rs17102086	46722939	AA (2385)	AA (3236)	AA (3236)	AA (3236)	AA (3290)	AA (2800)
rs17102091	46733105	BB (2385)	BB (3236)	BB (3236)	BB (3236)	BB (3290)	BB (2800)
rs4660921	46781602	BB (2385)	BB (3236)	BB (3236)	BB (3236)	BB (3290)	BB (2800)
rs17102133	46782192	BB (2385)	BB (3236)	BB (3236)	BB (3236)	BB (3290)	BB (2800)
rs17102152	46810763	BB (2385)	BB (3236)	BB (3236)	BB (3236)	BB (3290)	BB (2800)
rs6684274	46810842	AA (2385)	AA (3236)	AA (3236)	AA (3236)	AA (3290)	AA (2800)
rs6659228	46811027	BB (2385)	BB (3236)	BB (3236)	BB (3236)	BB (3290)	BB (2800)
rs17361805	46813967	BB (2385)	BB (3236)	BB (3236)	BB (3236)	BB (3290)	BB (2800)
rs12385696	46818836	BB (2385)	BB (3236)	BB (3236)	BB (3236)	BB (3290)	BB (2800)
rs41534051	46828734	BB (2385)	BB (3236)	BB (3236)	BB (3236)	BB (3290)	BB (2800)
rs10252	46830430	BB (2385)	BB (3236)	BB (3236)	BB (3236)	BB (3290)	BB (2800)
rs12062	46830447	AA (2385)	AA (3236)	AA (3236)	AA (3236)	AA (3290)	AA (2800)
rs17361887	46834775	BB (2385)	BB (3236)	BB (3236)	BB (3236)	BB (3290)	BB (2800)
rs6695043	46844530	AA (2385)	AA (3236)	AA (3236)	AA (3236)	AA (3290)	AA (2800)
rs2145408	46861907	BB (2385)	BB (3236)	BB (3236)	BB (3236)	BB (3290)	BB (2800)
rs4141964	46865040	BB (2385)	BB (3236)	BB (3236)	BB (3236)	BB (3290)	BB (2800)
rs324425	46881353	AA (2385)	AA (3236)	AA (3236)	AA (3236)	AA (3290)	AA (2800)
rs324423	46885104	BB (2385)	BB (3236)	BB (3236)	BB (3236)	BB (3290)	BB (2800)
rs1571138	46895641	BB (2385)	BB (3236)	BB (3236)	BB (3236)	BB (3290)	BB (2800)
rs1150066	46901546	AA (2385)	AA (3236)	AA (3236)	AA (3236)	AA (3290)	AA (2800)
rs619512	46905067	AA (2385)	AA (3236)	AA (3236)	AA (3236)	AA (3290)	AA (2800)
rs7542528	46920087	BB (2385)	BB (3236)	BB (3236)	BB (3236)	BB (3290)	BB (2800)
rs7532468	46920331	AA (2385)	AA (3236)	AA (3236)	AA (3236)	AA (3290)	AA (2800)
rs10890398	46925594	AA (2385)	AA (3236)	AA (3236)	AA (3236)	AA (3290)	AA (2800)
rs10890401	46936423	AA (2385)	AA (3236)	AA (3236)	AA (3236)	AA (3290)	AA (2800)
rs11211287	46939999	AA (2385)	AA (3236)	AA (3236)	AA (3236)	AA (3290)	AA (2800)
rs1886118	46949071	AA (2385)	AA (3236)	AA (3236)	AA (3236)	AA (3290)	AA (2800)
rs753209	46957260	BB (2385)	BB (3236)	BB (3236)	BB (3236)	BB (3290)	BB (2800)
rs11211292	46960553	BB (2385)	BB (3236)	BB (3236)	BB (3236)	BB (3290)	BB (2800)
rs12562748	46961914	BB (2385)	BB (3236)	BB (3236)	BB (3236)	BB (3290)	BB (2800)
rs2025382	46969018	AA (2385)	AA (3236)	AA (3236)	AA (3236)	AA (3290)	AA (2800)
rs35270239	46972958	BB (2385)	BB (3236)	BB (3236)	BB (3236)	BB (3290)	BB (2800)
rs11803942	46973075	AA (2385)	AA (3236)	AA (3236)	AA (3236)	AA (3290)	AA (2800)
rs6683116	46975773	BB (2385)	BB (3236)	BB (3236)	BB (3236)	BB (3290)	BB (2800)
rs942249	46982455	BB (2385)	BB (3236)	BB (3236)	BB (3236)	BB (3290)	BB (2800)
rs1745371	46985192	AA (2385)	AA (3236)	AA (3236)	AA (3236)	AA (3290)	AA (2800)
rs17102247	46989472	BB (2385)	BB (3236)	BB (3236)	BB (3236)	BB (3290)	BB (2800)
rs17102252	46989531	AA (2385)	AA (3236)	AA (3236)	AA (3236)	AA (3290)	AA (2800)
rs1267305	46989742	BB (2385)	BB (3236)	BB (3236)	BB (3236)	BB (3290)	BB (2800)

rs2404611	46997127	AA (2385)	AA (3236)	AA (3236)	AA (3236)	AA (3290)	AA (2800)
rs12138736	46999758	BB (2385)	BB (3236)	BB (3236)	BB (3236)	BB (3290)	BB (2800)
rs3737741	47000566	BB (2385)	BB (3236)	BB (3236)	BB (3236)	BB (3290)	BB (2800)
rs2476163	47007844	AA (2385)	AA (3236)	AA (3236)	AA (3236)	AA (3290)	AA (2800)
rs885127	47012144	AA (2385)	AA (3236)	AA (3236)	AA (3236)	AA (3290)	AA (2800)
rs942079	47012702	BB (2385)	BB (3236)	BB (3236)	BB (3236)	BB (3290)	BB (2800)
rs1258042	47015073	BB (2385)	BB (3236)	BB (3236)	BB (3236)	BB (3290)	BB (2800)
rs1258050	47022760	BB (2385)	BB (3236)	BB (3236)	BB (3236)	BB (3290)	BB (2800)
rs2181414	47033849	BB (2385)	BB (3236)	BB (3236)	BB (3236)	BB (3290)	BB (2800)
rs1933934	47034928	AA (2385)	AA (3236)	AA (3236)	AA (3236)	AA (3290)	AA (2800)
rs1054743	47035643	AA (2385)	AA (3236)	AA (3236)	AA (3236)	AA (3290)	AA (2800)
rs3766239	47036076	BB (2385)	BB (3236)	BB (3236)	BB (3236)	BB (3290)	BB (2800)
rs3766238	47036315	BB (2385)	BB (3236)	BB (3236)	BB (3236)	BB (3290)	BB (2800)
rs17102303	47049049	BB (2385)	BB (3236)	BB (3236)	BB (3236)	BB (3290)	BB (2800)
rs1258022	47055339	AA (2385)	AA (3236)	AA (3236)	AA (3236)	AA (3290)	AA (2800)
rs956527	47055992	BB (2385)	BB (3236)	BB (3236)	BB (3236)	BB (3290)	BB (2800)
rs3766231	47056347	AA (2385)	AA (3236)	AA (3236)	AA (3236)	AA (3290)	AA (2800)
rs2181412	47096647	BB (2385)	BB (3236)	BB (3236)	BB (3236)	BB (3290)	BB (2800)
rs1258072	47098048	AA (2385)	AA (3236)	AA (3236)	AA (3236)	AA (3290)	AA (2800)
rs744096	47107390	AA (2385)	AA (3236)	AA (3236)	AA (3236)	AA (3290)	AA (2800)
rs736188	47108828	BB (2385)	BB (3236)	BB (3236)	BB (3236)	BB (3290)	BB (2800)
rs1933932	47109328	AA (2385)	AA (3236)	AA (3236)	AA (3236)	AA (3290)	AA (2800)
rs629412	47120673	BB (2385)	BB (3236)	BB (3236)	BB (3236)	BB (3290)	BB (2800)
rs11211334	47121419	AA (2385)	AA (3236)	AA (3236)	AA (3236)	AA (3290)	AA (2800)
rs631368	47121502	AA (2385)	AA (3236)	AA (3236)	AA (3236)	AA (3290)	AA (2800)
rs611468	47124479	BB (2385)	BB (3236)	BB (3236)	BB (3236)	BB (3290)	BB (2800)
rs7354865	47124999	BB (2385)	BB (3236)	BB (3236)	BB (3236)	BB (3290)	BB (2800)
rs682000	47125185	AA (2385)	AA (3236)	AA (3236)	AA (3236)	AA (3290)	AA (2800)
rs601060	47139580	AA (2385)	AA (3236)	AA (3236)	AA (3236)	AA (3290)	AA (2800)
rs7412469	47146533	AA (2385)	AA (3236)	AA (3236)	AA (3236)	AA (3290)	AA (2800)
rs2275380	47147728	AA (2385)	AA (3236)	AA (3236)	AA (3236)	AA (3290)	AA (2800)
rs1890473	47149794	BB (2385)	BB (3236)	BB (3236)	BB (3236)	BB (3290)	BB (2800)
rs2119301	47153547	BB (2385)	BB (3236)	BB (3236)	BB (3236)	BB (3290)	BB (2800)
rs2241863	47155098	AA (2385)	AA (3236)	AA (3236)	AA (3236)	AA (3290)	AA (2800)
rs3766218	47157306	BB (2385)	BB (3236)	BB (3236)	BB (3236)	BB (3290)	BB (2800)
rs3766217	47159109	AA (2385)	AA (3236)	AA (3236)	AA (3236)	AA (3290)	AA (2800)
rs1440487	47167075	AA (2385)	AA (3236)	AA (3236)	AA (3236)	AA (3290)	AA (2800)
rs10749863	47174328	BB (2385)	BB (3236)	BB (3236)	BB (3236)	BB (3290)	BB (2800)
rs10158678	47176606	BB (2385)	BB (3236)	BB (3236)	BB (3236)	BB (3290)	BB (2800)
rs12071983	47178065	AA (2385)	AA (3236)	AA (3236)	AA (3236)	AA (3290)	AA (2800)
rs3766211	47184196	BB (2385)	BB (3236)	BB (3236)	BB (3236)	BB (3290)	BB (2800)
rs4660957	47188150	AA (2385)	AA (3236)	AA (3236)	AA (3236)	AA (3290)	AA (2800)
rs2993410	47212009	BB (2385)	BB (3236)	BB (3236)	BB (3236)	BB (3290)	BB (2800)
rs17102489	47212148	AA (2385)	AA (3236)	AA (3236)	AA (3236)	AA (3290)	AA (2800)
rs594387	47213386	AA (2385)	AA (3236)	AA (3236)	AA (3236)	AA (3290)	AA (2800)
rs720413	47219823	BB (2385)	BB (3236)	BB (3236)	BB (3236)	BB (3290)	BB (2800)
rs12082811	47225886	BB (2385)	BB (3236)	BB (3236)	BB (3236)	BB (3290)	BB (2800)
rs17449603	47233733	AA (2385)	AA (3236)	AA (3236)	AA (3236)	AA (3290)	AA (2800)
rs17102519	47234550	AA (2385)	AA (3236)	AA (3236)	AA (3236)	AA (3290)	AA (2800)
rs681840	47268041	BB (2385)	BB (3236)	BB (3236)	BB (3236)	BB (3290)	BB (2800)

rs837395	47269338	AA (2385)	AA (3236)	AA (3236)	AA (3236)	AA (3290)	AA (2800)
rs2065996	47275241	BB (2385)	BB (3236)	BB (3236)	BB (3236)	BB (3290)	BB (2800)
rs4646491	47280884	BB (2385)	BB (3236)	BB (3236)	BB (3236)	BB (3290)	BB (2800)
rs3766197	47281827	BB (2385)	BB (3236)	BB (3236)	BB (3236)	BB (3290)	BB (2800)
rs17102644	47318400	AA (2385)	AA (3236)	AA (3236)	AA (3236)	AA (3290)	AA (2800)
rs9429100	47325376	AA (2385)	AA (3236)	AA (3236)	AA (3236)	AA (3290)	AA (2800)
rs3890011	47398743	BB (2385)	BB (3236)	BB (3236)	BB (3236)	BB (3290)	BB (2800)
rs9333002	47402838	AA (2385)	AA (3236)	AA (3236)	AA (3236)	AA (3290)	AA (2800)
rs9332982	47406178	AA (2385)	AA (3236)	AA (3236)	AA (3236)	AA (3290)	AA (2800)
rs11211414	47449065	BB (2385)	BB (3236)	BB (3236)	BB (3236)	BB (3290)	BB (2800)
rs9326151	47485172	AA (2385)	AA (3236)	AA (3236)	AA (3236)	AA (3290)	AA (2800)
rs1502909	47485824	AA (2385)	AA (3236)	AA (3236)	AA (3236)	AA (3290)	AA (2800)
rs10890445	47486392	BB (2385)	BB (3236)	BB (3236)	BB (3236)	BB (3290)	BB (2800)
rs9793716	47493584	AA (2385)	AA (3236)	AA (3236)	AA (3236)	AA (3290)	AA (2800)
rs9793989	47496738	AA (2385)	AA (3236)	AA (3236)	AA (3236)	AA (3290)	AA (2800)
rs11211420	47497037	BB (2385)	BB (3236)	BB (3236)	BB (3236)	BB (3290)	BB (2800)
rs2292059	47504717	AA (2385)	AA (3236)	AA (3236)	AA (3236)	AA (3290)	AA (2800)
rs9793202	47533837	AA (2385)	AA (3236)	AA (3236)	AA (3236)	AA (3290)	AA (2800)
rs11211439	47561118	BB (2385)	BB (3236)	BB (3236)	BB (3236)	BB (3290)	BB (2800)
rs11211451	47573413	BB (2385)	BB (3236)	BB (3236)	BB (3236)	BB (3290)	BB (2800)
rs10890459	47573980	AA (2385)	AA (3236)	AA (3236)	AA (3236)	AA (3290)	AA (2800)
rs12734327	47574650	BB (2385)	BB (3236)	BB (3236)	BB (3236)	BB (3290)	BB (2800)
rs17109741	47606517	AA (2385)	AA (3236)	AA (3236)	AA (3236)	AA (3290)	AA (2800)
rs11211470	47615899	BB (2385)	BB (3236)	BB (3236)	BB (3236)	BB (3290)	BB (2800)
rs2798353	47641041	AA (2385)	AA (3236)	AA (3236)	AA (3236)	AA (3290)	AA (2800)
rs2494248	47644902	AA (2385)	AA (3236)	AA (3236)	AA (3236)	AA (3290)	AA (2800)
rs12184246	47657141	AA (2385)	AA (3236)	-3236	AA (3236)	AB (3290)	-2800
rs17103015	47666146	BB (2385)	BB (3236)	BB (3236)	BB (3236)	BB (3290)	BB (2800)
rs17103016	47666546	BB (2385)	BB (3236)	BB (3236)	BB (3236)	BB (3290)	BB (2800)
rs741959	47676233	BB (2385)	BB (3236)	BB (3236)	BB (3236)	BB (3290)	BB (2800)
rs2758743	47678355	AA (2385)	AA (3236)	AA (3236)	AA (3236)	AA (3290)	AA (2800)
rs2070929	47683607	AA (2385)	AA (3236)	AA (3236)	AA (3236)	AA (3290)	AA (2800)
rs7534271	47693981	BB (2385)	BB (3236)	BB (3236)	BB (3236)	BB (3290)	BB (2800)
rs2798349	47698703	AA (2385)	AA (3236)	AA (3236)	AA (3236)	AA (3290)	AA (2800)
rs11211483	47707435	BB (2385)	BB (3236)	BB (3236)	BB (3236)	BB (3290)	BB (2800)
rs10890472	47708112	AA (2385)	AA (3236)	AA (3236)	AA (3236)	AA (3290)	AA (2800)
rs1028646	47718358	AA (2385)	AA (3236)	AA (3236)	AA (3236)	AA (3290)	AA (2800)
rs12123358	47723824	AA (2385)	AA (3236)	AA (3236)	AA (3236)	AA (3290)	AA (2800)
rs6588095	47724166	BB (2385)	BB (3236)	BB (3236)	BB (3236)	BB (3290)	BB (2800)
rs11211500	47732378	BB (2385)	BB (3236)	BB (3236)	BB (3236)	BB (3290)	BB (2800)
rs2821096	47742726	AA (2385)	AA (3236)	AA (3236)	AA (3236)	AA (3290)	AA (2800)
rs11211503	47743189	BB (2385)	BB (3236)	BB (3236)	BB (3236)	BB (3290)	BB (2800)
rs6588196	47743490	BB (2385)	BB (3236)	BB (3236)	BB (3236)	BB (3290)	BB (2800)
rs11587014	47763207	BB (2385)	BB (3236)	BB (3236)	BB (3236)	BB (3290)	BB (2800)
rs12141060	47766647	BB (2385)	BB (3236)	BB (3236)	BB (3236)	BB (3290)	BB (2800)
rs6697248	47766943	BB (2385)	BB (3236)	BB (3236)	BB (3236)	BB (3290)	BB (2800)
rs6703956	47768743	BB (2385)	BB (3236)	BB (3236)	BB (3236)	BB (3290)	BB (2800)
rs3925058	47797623	AA (2385)	AA (3236)	AA (3236)	AA (3236)	AA (3290)	AA (2800)
rs12123269	47806087	BB (2385)	BB (3236)	BB (3236)	BB (3236)	BB (3290)	BB (2800)
rs2406102	47857735	BB (2385)	BB (3236)	BB (3236)	BB (3236)	BB (3290)	BB (2800)

rs6699343	47867739		AA (2385)	AA (3236)	AA (3236)	AA (3236)	AA (3290)	AA (2800)
rs7555040	47869316		BB (2385)	BB (3236)	BB (3236)	BB (3236)	BB (3290)	BB (2800)
rs4603175	47874586		BB (2385)	BB (3236)	BB (3236)	BB (3236)	BB (3290)	BB (2800)
rs17103223	47874863		BB (2385)	BB (3236)	BB (3236)	BB (3236)	BB (3290)	BB (2800)
rs11578776	47880278		AA (2385)	AA (3236)	AA (3236)	AA (3236)	AA (3290)	AA (2800)
rs6697911	47907604		AA (2385)	AA (3236)	AA (3236)	AA (3236)	AA (3290)	AA (2800)
rs1316443	47907662		AA (2385)	AA (3236)	AA (3236)	AA (3236)	AA (3290)	AA (2800)
rs6665067	47914863		AA (2385)	AA (3236)	AA (3236)	AA (3236)	AA (3290)	AA (2800)
rs527430	47918821		BB (2385)	BB (3236)	BB (3236)	BB (3236)	BB (3290)	BB (2800)
rs10890481	47922264		AA (2385)	AA (3236)	AA (3236)	AA (3236)	AA (3290)	AA (2800)
rs524158	47933094		BB (2385)	BB (3236)	BB (3236)	BB (3236)	BB (3290)	BB (2800)
rs2097	47933363		BB (2385)	BB (3236)	BB (3236)	BB (3236)	BB (3290)	BB (2800)
rs10789509	47938129		BB (2385)	BB (3236)	BB (3236)	BB (3236)	BB (3290)	BB (2800)
rs545670	47942422		AA (2385)	AA (3236)	AA (3236)	AA (3236)	AA (3290)	AA (2800)
rs2065945	47948764		BB (2385)	BB (3236)	BB (3236)	BB (3236)	BB (3290)	BB (2800)
rs12125691	47954511		BB (2385)	BB (3236)	BB (3236)	BB (3236)	BB (3290)	BB (2800)
rs17371253	47955427		AA (2385)	AA (3236)	AA (3236)	AA (3236)	AA (3290)	AA (2800)
rs7528714	47966058		AA (2385)	AA (3236)	AA (3236)	AA (3236)	AA (3290)	AA (2800)
rs7547288	47966292		AA (2385)	AA (3236)	AA (3236)	AA (3236)	AA (3290)	AA (2800)
rs17103342	47966860		AA (2385)	AA (3236)	AA (3236)	AA (3236)	AA (3290)	AA (2800)
rs4926559	47971646		BB (2385)	BB (3236)	BB (3236)	BB (3236)	BB (3290)	BB (2800)
rs1572547	47972149		AA (2385)	AA (3236)	AA (3236)	AA (3236)	AA (3290)	AA (2800)
rs1415540	47982642		AA (2385)	AA (3236)	AA (3236)	AA (3236)	AA (3290)	AA (2800)
rs1024108	47990749		AA (2385)	AA (3236)	AA (3236)	AA (3236)	AA (3290)	AA (2800)
rs478526	47991951		AA (2385)	AA (3236)	AA (3236)	AA (3236)	AA (3290)	AA (2800)
rs11211546	47992211		BB (2385)	BB (3236)	BB (3236)	BB (3236)	BB (3290)	BB (2800)
rs2102437	47992260		AA (2385)	AA (3236)	AA (3236)	AA (3236)	AA (3290)	AA (2800)
rs11211547	47995198		AA (2385)	AA (3236)	AA (3236)	AA (3236)	AA (3290)	AA (2800)
rs6688849	47996204		AA (2385)	AA (3236)	AA (3236)	AA (3236)	AA (3290)	AA (2800)
rs2485439	48004776		AA (2385)	AA (3236)	AA (3236)	AA (3236)	AA (3290)	AA (2800)
rs518057	48009686		AA (2385)	AA (3236)	AA (3236)	AA (3236)	AA (3290)	AA (2800)
rs694308	48015258		AA (2385)	AA (3236)	AA (3236)	AA (3236)	AA (3290)	AA (2800)
rs17420782	48019222		AA (2385)	AA (3236)	AA (3236)	AA (3236)	AA (3290)	AA (2800)
rs2485446	48019888		AA (2385)	AA (3236)	AA (3236)	AA (3236)	AA (3290)	AA (2800)
rs2457070	48024773		AA (2385)	AA (3236)	AA (3236)	AA (3236)	AA (3290)	AA (2800)
rs623229	48027067		BB (2385)	BB (3236)	BB (3236)	BB (3236)	BB (3290)	BB (2800)
rs1567310	48028705		BB (2385)	BB (3236)	BB (3236)	BB (3236)	BB (3290)	BB (2800)
rs2174957	48028843		AA (2385)	AA (3236)	AA (3236)	AA (3236)	AA (3290)	AA (2800)
rs11211555	48029039		AA (2385)	AA (3236)	AA (3236)	AA (3236)	AA (3290)	AA (2800)
rs6656330	48029727		BB (2385)	BB (3236)	BB (3236)	BB (3236)	BB (3290)	BB (2800)
rs17455010	48033714		BB (2385)	BB (3236)	BB (3236)	BB (3236)	BB (3290)	BB (2800)
rs2457068	48033995		BB (2385)	BB (3236)	BB (3236)	BB (3236)	BB (3290)	BB (2800)
rs1876599	48034735		BB (2385)	BB (3236)	BB (3236)	BB (3236)	BB (3290)	BB (2800)
rs949540	48036324		BB (2385)	BB (3236)	BB (3236)	BB (3236)	BB (3290)	BB (2800)
rs1508604	48046929		AA (2385)	AA (3236)	AA (3236)	AA (3236)	AA (3290)	AA (2800)
rs1355063	48048690		BB (2385)	BB (3236)	BB (3236)	BB (3236)	BB (3290)	BB (2800)
rs1108866	48060089		AA (2385)	AA (3236)	AA (3236)	AA (3236)	AA (3290)	AA (2800)
rs1108865	48060109		BB (2385)	BB (3236)	BB (3236)	BB (3236)	BB (3290)	BB (2800)
rs12402772	48062278		AA (2385)	AA (3236)	AA (3236)	AA (3236)	AA (3290)	AA (2800)
rs12742768	48065019		BB (2385)	BB (3236)	BB (3236)	BB (3236)	BB (3290)	BB (2800)

rs12760556	48065157		BB (2385)	BB (3236)	BB (3236)	BB (3236)	BB (3290)	BB (2800)
rs4926915	48083479		AA (2385)	AA (3236)	AA (3236)	AA (3236)	AA (3290)	AA (2800)
rs11211579	48095100		AA (2385)	AA (3236)	AA (3236)	AA (3236)	AA (3290)	AA (2800)
rs2506987	48100829		BB (2385)	BB (3236)	BB (3236)	BB (3236)	BB (3290)	BB (2800)
rs931937	48102121		AA (2385)	AA (3236)	AA (3236)	AA (3236)	AA (3290)	AA (2800)
rs12410488	48105228		AA (2385)	AA (3236)	AA (3236)	AA (3236)	AA (3290)	AA (2800)
rs10493128	48110751		BB (2385)	BB (3236)	BB (3236)	BB (3236)	BB (3290)	BB (2800)
rs4926925	48113798		BB (2385)	BB (3236)	BB (3236)	BB (3236)	BB (3290)	BB (2800)
rs10493127	48114417		AA (2385)	AA (3236)	AA (3236)	AA (3236)	AA (3290)	AA (2800)
rs11211585	48117693		AA (2385)	AA (3236)	AA (3236)	AA (3236)	AA (3290)	AA (2800)
rs12407853	48118188		BB (2385)	BB (3236)	BB (3236)	BB (3236)	BB (3290)	BB (2800)
rs11211590	48168375		BB (2385)	BB (3236)	BB (3236)	BB (3236)	BB (3290)	BB (2800)
rs893758	48181286		BB (2385)	BB (3236)	BB (3236)	BB (3236)	BB (3290)	BB (2800)
rs17103679	48183520		BB (2385)	BB (3236)	BB (3236)	BB (3236)	BB (3290)	BB (2800)
rs7523973	48189631		BB (2385)	BB (3236)	BB (3236)	BB (3236)	BB (3290)	BB (2800)
rs1992299	48191605		AA (2385)	AA (3236)	AA (3236)	AA (3236)	AA (3290)	AA (2800)
rs4926601	48193205		BB (2385)	BB (3236)	BB (3236)	BB (3236)	BB (3290)	BB (2800)
rs2165193	48213641		AA (2385)	AA (3236)	AA (3236)	AA (3236)	AA (3290)	AA (2800)
rs4504937	48214207		AA (2385)	AA (3236)	AA (3236)	AA (3236)	AA (3290)	AA (2800)
rs17103715	48214623		AA (2385)	AA (3236)	AA (3236)	AA (3236)	AA (3290)	AA (2800)
rs6700815	48214882		BB (2385)	BB (3236)	BB (3236)	BB (3236)	BB (3290)	BB (2800)
rs6701002	48215064		BB (2385)	BB (3236)	BB (3236)	BB (3236)	BB (3290)	BB (2800)
rs11579242	48215379		AA (2385)	AA (3236)	AA (3236)	AA (3236)	AA (3290)	AA (2800)
rs6665005	48215587		AA (2385)	AA (3236)	AA (3236)	AA (3236)	AA (3290)	AA (2800)
rs6672725	48215610		AA (2385)	AA (3236)	AA (3236)	AA (3236)	AA (3290)	AA (2800)
rs17424028	48217969		BB (2385)	BB (3236)	BB (3236)	BB (3236)	BB (3290)	BB (2800)
rs11584807	48220454		AA (2385)	AA (3236)	AA (3236)	AA (3236)	AA (3290)	AA (2800)
rs1077842	48224582		AA (2385)	AA (3236)	AA (3236)	AA (3236)	AA (3290)	AA (2800)
rs12085966	48229395		AA (2385)	AA (3236)	AA (3236)	AA (3236)	AA (3290)	AA (2800)
rs3850879	48232131		BB (2385)	BB (3236)	BB (3236)	BB (3236)	BB (3290)	BB (2800)
rs3844086	48232503		BB (2385)	BB (3236)	BB (3236)	BB (3236)	BB (3290)	BB (2800)
rs2282361	48240005		BB (2385)	BB (3236)	BB (3236)	BB (3236)	BB (3290)	BB (2800)
rs17458210	48240289		BB (2385)	BB (3236)	BB (3236)	BB (3236)	BB (3290)	BB (2800)
rs3850885	48247077		AA (2385)	AA (3236)	AA (3236)	AA (3236)	AA (3290)	AA (2800)
rs6588505	48254782		AA (2385)	AA (3236)	AA (3236)	AA (3236)	AA (3290)	AA (2800)
rs6588506	48255008		AA (2385)	AA (3236)	AA (3236)	AA (3236)	AA (3290)	AA (2800)
rs2119102	48266640		AA (2385)	AA (3236)	AA (3236)	AA (3236)	AA (3290)	AA (2800)
rs11211606	48269325		BB (2385)	BB (3236)	BB (3236)	BB (3236)	BB (3290)	BB (2800)
rs875398	48275263		AA (2385)	AA (3236)	AA (3236)	AA (3236)	AA (3290)	AA (2800)
rs6703502	48277765		BB (2385)	BB (3236)	BB (3236)	BB (3236)	BB (3290)	BB (2800)
rs17424871	48279623		BB (2385)	BB (3236)	BB (3236)	BB (3236)	BB (3290)	BB (2800)
rs11211609	48282667		AA (2385)	AA (3236)	AA (3236)	AA (3236)	AA (3290)	AA (2800)
rs11211610	48283620		AA (2385)	AA (3236)	AA (3236)	AA (3236)	AA (3290)	AA (2800)
rs12134443	48286563		AA (2385)	AA (3236)	AA (3236)	AA (3236)	AA (3290)	AA (2800)
rs11581081	48294120		AA (2385)	AA (3236)	AA (3236)	AA (3236)	AA (3290)	AA (2800)
rs12129836	48308457		BB (2385)	BB (3236)	BB (3236)	BB (3236)	BB (3290)	BB (2800)
rs10890507	48309587		BB (2385)	BB (3236)	BB (3236)	BB (3236)	BB (3290)	BB (2800)
rs6588525	48310335		BB (2385)	BB (3236)	BB (3236)	BB (3236)	BB (3290)	BB (2800)
rs11211616	48311619		AA (2385)	AA (3236)	AA (3236)	AA (3236)	AA (3290)	AA (2800)
rs10890510	48334490		AA (2385)	AA (3236)	AA (3236)	AA (3236)	AA (3290)	AA (2800)

rs1556979	48337061	AA (2385)	AA (3236)	AA (3236)	AA (3236)	AA (3290)	AA (2800)
rs1556980	48337492	BB (2385)	BB (3236)	BB (3236)	BB (3236)	BB (3290)	BB (2800)
rs1417138	48338817	BB (2385)	BB (3236)	BB (3236)	BB (3236)	BB (3290)	BB (2800)
rs10493130	48341005	BB (2385)	BB (3236)	BB (3236)	BB (3236)	BB (3290)	BB (2800)
rs6657569	48341451	BB (2385)	BB (3236)	BB (3236)	BB (3236)	BB (3290)	BB (2800)
rs17425308	48341893	BB (2385)	BB (3236)	BB (3236)	BB (3236)	BB (3290)	BB (2800)
rs10890511	48344390	BB (2385)	BB (3236)	BB (3236)	BB (3236)	BB (3290)	BB (2800)
rs9729312	48350799	BB (2385)	BB (3236)	BB (3236)	BB (3236)	BB (3290)	BB (2800)
rs7527840	48362380	BB (2385)	BB (3236)	BB (3236)	BB (3236)	BB (3290)	BB (2800)
rs4927198	48363281	BB (2385)	BB (3236)	BB (3236)	BB (3236)	BB (3290)	BB (2800)
rs4926671	48364782	BB (2385)	BB (3236)	BB (3236)	BB (3236)	BB (3290)	BB (2800)
rs2201691	48367400	AA (2385)	AA (3236)	AA (3236)	AA (3236)	AA (3290)	AA (2800)
rs11211631	48383741	AA (2385)	AA (3236)	AA (3236)	AA (3236)	AA (3290)	AA (2800)
rs4927231	48383980	BB (2385)	BB (3236)	BB (3236)	BB (3236)	BB (3290)	BB (2800)
rs10493131	48387427	BB (2385)	BB (3236)	BB (3236)	BB (3236)	BB (3290)	BB (2800)
rs6669798	48387588	BB (2385)	BB (3236)	BB (3236)	BB (3236)	BB (3290)	BB (2800)
rs1361443	48391668	AA (2385)	AA (3236)	AA (3236)	AA (3236)	AA (3290)	AA (2800)
rs17459747	48396869	AA (2385)	AA (3236)	AA (3236)	AA (3236)	AA (3290)	AA (2800)
rs951879	48397737	BB (2385)	BB (3236)	BB (3236)	BB (3236)	BB (3290)	BB (2800)
rs17426042	48407275	BB (2385)	BB (3236)	BB (3236)	BB (3236)	BB (3290)	BB (2800)
rs11578428	48411232	BB (2385)	BB (3236)	BB (3236)	BB (3236)	BB (3290)	BB (2800)
rs1603530	48411729	BB (2385)	BB (3236)	BB (3236)	BB (3236)	BB (3290)	BB (2800)
rs931628	48419646	AA (2385)	AA (3236)	AA (3236)	AA (3236)	AA (3290)	AA (2800)
rs9660630	48421627	AA (2385)	AA (3236)	AA (3236)	AA (3236)	AA (3290)	AA (2800)
rs6673069	48421694	AA (2385)	AA (3236)	AA (3236)	AA (3236)	AA (3290)	AA (2800)
rs17103971	48434109	AA (2385)	AA (3236)	AA (3236)	AA (3236)	AA (3290)	AA (2800)
rs17103980	48445208	AA (2385)	AA (3236)	AA (3236)	AA (3236)	AA (3290)	AA (2800)
rs17103991	48445491	BB (2385)	BB (3236)	BB (3236)	BB (3236)	BB (3290)	BB (2800)
rs6676465	48457543	BB (2385)	BB (3236)	BB (3236)	BB (3236)	BB (3290)	BB (2800)
rs41394646	48467450	BB (2385)	BB (3236)	BB (3236)	BB (3236)	BB (3290)	BB (2800)
rs6695419	48479075	AA (2385)	AA (3236)	AA (3236)	AA (3236)	AA (3290)	AA (2800)
rs303916	48479525	BB (2385)	BB (3236)	BB (3236)	BB (3236)	BB (3290)	BB (2800)
rs2492791	48484327	AA (2385)	AA (3236)	AA (3236)	AA (3236)	AA (3290)	AA (2800)
rs1413330	48485047	BB (2385)	BB (3236)	BB (3236)	BB (3236)	BB (3290)	BB (2800)
rs1693246	48486172	BB (2385)	BB (3236)	BB (3236)	BB (3236)	BB (3290)	BB (2800)
rs1693251	48487137	AA (2385)	AA (3236)	AA (3236)	AA (3236)	AA (3290)	AA (2800)
rs215863	48495636	AA (2385)	AA (3236)	AA (3236)	AA (3236)	AA (3290)	AA (2800)
rs303925	48503280	BB (2385)	BB (3236)	BB (3236)	BB (3236)	BB (3290)	BB (2800)
rs6588642	48507037	BB (2385)	BB (3236)	BB (3236)	BB (3236)	BB (3290)	BB (2800)
rs748142	48509053	BB (2385)	BB (3236)	BB (3236)	BB (3236)	BB (3290)	BB (2800)
rs748141	48509159	BB (2385)	BB (3236)	BB (3236)	BB (3236)	BB (3290)	BB (2800)
rs6664402	48509939	AA (2385)	AA (3236)	AA (3236)	AA (3236)	AA (3290)	AA (2800)
rs6690144	48512369	AA (2385)	AA (3236)	AA (3236)	AA (3236)	AA (3290)	AA (2800)
rs823390	48512745	BB (2385)	BB (3236)	BB (3236)	BB (3236)	BB (3290)	BB (2800)
rs513108	48516401	AA (2385)	AA (3236)	AA (3236)	AA (3236)	AA (3290)	AA (2800)
rs4927321	48517018	AA (2385)	AA (3236)	AA (3236)	AA (3236)	AA (3290)	AA (2800)
rs302694	48517182	AA (2385)	AA (3236)	AA (3236)	AA (3236)	AA (3290)	AA (2800)
rs302691	48519912	BB (2385)	BB (3236)	BB (3236)	BB (3236)	BB (3290)	BB (2800)
rs6699629	48520617	AA (2385)	AA (3236)	AA (3236)	AA (3236)	AA (3290)	AA (2800)
rs2270973	48520777	BB (2385)	BB (3236)	BB (3236)	BB (3236)	BB (3290)	BB (2800)

rs6679531	48524180		BB (2385)	BB (3236)	BB (3236)	BB (3236)	BB (3290)	BB (2800)
rs7542715	48527534		AA (2385)	AA (3236)	AA (3236)	AA (3236)	AA (3290)	AA (2800)
rs728864	48530258		AA (2385)	AA (3236)	AA (3236)	AA (3236)	AA (3290)	AA (2800)
rs592072	48543158		BB (2385)	BB (3236)	BB (3236)	BB (3236)	BB (3290)	BB (2800)
rs653364	48545337		AA (2385)	AA (3236)	AA (3236)	AA (3236)	AA (3290)	AA (2800)
rs17468833	48564267		AA (2385)	AA (3236)	AA (3236)	AA (3236)	AA (3290)	AA (2800)
rs17468972	48568400		BB (2385)	BB (3236)	BB (3236)	BB (3236)	BB (3290)	BB (2800)
rs10888596	48568672		BB (2385)	BB (3236)	BB (3236)	BB (3236)	BB (3290)	BB (2800)
rs12127052	48581510		AA (2385)	AA (3236)	AA (3236)	AA (3236)	AA (3290)	AA (2800)
rs11205444	48604697		BB (2385)	BB (3236)	BB (3236)	BB (3236)	BB (3290)	BB (2800)
rs214215	48628136		BB (2385)	BB (3236)	BB (3236)	BB (3236)	BB (3290)	BB (2800)
rs214214	48628867		AA (2385)	AA (3236)	AA (3236)	AA (3236)	AA (3290)	AA (2800)
rs10788882	48630438		BB (2385)	BB (3236)	BB (3236)	BB (3236)	BB (3290)	BB (2800)
rs17104225	48630940		AA (2385)	AA (3236)	AA (3236)	AA (3236)	AA (3290)	AA (2800)
rs11205449	48647124		AA (2385)	AA (3236)	AA (3236)	AA (3236)	AA (3290)	AA (2800)
rs12123285	48651709		BB (2385)	BB (3236)	BB (3236)	BB (3236)	BB (3290)	BB (2800)
rs7541597	48655214		BB (2385)	BB (3236)	BB (3236)	BB (3236)	BB (3290)	BB (2800)
rs519720	48666524		AA (2385)	AA (3236)	AA (3236)	AA (3236)	AA (3290)	AA (2800)
rs558615	48668297		BB (2385)	BB (3236)	BB (3236)	BB (3236)	BB (3290)	BB (2800)
rs555238	48668616		BB (2385)	BB (3236)	BB (3236)	BB (3236)	BB (3290)	BB (2800)
rs12128169	48670415		BB (2385)	BB (3236)	BB (3236)	BB (3236)	BB (3290)	BB (2800)
rs17104273	48670484		AA (2385)	AA (3236)	AA (3236)	AA (3236)	AA (3290)	AA (2800)
rs492379	48673745		BB (2385)	BB (3236)	BB (3236)	BB (3236)	BB (3290)	BB (2800)
rs537303	48673936		AB (2385)	AB (3236)	AB (3236)	-3236	-3290	AB (2800)
rs12081912	48681693		AA (2385)	AA (3236)	AA (3236)	AA (3236)	AA (3290)	AA (2800)
rs1524732	48686096		AA (2385)	AA (3236)	AA (3236)	AA (3236)	AA (3290)	AA (2800)
rs850763	48708228		AA (2385)	AA (3236)	AA (3236)	AA (3236)	AA (3290)	AA (2800)
rs6670286	48709019		BB (2385)	BB (3236)	BB (3236)	BB (3236)	BB (3290)	BB (2800)
rs41341650	48711815		BB (2385)	BB (3236)	BB (3236)	BB (3236)	BB (3290)	BB (2800)
rs498502	48725096		BB (2385)	BB (3236)	BB (3236)	BB (3236)	BB (3290)	BB (2800)
rs6704171	48725431		BB (2385)	BB (3236)	BB (3236)	BB (3236)	BB (3290)	BB (2800)
rs926978	48732780		BB (2385)	BB (3236)	BB (3236)	BB (3236)	BB (3290)	BB (2800)
rs743065	48734366		BB (2385)	BB (3236)	BB (3236)	BB (3236)	BB (3290)	BB (2800)
rs212968	48734666		AA (2385)	AA (3236)	AA (3236)	AA (3236)	AA (3290)	AA (2800)
rs4433447	48759274		BB (2385)	BB (3236)	BB (3236)	BB (3236)	BB (3290)	BB (2800)
rs7536253	48775943		BB (2385)	BB (3236)	BB (3236)	BB (3236)	BB (3290)	BB (2800)
rs1114192	48797332		BB (2385)	BB (3236)	BB (3236)	BB (3236)	BB (3290)	BB (2800)
rs1120985	48810112		BB (2385)	BB (3236)	BB (3236)	BB (3236)	BB (3290)	BB (2800)
rs11205476	48820294		AA (2385)	AA (3236)	AA (3236)	AA (3236)	AA (3290)	AA (2800)
rs7513258	48826009		BB (2385)	BB (3236)	BB (3236)	BB (3236)	BB (3290)	BB (2800)
rs3767616	48857313		AA (2385)	AA (3236)	AA (3236)	AA (3236)	AA (3290)	AA (2800)
rs1416043	48863365		AA (2385)	AA (3236)	AA (3236)	AA (3236)	AA (3290)	AA (2800)
rs2490537	48889599		BB (2385)	BB (3236)	BB (3236)	BB (3236)	BB (3290)	BB (2800)
rs2485911	48910976		BB (2385)	BB (3236)	BB (3236)	BB (3236)	BB (3290)	BB (2800)
rs2255264	48911430		BB (2385)	BB (3236)	BB (3236)	BB (3236)	BB (3290)	BB (2800)
rs2803251	48911793		BB (2385)	BB (3236)	BB (3236)	BB (3236)	BB (3290)	BB (2800)
rs2787873	48932488		AB (2385)	AA (3236)	AA (3236)	AA (3236)	AA (3290)	AA (2800)
rs2245912	48939678		BB (2385)	BB (3236)	BB (3236)	BB (3236)	BB (3290)	BB (2800)
rs1539530	48974933		AA (2385)	AA (3236)	AA (3236)	AA (3236)	AA (3290)	AA (2800)
rs2354462	48975446		BB (2385)	BB (3236)	BB (3236)	BB (3236)	BB (3290)	BB (2800)

rs7527001	48976856	BB (2385)	BB (3236)	BB (3236)	BB (3236)	BB (3290)	BB (2800)
rs7555069	48987511	BB (2385)	BB (3236)	BB (3236)	BB (3236)	BB (3290)	BB (2800)
rs2798112	48998247	AA (2385)	AA (3236)	AA (3236)	AA (3236)	AA (3290)	AA (2800)
rs2246105	48998361	BB (2385)	BB (3236)	BB (3236)	BB (3236)	BB (3290)	BB (2800)
rs2803261	48998748	AA (2385)	AA (3236)	AA (3236)	AA (3236)	AA (3290)	AA (2800)
rs1539525	49001369	BB (2385)	BB (3236)	BB (3236)	BB (3236)	BB (3290)	BB (2800)
rs2246670	49002874	AA (2385)	AA (3236)	AA (3236)	AA (3236)	AA (3290)	AA (2800)
rs7515225	49006786	AA (2385)	AA (3236)	AA (3236)	AA (3236)	AA (3290)	AA (2800)
rs11205505	49017435	AA (2385)	AA (3236)	AA (3236)	AA (3236)	AA (3290)	AA (2800)
rs2803267	49019793	AA (2385)	AA (3236)	AA (3236)	AA (3236)	AA (3290)	AA (2800)
rs1418953	49021080	BB (2385)	BB (3236)	BB (3236)	BB (3236)	BB (3290)	BB (2800)
rs12066734	49021519	BB (2385)	BB (3236)	BB (3236)	BB (3236)	BB (3290)	BB (2800)
rs10888606	49025933	AA (2385)	AA (3236)	AA (3236)	AA (3236)	AA (3290)	AA (2800)
rs2803270	49031238	AA (2385)	AA (3236)	AA (3236)	AA (3236)	AA (3290)	AA (2800)
rs17104515	49033296	AA (2385)	AA (3236)	AA (3236)	AA (3236)	AA (3290)	AA (2800)
rs11802042	49033734	AA (2385)	AA (3236)	AA (3236)	AA (3236)	AA (3290)	AA (2800)
rs2798098	49035783	BB (2385)	BB (3236)	BB (3236)	BB (3236)	BB (3290)	BB (2800)
rs1539529	49037053	AA (2385)	AA (3236)	AA (3236)	AA (3236)	AA (3290)	AA (2800)
rs17373183	49037217	AA (2385)	AA (3236)	AA (3236)	AA (3236)	AA (3290)	AA (2800)
rs2803273	49042349	BB (2385)	BB (3236)	BB (3236)	BB (3236)	BB (3290)	BB (2800)
rs6665839	49043877	AA (2385)	AA (3236)	AA (3236)	AA (3236)	AA (3290)	AA (2800)
rs2798122	49044129	BB (2385)	BB (3236)	BB (3236)	BB (3236)	BB (3290)	BB (2800)
rs2798121	49046897	BB (2385)	BB (3236)	BB (3236)	BB (3236)	BB (3290)	BB (2800)
rs17104533	49047146	AA (2385)	AA (3236)	AA (3236)	AA (3236)	AA (3290)	AA (2800)
rs17104537	49047168	AA (2385)	AA (3236)	AA (3236)	AA (3236)	AA (3290)	AA (2800)
rs11205512	49051748	AA (2385)	AA (3236)	AA (3236)	AA (3236)	AA (3290)	AA (2800)
rs11579440	49052423	BB (2385)	BB (3236)	BB (3236)	BB (3236)	-3290	BB (2800)
rs2803277	49052933	BB (2385)	BB (3236)	BB (3236)	BB (3236)	BB (3290)	BB (2800)
rs923028	49054880	AA (2385)	AA (3236)	AA (3236)	AA (3236)	AA (3290)	AA (2800)
rs319980	49066644	AA (2385)	AA (3236)	AA (3236)	AA (3236)	AA (3290)	AA (2800)
rs320017	49074995	AA (2385)	AA (3236)	AA (3236)	AA (3236)	AA (3290)	AA (2800)
rs320027	49082023	BB (2385)	BB (3236)	BB (3236)	BB (3236)	BB (3290)	BB (2800)
rs320033	49087602	BB (2385)	BB (3236)	BB (3236)	BB (3236)	BB (3290)	BB (2800)
rs11205517	49092039	AA (2385)	AA (3236)	AA (3236)	AA (3236)	AA (3290)	AA (2800)
rs7517510	49107979	BB (2385)	BB (3236)	BB (3236)	BB (3236)	BB (3290)	BB (2800)
rs319954	49112893	BB (2385)	BB (3236)	BB (3236)	BB (3236)	BB (3290)	BB (2800)
rs17373849	49113247	AA (2385)	AA (3236)	AA (3236)	AA (3236)	AA (3290)	AA (2800)
rs319953	49113319	BB (2385)	BB (3236)	BB (3236)	BB (3236)	BB (3290)	BB (2800)
rs11205519	49117242	BB (2385)	BB (3236)	BB (3236)	BB (3236)	BB (3290)	BB (2800)
rs319998	49123432	BB (2385)	BB (3236)	BB (3236)	BB (3236)	BB (3290)	BB (2800)
rs319999	49123707	BB (2385)	BB (3236)	BB (3236)	BB (3236)	BB (3290)	BB (2800)
rs320000	49126253	AA (2385)	AA (3236)	AA (3236)	AA (3236)	AA (3290)	AA (2800)
rs320009	49129204	BB (2385)	BB (3236)	BB (3236)	BB (3236)	BB (3290)	BB (2800)
rs320011	49129846	BB (2385)	BB (3236)	BB (3236)	BB (3236)	BB (3290)	BB (2800)
rs320012	49130138	AA (2385)	AA (3236)	AA (3236)	AA (3236)	AA (3290)	AA (2800)
rs320013	49130538	AA (2385)	AA (3236)	AA (3236)	AA (3236)	AA (3290)	AA (2800)
rs320014	49130683	AA (2385)	AA (3236)	AA (3236)	AA (3236)	AA (3290)	AA (2800)
rs6663376	49133661	AA (2385)	AA (3236)	AA (3236)	AA (3236)	AA (3290)	AA (2800)
rs387244	49139531	AA (2385)	AA (3236)	AA (3236)	AA (3236)	AA (3290)	AA (2800)
rs7532350	49141251	BB (2385)	BB (3236)	BB (3236)	BB (3236)	BB (3290)	BB (2800)

rs6694041	49148580		BB (2385)	BB (3236)	BB (3236)	BB (3236)	BB (3290)	BB (2800)
rs11205521	49148942		BB (2385)	BB (3236)	BB (3236)	BB (3236)	BB (3290)	BB (2800)
rs17104662	49154950		AA (2385)	AA (3236)	AA (3236)	AA (3236)	AA (3290)	AA (2800)
rs17104664	49156377		BB (2385)	BB (3236)	BB (3236)	BB (3236)	BB (3290)	BB (2800)
rs6695778	49164688		BB (2385)	BB (3236)	BB (3236)	BB (3236)	BB (3290)	BB (2800)
rs320040	49169263		AA (2385)	AA (3236)	AA (3236)	AA (3236)	AA (3290)	AA (2800)
rs320046	49170356		BB (2385)	BB (3236)	BB (3236)	BB (3236)	BB (3290)	BB (2800)
rs320047	49171141		AA (2385)	AA (3236)	AA (3236)	AA (3236)	AA (3290)	AA (2800)
rs320049	49174059		BB (2385)	BB (3236)	BB (3236)	BB (3236)	BB (3290)	BB (2800)
rs320050	49174405		BB (2385)	BB (3236)	BB (3236)	BB (3236)	BB (3290)	BB (2800)
rs753456	49187171		AA (2385)	AA (3236)	AA (3236)	AA (3236)	AA (3290)	AA (2800)
rs4926528	49189775		BB (2385)	BB (3236)	BB (3236)	BB (3236)	BB (3290)	BB (2800)
rs6588054	49206305		AA (2385)	AA (3236)	AA (3236)	AA (3236)	AA (3290)	AA (2800)
rs1473455	49210570		AA (2385)	AA (3236)	AA (3236)	AA (3236)	AA (3290)	AA (2800)
rs11578117	49219560		AA (2385)	AA (3236)	AA (3236)	AA (3236)	AA (3290)	AA (2800)
rs12086219	49221303		AA (2385)	AA (3236)	AA (3236)	AA (3236)	AA (3290)	AA (2800)
rs10493135	49222528		AA (2385)	AA (3236)	AA (3236)	AA (3236)	AA (3290)	AA (2800)
rs1934395	49234376		AA (2385)	AA (3236)	AA (3236)	AA (3236)	AA (3290)	AA (2800)
rs11205538	49237594		AA (2385)	AA (3236)	AA (3236)	AA (3236)	AA (3290)	AA (2800)
rs1934377	49237975		AA (2385)	AA (3236)	AA (3236)	AA (3236)	AA (3290)	AA (2800)
rs2153324	49239968		BB (2385)	BB (3236)	BB (3236)	BB (3236)	BB (3290)	BB (2800)
rs7520784	49240957		BB (2385)	BB (3236)	BB (3236)	BB (3236)	BB (3290)	BB (2800)
rs1934379	49241421		AA (2385)	AA (3236)	AA (3236)	AA (3236)	AA (3290)	AA (2800)
rs7514802	49268066		AA (2385)	AA (3236)	AA (3236)	AA (3236)	AA (3290)	AA (2800)
rs7538351	49270823		BB (2385)	BB (3236)	BB (3236)	BB (3236)	BB (3290)	BB (2800)
rs17104898	49271149		AA (2385)	AA (3236)	AA (3236)	AA (3236)	AA (3290)	AA (2800)
rs12072244	49271311		AA (2385)	AA (3236)	AA (3236)	AA (3236)	AA (3290)	AA (2800)
rs1934367	49273578		AA (2385)	AA (3236)	AA (3236)	AA (3236)	AA (3290)	AA (2800)
rs1934368	49274047		AA (2385)	AA (3236)	AA (3236)	AA (3236)	AA (3290)	AA (2800)
rs2153323	49283412		BB (2385)	BB (3236)	BB (3236)	BB (3236)	BB (3290)	BB (2800)
rs7545104	49288623		AA (2385)	AA (3236)	AA (3236)	AA (3236)	AA (3290)	AA (2800)
rs5015171	49308013		BB (2385)	BB (3236)	BB (3236)	BB (3236)	BB (3290)	BB (2800)
rs12239493	49308088		AA (2385)	AA (3236)	AA (3236)	AA (3236)	AA (3290)	AA (2800)
rs4926765	49315866		AA (2385)	AA (3236)	AA (3236)	AA (3236)	AA (3290)	AA (2800)
rs1890729	49316712		AA (2385)	AA (3236)	AA (3236)	AA (3236)	AA (3290)	AA (2800)
rs17104994	49317185		BB (2385)	BB (3236)	BB (3236)	BB (3236)	BB (3290)	BB (2800)
rs10788896	49317239		BB (2385)	BB (3236)	BB (3236)	BB (3236)	BB (3290)	BB (2800)
rs2355689	49320832		BB (2385)	BB (3236)	BB (3236)	BB (3236)	BB (3290)	BB (2800)
rs6684570	49330804		BB (2385)	BB (3236)	BB (3236)	BB (3236)	BB (3290)	BB (2800)
rs10888629	49331291		AA (2385)	AA (3236)	AA (3236)	AA (3236)	AA (3290)	AA (2800)
rs10888631	49331466		AA (2385)	AA (3236)	AA (3236)	AA (3236)	AA (3290)	AA (2800)
rs9660009	49335597		BB (2385)	BB (3236)	BB (3236)	BB (3236)	BB (3290)	BB (2800)
rs5017079	49336456		AA (2385)	AA (3236)	AA (3236)	AA (3236)	AA (3290)	AA (2800)
rs4363465	49340738		AA (2385)	AA (3236)	AA (3236)	AA (3236)	AA (3290)	AA (2800)
rs4457622	49346828		BB (2385)	BB (3236)	BB (3236)	BB (3236)	BB (3290)	BB (2800)
rs4285747	49352177		AA (2385)	AA (3236)	AA (3236)	AA (3236)	AA (3290)	AA (2800)
rs12023014	49352725		BB (2385)	BB (3236)	BB (3236)	BB (3236)	BB (3290)	BB (2800)
rs4926771	49354711		AA (2385)	AA (3236)	AA (3236)	AA (3236)	AA (3290)	AA (2800)
rs11578524	49355640		AA (2385)	AA (3236)	AA (3236)	AA (3236)	AA (3290)	AA (2800)
rs3121522	49359873		BB (2385)	BB (3236)	BB (3236)	BB (3236)	BB (3290)	BB (2800)

rs6657415	49360162		BB (2385)	BB (3236)	BB (3236)	BB (3236)	BB (3290)	BB (2800)
rs12043418	49360620		AA (2385)	AA (3236)	AA (3236)	AA (3236)	AA (3290)	AA (2800)
rs12033195	49378801		AA (2385)	AA (3236)	AA (3236)	AA (3236)	AA (3290)	AA (2800)
rs1578844	49379812		BB (2385)	BB (3236)	BB (3236)	BB (3236)	BB (3290)	BB (2800)
rs17105173	49380708		BB (2385)	BB (3236)	BB (3236)	BB (3236)	BB (3290)	BB (2800)
rs1891846	49384055		AA (2385)	AA (3236)	AA (3236)	AA (3236)	AA (3290)	AA (2800)
rs1108427	49385466		AA (2385)	AA (3236)	AA (3236)	AA (3236)	AA (3290)	AA (2800)
rs11205572	49390336		AA (2385)	AA (3236)	AA (3236)	AA (3236)	AA (3290)	AA (2800)
rs726814	49400815		BB (2385)	BB (3236)	BB (3236)	BB (3236)	BB (3290)	BB (2800)
rs10788901	49401185		BB (2385)	BB (3236)	BB (3236)	BB (3236)	BB (3290)	BB (2800)
rs10493139	49407397		AA (2385)	AA (3236)	AA (3236)	AA (3236)	AA (3290)	AA (2800)
rs3127547	49422094		AA (2385)	AA (3236)	AA (3236)	AA (3236)	AA (3290)	AA (2800)
rs12097797	49428303		BB (2385)	BB (3236)	BB (3236)	BB (3236)	BB (3290)	BB (2800)
rs7527167	49429425		BB (2385)	BB (3236)	BB (3236)	BB (3236)	BB (3290)	BB (2800)
rs10493138	49429437		AA (2385)	AA (3236)	AA (3236)	AA (3236)	AA (3290)	AA (2800)
rs3122291	49442023		BB (2385)	BB (3236)	BB (3236)	BB (3236)	BB (3290)	BB (2800)
rs3118215	49450704		BB (2385)	BB (3236)	BB (3236)	BB (3236)	BB (3290)	BB (2800)
rs3121514	49451272		BB (2385)	BB (3236)	BB (3236)	BB (3236)	BB (3290)	BB (2800)
rs3118223	49463801		AA (2385)	AA (3236)	AA (3236)	AA (3236)	AA (3290)	AA (2800)
rs3127556	49468025		AA (2385)	AA (3236)	AA (3236)	AA (3236)	AA (3290)	AA (2800)
rs6669433	49482703		BB (2385)	BB (3236)	BB (3236)	BB (3236)	BB (3290)	BB (2800)
rs6672750	49513773		BB (2385)	BB (3236)	BB (3236)	BB (3236)	BB (3290)	BB (2800)
rs7536787	49525465		BB (2385)	BB (3236)	BB (3236)	BB (3236)	BB (3290)	BB (2800)
rs3850871	49528319		AA (2385)	AA (3236)	AA (3236)	AA (3236)	AA (3290)	AA (2800)
rs11205596	49541827		AA (2385)	AA (3236)	AA (3236)	AA (3236)	AA (3290)	AA (2800)
rs6588238	49552036		AA (2385)	AA (3236)	AA (3236)	AA (3236)	AA (3290)	AA (2800)
rs7527364	49566557		AA (2385)	AA (3236)	AA (3236)	AA (3236)	AA (3290)	AA (2800)
rs768328	49573780		BB (2385)	BB (3236)	BB (3236)	BB (3236)	BB (3290)	BB (2800)
rs684382	49588464		AA (2385)	AA (3236)	AA (3236)	AA (3236)	AA (3290)	AA (2800)
rs545621	49589806		BB (2385)	BB (3236)	BB (3236)	BB (3236)	BB (3290)	BB (2800)
rs10493142	49591834		AA (2385)	AA (3236)	AA (3236)	AA (3236)	AA (3290)	AA (2800)
rs483330	49591977		AA (2385)	AA (3236)	AA (3236)	AA (3236)	AA (3290)	AA (2800)
rs354168	49642698		BB (2385)	BB (3236)	BB (3236)	BB (3236)	BB (3290)	BB (2800)
rs354164	49665436		BB (2385)	BB (3236)	BB (3236)	BB (3236)	BB (3290)	BB (2800)
rs6702810	49721079		AA (2385)	AA (3236)	AA (3236)	AA (3236)	AA (3290)	AA (2800)
rs17785166	49731368		AA (2385)	AA (3236)	AA (3236)	AA (3236)	AA (3290)	AA (2800)
rs241501	49765438		AA (2385)	AA (3236)	AA (3236)	AA (3236)	AA (3290)	AA (2800)
rs12037079	49771826		BB (2385)	BB (3236)	BB (3236)	BB (3236)	BB (3290)	BB (2800)
rs241472	49771853		AA (2385)	AA (3236)	AA (3236)	AA (3236)	AA (3290)	AA (2800)
rs1494461	49787196		AA (2385)	AA (3236)	AA (3236)	AA (3236)	AA (3290)	AA (2800)
rs17534292	49797804		AA (2385)	AA (3236)	AA (3236)	AA (3236)	AA (3290)	AA (2800)
rs17105629	49799321		BB (2385)	BB (3236)	BB (3236)	BB (3236)	BB (3290)	BB (2800)
rs17105631	49800063		BB (2385)	BB (3236)	BB (3236)	BB (3236)	BB (3290)	BB (2800)
rs6659225	49822433		AA (2385)	AA (3236)	AA (3236)	AA (3236)	AA (3290)	AA (2800)
rs1338214	49823497		BB (2385)	BB (3236)	BB (3236)	BB (3236)	BB (3290)	BB (2800)
rs17098808	49831380		AA (2385)	AA (3236)	AA (3236)	AA (3236)	AA (3290)	AA (2800)
rs1338216	49847861		AA (2385)	AA (3236)	AA (3236)	AA (3236)	AA (3290)	AA (2800)
rs41480045	49882541		AA (2385)	AA (3236)	AA (3236)	AA (3236)	AA (3290)	AA (2800)
rs1179484	49908644		BB (2385)	BB (3236)	BB (3236)	BB (3236)	BB (3290)	BB (2800)
rs1766377	49917084		BB (2385)	BB (3236)	BB (3236)	BB (3236)	BB (3290)	BB (2800)

rs1167309	49997674	BB (2385)	BB (3236)	BB (3236)	BB (3236)	BB (3290)	BB (2800)
rs2178085	50023956	BB (2385)	BB (3236)	BB (3236)	BB (3236)	BB (3290)	BB (2800)
rs1305490	50025534	BB (2385)	BB (3236)	BB (3236)	BB (3236)	BB (3290)	BB (2800)
rs17105712	50052684	AA (2385)	AA (3236)	AA (3236)	AA (3236)	AA (3290)	AA (2800)
rs6588353	50086359	AA (2385)	AA (3236)	AA (3236)	AA (3236)	AA (3290)	AA (2800)
rs1891667	50095385	AA (2385)	AA (3236)	AA (3236)	AA (3236)	AA (3290)	AA (2800)
rs1343161	50110850	BB (2385)	BB (3236)	BB (3236)	BB (3236)	BB (3290)	BB (2800)
rs12735229	50161291	BB (2385)	BB (3236)	BB (3236)	BB (3236)	BB (3290)	BB (2800)
rs11205641	50185075	AA (2385)	AA (3236)	AA (3236)	AA (3236)	AA (3290)	AA (2800)
rs10888669	50250273	BB (2385)	BB (3236)	BB (3236)	BB (3236)	BB (3290)	BB (2800)
rs4926831	50290101	BB (2385)	BB (3236)	BB (3236)	BB (3236)	BB (3290)	BB (2800)
rs4244636	50290612	BB (2385)	BB (3236)	BB (3236)	BB (3236)	BB (3290)	BB (2800)
rs6675647	50308556	BB (2385)	BB (3236)	BB (3236)	BB (3236)	BB (3290)	BB (2800)
rs4582848	50312200	BB (2385)	BB (3236)	BB (3236)	BB (3236)	BB (3290)	BB (2800)
rs10736388	50335528	BB (2385)	BB (3236)	BB (3236)	BB (3236)	BB (3290)	BB (2800)
rs17105788	50349764	AA (2385)	AA (3236)	AA (3236)	AA (3236)	AA (3290)	AA (2800)
rs6685494	50453735	AA (2385)	AA (3236)	AA (3236)	AA (3236)	AA (3290)	AA (2800)
rs4259707	50528591	AA (2385)	AA (3236)	AA (3236)	AA (3236)	AA (3290)	AA (2800)
rs4636517	50532969	AA (2385)	AA (3236)	AA (3236)	AA (3236)	AA (3290)	AA (2800)
rs4926547	50546810	BB (2385)	BB (3236)	BB (3236)	BB (3236)	BB (3290)	BB (2800)
rs4078484	50580823	AA (2385)	AA (3236)	AA (3236)	AA (3236)	AA (3290)	AA (2800)
rs967582	50582172	AA (2385)	AA (3236)	AA (3236)	AA (3236)	AA (3290)	AA (2800)
rs2494884	50591405	BB (2385)	BB (3236)	BB (3236)	BB (3236)	BB (3290)	BB (2800)
rs4593875	50591851	BB (2385)	BB (3236)	BB (3236)	BB (3236)	BB (3290)	BB (2800)
rs7532622	50594585	BB (2385)	BB (3236)	BB (3236)	BB (3236)	BB (3290)	BB (2800)
rs7523561	50600881	AA (2385)	AA (3236)	AA (3236)	-3236	-3290	AA (2800)
rs6588376	50602495	BB (2385)	BB (3236)	BB (3236)	BB (3236)	BB (3290)	BB (2800)
rs3001635	50643666	AA (2385)	AA (3236)	AA (3236)	AA (3236)	AA (3290)	AA (2800)
rs17105985	50646135	BB (2385)	BB (3236)	BB (3236)	BB (3236)	BB (3290)	BB (2800)
rs3001636	50647026	AA (2385)	AA (3236)	AA (3236)	AA (3236)	AA (3290)	AA (2800)
rs3001637	50648032	AA (2385)	AA (3236)	AA (3236)	AA (3236)	AA (3290)	AA (2800)
rs2494875	50652318	BB (2385)	BB (3236)	BB (3236)	BB (3236)	BB (3290)	BB (2800)
rs17106009	50661138	BB (2385)	BB (3236)	BB (3236)	BB (3236)	BB (3290)	BB (2800)
rs2480682	50661688	BB (2385)	BB (3236)	BB (3236)	BB (3236)	BB (3290)	BB (2800)
rs2494881	50684153	BB (2385)	BB (3236)	BB (3236)	BB (3236)	BB (3290)	BB (2800)
rs2841870	50686434	AA (2385)	AA (3236)	AA (3236)	AA (3236)	-3290	AA (2800)
rs4926849	50694740	BB (2385)	BB (3236)	BB (3236)	BB (3236)	BB (3290)	BB (2800)
rs4926853	50709222	AA (2385)	AA (3236)	AA (3236)	AA (3236)	AA (3290)	AA (2800)
rs4244638	50709557	BB (2385)	BB (3236)	BB (3236)	BB (3236)	BB (3290)	BB (2800)
rs4437920	50716267	AA (2385)	AA (3236)	AA (3236)	AA (3236)	AA (3290)	AA (2800)
rs3009121	50717854	BB (2385)	BB (3236)	BB (3236)	BB (3236)	BB (3290)	BB (2800)
rs3001644	50722989	AA (2385)	AA (3236)	AA (3236)	AA (3236)	AA (3290)	AA (2800)
rs12122035	50726052	AA (2385)	AA (3236)	AA (3236)	AA (3236)	AA (3290)	AA (2800)
rs2938100	50754556	BB (2385)	BB (3236)	BB (3236)	BB (3236)	BB (3290)	BB (2800)
rs3009109	50757885	AA (2385)	AA (3236)	AA (3236)	AA (3236)	AA (3290)	AA (2800)
rs1875635	50765982	BB (2385)	BB (3236)	BB (3236)	BB (3236)	BB (3290)	BB (2800)
rs41372644	50766112	AA (2385)	AA (3236)	AA (3236)	AA (3236)	AA (3290)	AA (2800)
rs1948809	50794187	BB (2385)	BB (3236)	BB (3236)	BB (3236)	BB (3290)	BB (2800)
rs17386024	50808204	AA (2385)	AA (3236)	AA (3236)	AA (3236)	AA (3290)	AA (2800)
rs6657279	50808295	BB (2385)	BB (3236)	BB (3236)	BB (3236)	BB (3290)	BB (2800)

rs11205709	50809294		BB (2385)	BB (3236)	BB (3236)	BB (3236)	BB (3290)	BB (2800)
rs3012088	50809974		AA (2385)	AA (3236)	AA (3236)	AA (3236)	AA (3290)	AA (2800)
rs6660224	50814731		BB (2385)	BB (3236)	BB (3236)	BB (3236)	BB (3290)	BB (2800)
rs1109337	50816390		AA (2385)	AA (3236)	AA (3236)	AA (3236)	AA (3290)	AA (2800)
rs11205712	50817701		AA (2385)	AA (3236)	AA (3236)	AA (3236)	AA (3290)	AA (2800)
rs11205716	50835606		BB (2385)	BB (3236)	BB (3236)	BB (3236)	BB (3290)	BB (2800)
rs2455636	50844173		AA (2385)	AA (3236)	AA (3236)	AA (3236)	AA (3290)	AA (2800)
rs7517924	50844929		BB (2385)	BB (3236)	BB (3236)	BB (3236)	BB (3290)	BB (2800)
rs2055491	50852769		AA (2385)	AA (3236)	AA (3236)	AA (3236)	AA (3290)	AA (2800)
rs4309025	50859745		BB (2385)	BB (3236)	BB (3236)	BB (3236)	BB (3290)	BB (2800)
rs2484676	50872673		BB (2385)	BB (3236)	BB (3236)	BB (3236)	BB (3290)	BB (2800)
rs12097873	50921122		AA (2385)	AA (3236)	AA (3236)	AA (3236)	AA (3290)	AA (2800)
rs12063722	50931102		BB (2385)	BB (3236)	BB (3236)	BB (3236)	BB (3290)	BB (2800)
rs3888843	50935622		BB (2385)	BB (3236)	BB (3236)	BB (3236)	BB (3290)	BB (2800)
rs17106230	50939762		BB (2385)	BB (3236)	BB (3236)	BB (3236)	BB (3290)	BB (2800)
rs1474784	50944634		AA (2385)	AA (3236)	AA (3236)	AA (3236)	AA (3290)	AA (2800)
rs12059456	50954098		AA (2385)	AA (3236)	AA (3236)	AA (3236)	AA (3290)	AA (2800)
rs3789577	50954128		BB (2385)	BB (3236)	BB (3236)	BB (3236)	BB (3290)	BB (2800)
rs1149791	50964040		AA (2385)	AA (3236)	AA (3236)	AA (3236)	AA (3290)	AA (2800)
rs11581155	50976306		AA (2385)	AA (3236)	AA (3236)	AA (3236)	AA (3290)	AA (2800)
rs11205731	51000610		BB (2385)	BB (3236)	BB (3236)	BB (3236)	BB (3290)	BB (2800)
rs17387164	51017469		AA (2385)	AA (3236)	AA (3236)	AA (3236)	AA (3290)	AA (2800)
rs17106287	51025906		AA (2385)	AA (3236)	AA (3236)	AA (3236)	AA (3290)	AA (2800)
rs3789584	51029387		BB (2385)	BB (3236)	BB (3236)	BB (3236)	BB (3290)	BB (2800)
rs12118892	51029609		BB (2385)	BB (3236)	BB (3236)	BB (3236)	BB (3290)	BB (2800)
rs3789586	51054322		BB (2385)	BB (3236)	BB (3236)	BB (3236)	BB (3290)	BB (2800)
rs12083078	51066950		BB (2385)	BB (3236)	BB (3236)	BB (3236)	BB (3290)	BB (2800)
rs12568008	51076822		AA (2385)	AA (3236)	AA (3236)	AA (3236)	AA (3290)	AA (2800)
rs11205743	51087465		BB (2385)	BB (3236)	BB (3236)	BB (3236)	BB (3290)	BB (2800)
rs17106353	51137380		BB (2385)	BB (3236)	BB (3236)	BB (3236)	BB (3290)	BB (2800)
rs11205760	51174330		AA (2385)	AA (3236)	AA (3236)	AA (3236)	AA (3290)	AA (2800)
rs6672112	51188594		AA (2385)	-3236	AA (3236)	-3236	AA (3290)	AA (2800)
rs6680846	51188674		AA (2385)	AA (3236)	AA (3236)	AA (3236)	AA (3290)	AA (2800)
rs12089747	51246155		AA (2385)	AA (3236)	AA (3236)	AA (3236)	AA (3290)	AA (2800)
rs12075991	51252618		AA (2385)	AA (3236)	AA (3236)	AA (3236)	AA (3290)	AA (2800)
rs11205784	51319883		BB (2385)	BB (3236)	BB (3236)	BB (3236)	BB (3290)	BB (2800)
rs1464081	51335094		BB (2385)	BB (3236)	BB (3236)	BB (3236)	BB (3290)	BB (2800)
rs12078434	51393645		AA (2385)	AA (3236)	AA (3236)	AA (3236)	AA (3290)	AA (2800)
rs3176466	51438365		BB (2385)	BB (3236)	BB (3236)	BB (3236)	BB (3290)	BB (2800)
rs41324853	51449575		BB (2385)	BB (3236)	BB (3236)	BB (3236)	BB (3290)	BB (2800)
rs17389502	51468754		BB (2385)	BB (3236)	BB (3236)	BB (3236)	BB (3290)	BB (2800)
rs12092511	51477088		AA (2385)	AA (3236)	AA (3236)	AA (3236)	AA (3290)	AA (2800)
rs6663781	51580637		AA (2385)	AA (3236)	AA (3236)	AA (3236)	AA (3290)	AA (2800)
rs12074413	51616379		AA (2385)	AA (3236)	AA (3236)	AA (3236)	AA (3290)	AA (2800)
rs1999230	51635557		AA (2385)	AA (3236)	AA (3236)	AA (3236)	AA (3290)	AA (2800)
rs17106443	51644702		BB (2385)	BB (3236)	BB (3236)	BB (3236)	BB (3290)	BB (2800)
rs17106481	51748711		BB (2385)	BB (3236)	BB (3236)	BB (3236)	BB (3290)	BB (2800)
rs17106484	51751023		AA (2385)	AA (3236)	AA (3236)	AA (3236)	AA (3290)	AA (2800)
rs11205832	51797643		AA (2385)	AA (3236)	AA (3236)	AA (3236)	AA (3290)	AA (2800)
rs11205836	51804731		BB (2385)	BB (3236)	BB (3236)	BB (3236)	BB (3290)	BB (2800)

rs6588405	51811627		BB (2385)	BB (3236)	BB (3236)	BB (3236)	BB (3290)	BB (2800)
rs6659736	51814274		BB (2385)	BB (3236)	BB (3236)	BB (3236)	BB (3290)	BB (2800)
rs1105681	51837737		BB (2385)	BB (3236)	BB (3236)	BB (3236)	BB (3290)	BB (2800)
rs17106577	51904505		BB (2385)	BB (3236)	BB (3236)	BB (3236)	BB (3290)	BB (2800)
rs17397993	51931124		BB (2385)	BB (3236)	BB (3236)	BB (3236)	BB (3290)	BB (2800)
rs11808604	51964705		AA (2385)	AA (3236)	AA (3236)	AA (3236)	AA (3290)	AA (2800)
rs10399889	52010203		BB (2385)	BB (3236)	BB (3236)	BB (3236)	BB (3290)	BB (2800)
rs12067434	52030379		AA (2385)	AA (3236)	AA (3236)	AA (3236)	AA (3290)	AA (2800)
rs13375698	52059408		AA (2385)	AA (3236)	AA (3236)	AA (3236)	AA (3290)	AA (2800)
rs6702037	52310756		AA (2385)	AA (3236)	AA (3236)	AA (3236)	AA (3290)	AA (2800)
rs11205901	52348349		AA (2385)	AA (3236)	AA (3236)	AA (3236)	AA (3290)	AA (2800)
rs17106862	52348388		BB (2385)	BB (3236)	BB (3236)	BB (3236)	BB (3290)	BB (2800)
rs17106866	52348424		BB (2385)	BB (3236)	BB (3236)	BB (3236)	BB (3290)	BB (2800)
rs11205902	52348769		AA (2385)	AA (3236)	AA (3236)	AA (3236)	AA (3290)	AA (2800)
rs1832703	52361064		AA (2385)	AA (3236)	AA (3236)	AA (3236)	AA (3290)	AA (2800)
rs6588416	52375424		AA (2385)	AA (3236)	AA (3236)	AA (3236)	AA (3290)	AA (2800)
rs2244262	52376769		BB (2385)	BB (3236)	BB (3236)	BB (3236)	BB (3290)	BB (2800)
rs1935289	52406872		AA (2385)	AA (3236)	AA (3236)	AA (3236)	AA (3290)	AA (2800)
rs4557910	52414927		AA (2385)	AA (3236)	AA (3236)	AA (3236)	-3290	AB (2800)
rs2809942	52416078		BB (2385)	BB (3236)	BB (3236)	BB (3236)	BB (3290)	BB (2800)
rs2809943	52416140		BB (2385)	BB (3236)	BB (3236)	BB (3236)	BB (3290)	BB (2800)
rs2795004	52418140		BB (2385)	BB (3236)	BB (3236)	BB (3236)	BB (3290)	BB (2800)
rs2795007	52422863		AA (2385)	AA (3236)	AA (3236)	AA (3236)	AA (3290)	AA (2800)
rs2809949	52425953		BB (2385)	BB (3236)	BB (3236)	BB (3236)	BB (3290)	BB (2800)
rs1167179	52468546		AA (2385)	AA (3236)	AA (3236)	AA (3236)	AA (3290)	AA (2800)
rs2794997	52469594		BB (2385)	BB (3236)	BB (3236)	BB (3236)	BB (3290)	BB (2800)
rs1167183	52474462		AA (2385)	AA (3236)	AA (3236)	AA (3236)	AA (3290)	AA (2800)
rs2992177	52481985		AA (2385)	AA (3236)	AA (3236)	AA (3236)	AA (3290)	AA (2800)
rs7512076	52486104		BB (2385)	BB (3236)	BB (3236)	BB (3236)	BB (3290)	BB (2800)
rs17106988	52499939		BB (2385)	BB (3236)	BB (3236)	BB (3236)	BB (3290)	BB (2800)
rs2809956	52517804		AA (2385)	AA (3236)	AA (3236)	AA (3236)	AA (3290)	AA (2800)
rs1932229	52519028		BB (2385)	BB (3236)	BB (3236)	BB (3236)	BB (3290)	BB (2800)
rs2809955	52519930		BB (2385)	BB (3236)	BB (3236)	BB (3236)	BB (3290)	BB (2800)
rs2783199	52534432		BB (2385)	BB (3236)	BB (3236)	BB (3236)	BB (3290)	BB (2800)
rs17173201	52535322		BB (2385)	BB (3236)	BB (3236)	BB (3236)	BB (3290)	BB (2800)
rs2998737	52535495		BB (2385)	BB (3236)	BB (3236)	BB (3236)	BB (3290)	BB (2800)
rs2985740	52565851		AA (2385)	AA (3236)	AA (3236)	AA (3236)	AA (3290)	AA (2800)
rs12039206	52569002		AA (2385)	AA (3236)	AA (3236)	AA (3236)	AA (3290)	AA (2800)
rs12027960	52584437		BB (2385)	BB (3236)	BB (3236)	BB (3236)	BB (3290)	BB (2800)
rs17107057	52588298		AA (2385)	AA (3236)	AA (3236)	AA (3236)	-3290	AA (2800)
rs553648	52619890		BB (2385)	BB (3236)	BB (3236)	BB (3236)	BB (3290)	BB (2800)
rs10888749	52709480		BB (2385)	BB (3236)	BB (3236)	BB (3236)	BB (3290)	BB (2800)
rs17364143	52745680		AA (2385)	AA (3236)	AA (3236)	AA (3236)	AA (3290)	AA (2800)
rs2820331	52764959		AA (2385)	AA (3236)	AA (3236)	AA (3236)	AA (3290)	AA (2800)
rs2753402	52771408		BB (2385)	BB (3236)	BB (3236)	BB (3236)	BB (3290)	BB (2800)
rs11205950	52794842		BB (2385)	BB (3236)	BB (3236)	BB (3236)	BB (3290)	BB (2800)
rs2762819	52798033		BB (2385)	BB (3236)	BB (3236)	BB (3236)	BB (3290)	BB (2800)
rs2820337	52801061		AA (2385)	AA (3236)	AA (3236)	AA (3236)	AA (3290)	AA (2800)
rs7517338	52811581		BB (2385)	BB (3236)	BB (3236)	BB (3236)	BB (3290)	BB (2800)
rs9633423	52836139		AA (2385)	AA (3236)	AA (3236)	AA (3236)	AA (3290)	AA (2800)

rs10157619	52836736		BB (2385)	BB (3236)	BB (3236)	BB (3236)	BB (3290)	BB (2800)
rs6672059	52855047		BB (2385)	BB (3236)	BB (3236)	BB (3236)	BB (3290)	BB (2800)
rs3087477	52861871		AA (2385)	AA (3236)	AA (3236)	AA (3236)	AA (3290)	AA (2800)
rs6665054	52874114		AA (2385)	AA (3236)	AA (3236)	AA (3236)	AA (3290)	AA (2800)
rs10127490	52874787		BB (2385)	BB (3236)	BB (3236)	BB (3236)	BB (3290)	BB (2800)
rs17373044	52887953		BB (2385)	BB (3236)	BB (3236)	BB (3236)	BB (3290)	BB (2800)
rs10493166	52897395		BB (2385)	BB (3236)	BB (3236)	BB (3236)	BB (3290)	BB (2800)
rs12239281	52915793		BB (2385)	BB (3236)	BB (3236)	BB (3236)	BB (3290)	BB (2800)
rs7554003	52916371		BB (2385)	BB (3236)	BB (3236)	BB (3236)	BB (3290)	BB (2800)
rs13376564	52928448		BB (2385)	BB (3236)	BB (3236)	BB (3236)	BB (3290)	BB (2800)
rs6588429	52972639		BB (2385)	BB (3236)	BB (3236)	BB (3236)	BB (3290)	BB (2800)
rs997915	52973210		AA (2385)	AA (3236)	AA (3236)	AA (3236)	AA (3290)	AA (2800)
rs7553627	52986978		AA (2385)	AA (3236)	AA (3236)	AA (3236)	AA (3290)	AA (2800)
rs6588430	53021961		BB (2385)	BB (3236)	BB (3236)	BB (3236)	BB (3290)	BB (2800)
rs17107307	53039246		AA (2385)	AA (3236)	AA (3236)	AA (3236)	AA (3290)	AA (2800)
rs17373814	53039603		BB (2385)	BB (3236)	BB (3236)	BB (3236)	BB (3290)	BB (2800)
rs835353	53049831		BB (2385)	BB (3236)	BB (3236)	BB (3236)	BB (3290)	BB (2800)
rs1097233	53063087		BB (2385)	BB (3236)	BB (3236)	BB (3236)	BB (3290)	BB (2800)
rs835340	53063913		AA (2385)	AA (3236)	AA (3236)	AA (3236)	AA (3290)	AA (2800)
rs835341	53064012		BB (2385)	BB (3236)	BB (3236)	BB (3236)	BB (3290)	BB (2800)
rs835342	53064034		BB (2385)	BB (3236)	BB (3236)	BB (3236)	BB (3290)	BB (2800)
rs6682886	53079523		BB (2385)	BB (3236)	BB (3236)	BB (3236)	BB (3290)	BB (2800)
rs6671552	53081585		BB (2385)	BB (3236)	BB (3236)	BB (3236)	BB (3290)	BB (2800)
rs6688451	53086040		AA (2385)	AA (3236)	AA (3236)	AA (3236)	AA (3290)	AA (2800)
rs2050696	53094802		AA (2385)	AA (3236)	AA (3236)	AA (3236)	AA (3290)	AA (2800)
rs494030	53102527		BB (2385)	BB (3236)	BB (3236)	BB (3236)	BB (3290)	BB (2800)
rs2485917	53112410		BB (2385)	BB (3236)	BB (3236)	BB (3236)	BB (3290)	BB (2800)
rs706497	53114736		AA (2385)	AA (3236)	AA (3236)	AA (3236)	AA (3290)	AA (2800)
rs269324	53126155		AA (2385)	AA (3236)	AA (3236)	AA (3236)	AA (3290)	AA (2800)
rs6672879	53137112		BB (2385)	BB (3236)	BB (3236)	BB (3236)	BB (3290)	BB (2800)
rs12067454	53137328		BB (2385)	BB (3236)	BB (3236)	BB (3236)	BB (3290)	BB (2800)
rs448788	53161620		AA (2385)	AA (3236)	AA (3236)	AA (3236)	AA (3290)	AA (2800)
rs269286	53169018		AA (2385)	AA (3236)	AA (3236)	AA (3236)	AA (3290)	AA (2800)
rs440871	53173052		BB (2385)	BB (3236)	BB (3236)	BB (3236)	BB (3290)	BB (2800)
rs387436	53179376		AA (2385)	AA (3236)	AA (3236)	AA (3236)	AA (3290)	AA (2800)
rs425520	53179805		BB (2385)	BB (3236)	BB (3236)	BB (3236)	BB (3290)	BB (2800)
rs6703127	53180565		BB (2385)	BB (3236)	BB (3236)	BB (3236)	BB (3290)	BB (2800)
rs6703281	53180716		BB (2385)	BB (3236)	BB (3236)	BB (3236)	BB (3290)	BB (2800)
rs146750	53183447		BB (2385)	BB (3236)	BB (3236)	BB (3236)	BB (3290)	BB (2800)
rs12095247	53193796		AA (2385)	AA (3236)	AA (3236)	AA (3236)	AA (3290)	AA (2800)
rs6661196	53203664		AA (2385)	AA (3236)	AA (3236)	AA (3236)	AA (3290)	AA (2800)
rs6588439	53209442		BB (2385)	BB (3236)	BB (3236)	BB (3236)	BB (3290)	BB (2800)
rs4926928	53210163		BB (2385)	BB (3236)	BB (3236)	BB (3236)	BB (3290)	BB (2800)
rs10157120	53210888		BB (2385)	BB (3236)	BB (3236)	BB (3236)	BB (3290)	BB (2800)
rs10158562	53211003		BB (2385)	BB (3236)	BB (3236)	BB (3236)	BB (3290)	BB (2800)
rs12038438	53230581		AA (2385)	AA (3236)	AA (3236)	AA (3236)	AA (3290)	AA (2800)
rs10888755	53240829		AA (2385)	AA (3236)	AA (3236)	AA (3236)	AA (3290)	AA (2800)
rs2065477	53250213		BB (2385)	BB (3236)	BB (3236)	BB (3236)	BB (3290)	BB (2800)
rs12080613	53257411		AA (2385)	AA (3236)	AA (3236)	AA (3236)	AA (3290)	AA (2800)
rs12731800	53286967		BB (2385)	BB (3236)	BB (3236)	BB (3236)	BB (3290)	BB (2800)

rs480299	53320274	BB (2385)	BB (3236)	BB (3236)	BB (3236)	BB (3290)	BB (2800)
rs554301	53321948	AA (2385)	AA (3236)	AA (3236)	AA (3236)	AA (3290)	AA (2800)
rs514881	53336737	AA (2385)	AA (3236)	AA (3236)	AA (3236)	AA (3290)	AA (2800)
rs512723	53343880	AA (2385)	AA (3236)	AA (3236)	AA (3236)	AA (3290)	AA (2800)
rs499195	53344907	BB (2385)	BB (3236)	BB (3236)	BB (3236)	BB (3290)	BB (2800)
rs1044365	53361928	AA (2385)	AA (3236)	AA (3236)	AA (3236)	AA (3290)	AA (2800)
rs12094972	53385292	BB (2385)	BB (3236)	BB (3236)	BB (3236)	BB (3290)	BB (2800)
rs17107484	53410388	AA (2385)	AA (3236)	AA (3236)	AA (3236)	AA (3290)	AA (2800)
rs11206047	53412213	BB (2385)	BB (3236)	BB (3236)	BB (3236)	BB (3290)	BB (2800)
rs12060111	53412769	BB (2385)	BB (3236)	BB (3236)	BB (3236)	BB (3290)	BB (2800)
rs7528867	53453930	BB (2385)	BB (3236)	BB (3236)	BB (3236)	BB (3290)	BB (2800)
rs11206061	53455899	AA (2385)	AA (3236)	AA (3236)	AA (3236)	AA (3290)	AA (2800)
rs6697076	53459362	AA (2385)	AA (3236)	AA (3236)	AA (3236)	AA (3290)	AA (2800)
rs11581638	53459690	BB (2385)	BB (3236)	BB (3236)	BB (3236)	BB (3290)	BB (2800)
rs17107541	53462338	AA (2385)	AA (3236)	AA (3236)	AA (3236)	AA (3290)	AA (2800)
rs6679819	53476970	BB (2385)	BB (3236)	BB (3236)	BB (3236)	BB (3290)	BB (2800)
rs7529265	53480745	AA (2385)	AA (3236)	AA (3236)	AA (3236)	AA (3290)	AA (2800)
rs7546714	53488564	AA (2385)	AA (3236)	AA (3236)	AA (3236)	AA (3290)	AA (2800)
rs7555050	53489132	BB (2385)	BB (3236)	BB (3236)	BB (3236)	BB (3290)	BB (2800)
rs7549603	53489296	BB (2385)	BB (3236)	BB (3236)	BB (3236)	BB (3290)	BB (2800)
rs17107654	53494879	AA (2385)	AA (3236)	AA (3236)	AA (3236)	AA (3290)	AA (2800)
rs13376501	53512285	BB (2385)	BB (3236)	BB (3236)	BB (3236)	BB (3290)	BB (2800)
rs11588200	53512519	AA (2385)	AA (3236)	AA (3236)	AA (3236)	AA (3290)	AA (2800)
rs6664845	53514497	BB (2385)	BB (3236)	BB (3236)	BB (3236)	BB (3290)	BB (2800)
rs3766762	53514516	AA (2385)	AA (3236)	AA (3236)	AA (3236)	AA (3290)	AA (2800)
rs4926946	53515101	BB (2385)	BB (3236)	BB (3236)	BB (3236)	BB (3290)	BB (2800)
rs17107767	53517997	BB (2385)	BB (3236)	BB (3236)	BB (3236)	BB (3290)	BB (2800)
rs12096165	53518049	BB (2385)	BB (3236)	BB (3236)	BB (3236)	BB (3290)	BB (2800)
rs17107792	53530023	BB (2385)	BB (3236)	BB (3236)	BB (3236)	BB (3290)	BB (2800)
rs11584649	53530487	BB (2385)	BB (3236)	BB (3236)	BB (3236)	BB (3290)	BB (2800)
rs11580093	53532609	AA (2385)	AA (3236)	AA (3236)	AA (3236)	AA (3290)	AA (2800)
rs17107812	53532920	BB (2385)	BB (3236)	BB (3236)	BB (3236)	BB (3290)	BB (2800)
rs6697308	53534687	BB (2385)	BB (3236)	BB (3236)	BB (3236)	BB (3290)	BB (2800)
rs12568971	53537833	BB (2385)	BB (3236)	BB (3236)	BB (3236)	BB (3290)	BB (2800)
rs2306459	53554305	BB (2385)	BB (3236)	BB (3236)	BB (3236)	BB (3290)	BB (2800)
rs11583585	53554406	AA (2385)	AA (3236)	AA (3236)	AA (3236)	AA (3290)	AA (2800)
rs1288418	53564586	AA (2385)	AA (3236)	AA (3236)	AA (3236)	AA (3290)	AA (2800)
rs4926953	53569492	AA (2385)	AA (3236)	AA (3236)	AA (3236)	AA (3290)	AA (2800)
rs11206101	53570659	BB (2385)	BB (3236)	BB (3236)	BB (3236)	BB (3290)	BB (2800)
rs3766779	53578174	AA (2385)	AA (3236)	AA (3236)	AA (3236)	AA (3290)	AA (2800)
rs1288384	53583171	AA (2385)	AA (3236)	AA (3236)	AA (3236)	AA (3290)	AA (2800)
rs6694834	53587179	BB (2385)	BB (3236)	BB (3236)	BB (3236)	BB (3290)	BB (2800)
rs1288356	53599033	BB (2385)	BB (3236)	BB (3236)	BB (3236)	BB (3290)	BB (2800)
rs1288354	53600350	BB (2385)	BB (3236)	BB (3236)	BB (3236)	-3290	BB (2800)
rs1288367	53605884	AA (2385)	AA (3236)	AA (3236)	AA (3236)	AA (3290)	AA (2800)
rs11206109	53606080	BB (2385)	BB (3236)	BB (3236)	BB (3236)	BB (3290)	BB (2800)
rs34418279	53608425	BB (2385)	BB (3236)	BB (3236)	BB (3236)	BB (3290)	BB (2800)
rs10888768	53615541	AA (2385)	AA (3236)	AA (3236)	AA (3236)	AA (3290)	AA (2800)
rs1481043	53619502	BB (2385)	BB (3236)	BB (3236)	BB (3236)	BB (3290)	BB (2800)
rs6689888	53620833	BB (2385)	BB (3236)	BB (3236)	BB (3236)	BB (3290)	BB (2800)

rs6588466	53625449		BB (2385)	BB (3236)	BB (3236)	BB (3236)	BB (3290)	BB (2800)
rs6682324	53626014		BB (2385)	BB (3236)	BB (3236)	BB (3236)	BB (3290)	BB (2800)
rs1288340	53628061		BB (2385)	BB (3236)	BB (3236)	BB (3236)	BB (3290)	BB (2800)
rs1288333	53630914		BB (2385)	BB (3236)	BB (3236)	BB (3236)	BB (3290)	BB (2800)
rs1288332	53630960		BB (2385)	BB (3236)	BB (3236)	BB (3236)	BB (3290)	BB (2800)
rs2046140	53632424		BB (2385)	BB (3236)	BB (3236)	BB (3236)	BB (3290)	BB (2800)
rs4543741	53636177		BB (2385)	BB (3236)	BB (3236)	BB (3236)	BB (3290)	BB (2800)
rs3125253	53637106		AA (2385)	AA (3236)	AA (3236)	AA (3236)	AA (3290)	AA (2800)
rs7531337	53641911		AA (2385)	AA (3236)	AA (3236)	AA (3236)	AA (3290)	AA (2800)
rs3102047	53642449		BB (2385)	BB (3236)	BB (3236)	BB (3236)	BB (3290)	BB (2800)
rs6704459	53643199		BB (2385)	BB (3236)	BB (3236)	BB (3236)	BB (3290)	BB (2800)
rs6588471	53644967		BB (2385)	BB (3236)	BB (3236)	BB (3236)	BB (3290)	BB (2800)
rs5177	53711735		AA (2385)	AA (3236)	AA (3236)	AA (3236)	AA (3290)	AA (2800)
rs11206127	53713549		BB (2385)	BB (3236)	BB (3236)	BB (3236)	BB (3290)	BB (2800)
rs869987	53723190		BB (2385)	BB (3236)	BB (3236)	BB (3236)	BB (3290)	BB (2800)
rs869988	53723349		BB (2385)	BB (3236)	BB (3236)	BB (3236)	BB (3290)	BB (2800)
rs17108177	53732138		BB (2385)	BB (3236)	BB (3236)	BB (3236)	BB (3290)	BB (2800)
rs2297657	53746595		BB (2385)	BB (3236)	BB (3236)	BB (3236)	BB (3290)	BB (2800)
rs10493173	53771202		BB (2385)	BB (3236)	BB (3236)	BB (3236)	BB (3290)	BB (2800)
rs1288489	53772544		AA (2385)	AA (3236)	AA (3236)	AA (3236)	AA (3290)	AA (2800)
rs884586	53782093		AA (2385)	AA (3236)	AA (3236)	AA (3236)	AA (3290)	AA (2800)
rs41360049	53788279		BB (2385)	BB (3236)	BB (3236)	BB (3236)	BB (3290)	BB (2800)
rs1288523	53788874		AA (2385)	AA (3236)	AA (3236)	AA (3236)	AA (3290)	AA (2800)
rs12354192	53796783		BB (2385)	BB (3236)	BB (3236)	BB (3236)	BB (3290)	BB (2800)
rs2225725	53796901		BB (2385)	BB (3236)	BB (3236)	BB (3236)	BB (3290)	BB (2800)
rs2782495	53808358		AA (2385)	AA (3236)	AA (3236)	AA (3236)	AA (3290)	AA (2800)
rs1416096	53808658		AA (2385)	AA (3236)	AA (3236)	AA (3236)	AA (3290)	AA (2800)
rs1416095	53808693		BB (2385)	BB (3236)	BB (3236)	BB (3236)	BB (3290)	BB (2800)
rs2782497	53809030		AA (2385)	AA (3236)	AA (3236)	AA (3236)	AA (3290)	AA (2800)
rs6698595	53811942		BB (2385)	BB (3236)	BB (3236)	BB (3236)	BB (3290)	BB (2800)
rs2027261	53819026		AA (2385)	AA (3236)	AA (3236)	AA (3236)	AA (3290)	AA (2800)
rs1999898	53829820		AA (2385)	AA (3236)	AA (3236)	AA (3236)	AA (3290)	AA (2800)
rs11206152	53831095		AA (2385)	AA (3236)	AA (3236)	AA (3236)	AA (3290)	AA (2800)
rs12041966	53832697		AA (2385)	AA (3236)	AA (3236)	AA (3236)	AA (3290)	AA (2800)
rs17108402	53846253		BB (2385)	BB (3236)	BB (3236)	BB (3236)	BB (3290)	BB (2800)
rs6660818	53857444		AA (2385)	AA (3236)	AA (3236)	AA (3236)	AA (3290)	AA (2800)
rs41447650	53860358		AA (2385)	AA (3236)	AA (3236)	AA (3236)	AA (3290)	AA (2800)
rs17371030	53864019		BB (2385)	BB (3236)	BB (3236)	BB (3236)	BB (3290)	BB (2800)
rs4926992	53870466		AA (2385)	AA (3236)	AA (3236)	AA (3236)	AA (3290)	AA (2800)
rs1288587	53873555		BB (2385)	BB (3236)	BB (3236)	BB (3236)	BB (3290)	BB (2800)
rs953625	53876381		AA (2385)	AA (3236)	AA (3236)	AA (3236)	AA (3290)	AA (2800)
rs17379805	53877480		AA (2385)	AA (3236)	AA (3236)	AA (3236)	AA (3290)	AA (2800)
rs17108507	53879927		BB (2385)	BB (3236)	BB (3236)	BB (3236)	BB (3290)	BB (2800)
rs17108515	53880356		AA (2385)	AA (3236)	AA (3236)	AA (3236)	AA (3290)	AA (2800)
rs6685296	53880369		AA (2385)	AA (3236)	AA (3236)	AA (3236)	AA (3290)	AA (2800)
rs6683597	53881254		BB (2385)	BB (3236)	BB (3236)	BB (3236)	BB (3290)	BB (2800)
rs6697300	53881608		BB (2385)	BB (3236)	BB (3236)	BB (3236)	BB (3290)	BB (2800)
rs12033075	53897517		AA (2385)	AA (3236)	AA (3236)	AA (3236)	AA (3290)	AA (2800)
rs7512516	53909020		BB (2385)	BB (3236)	BB (3236)	BB (3236)	BB (3290)	BB (2800)
rs11206160	53911812		BB (2385)	BB (3236)	BB (3236)	BB (3236)	BB (3290)	BB (2800)

rs12046664	53916576		AA (2385)	AA (3236)	AA (3236)	AA (3236)	AA (3290)	AA (2800)
rs41366756	53917443		BB (2385)	BB (3236)	BB (3236)	BB (3236)	BB (3290)	BB (2800)
rs41421650	53918872		BB (2385)	BB (3236)	BB (3236)	BB (3236)	BB (3290)	BB (2800)
rs41443646	53921571		BB (2385)	BB (3236)	BB (3236)	BB (3236)	BB (3290)	BB (2800)
rs7543581	53922155		AA (2385)	AA (3236)	AA (3236)	AA (3236)	AA (3290)	AA (2800)
rs1108989	53929463		AA (2385)	AA (3236)	AA (3236)	AA (3236)	AA (3290)	AA (2800)
rs1288632	53929536		BB (2385)	BB (3236)	BB (3236)	BB (3236)	BB (3290)	BB (2800)
rs11579575	53936718		BB (2385)	BB (3236)	BB (3236)	BB (3236)	BB (3290)	BB (2800)
rs1288642	53940255		AA (2385)	AA (3236)	AA (3236)	AA (3236)	AA (3290)	AA (2800)
rs17108559	53947868		AA (2385)	AA (3236)	AA (3236)	AA (3236)	AA (3290)	AA (2800)
rs943520	53965176		BB (2385)	BB (3236)	BB (3236)	BB (3236)	BB (3290)	BB (2800)
rs7520584	54000726		AA (2385)	AA (3236)	AA (3236)	AA (3236)	AA (3290)	AA (2800)
rs9700109	54009376		BB (2385)	BB (3236)	BB (3236)	BB (3236)	BB (3290)	BB (2800)
rs17108709	54010826		AA (2385)	AA (3236)	AA (3236)	AA (3236)	AA (3290)	AA (2800)
rs6682171	54011142		BB (2385)	BB (3236)	BB (3236)	BB (3236)	BB (3290)	BB (2800)
rs41488848	54011503		AA (2385)	AA (3236)	AA (3236)	AA (3236)	AA (3290)	AA (2800)
rs17108762	54014413		BB (2385)	BB (3236)	BB (3236)	BB (3236)	BB (3290)	BB (2800)
rs563403	54014492		AA (2385)	AA (3236)	AA (3236)	AA (3236)	AA (3290)	AA (2800)
rs552789	54027909		BB (2385)	BB (3236)	BB (3236)	BB (3236)	BB (3290)	BB (2800)
rs10493174	54038536		AA (2385)	AA (3236)	AA (3236)	AA (3236)	AA (3290)	AA (2800)
rs4634849	54043110		AA (2385)	AA (3236)	AA (3236)	AA (3236)	AA (3290)	AA (2800)
rs17386087	54045624		BB (2385)	BB (3236)	BB (3236)	BB (3236)	BB (3290)	BB (2800)
rs17386108	54045718		BB (2385)	BB (3236)	BB (3236)	BB (3236)	BB (3290)	BB (2800)
rs7551844	54061333		AA (2385)	AA (3236)	AA (3236)	AA (3236)	AA (3290)	AA (2800)
rs10888795	54061446		AA (2385)	AA (3236)	AA (3236)	AA (3236)	AA (3290)	AA (2800)
rs3013754	54061978		BB (2385)	BB (3236)	BB (3236)	BB (3236)	BB (3290)	BB (2800)
rs2948053	54062273		AA (2385)	AA (3236)	AA (3236)	AA (3236)	AA (3290)	AA (2800)
rs1320385	54064469		BB (2385)	BB (3236)	BB (3236)	BB (3236)	BB (3290)	BB (2800)
rs12120383	54065523		AA (2385)	AA (3236)	AA (3236)	AA (3236)	AA (3290)	AA (2800)
rs12036675	54066318		BB (2385)	BB (3236)	BB (3236)	BB (3236)	BB (3290)	BB (2800)
rs12062528	54070831		BB (2385)	BB (3236)	BB (3236)	BB (3236)	BB (3290)	BB (2800)
rs17382457	54071428		AA (2385)	AA (3236)	AA (3236)	AA (3236)	AA (3290)	AA (2800)
rs12563871	54071610		AA (2385)	AA (3236)	AA (3236)	AA (3236)	AA (3290)	AA (2800)
rs3013749	54072759		AA (2385)	AA (3236)	AA (3236)	AA (3236)	AA (3290)	AA (2800)
rs3013747	54074803		AA (2385)	AA (3236)	AA (3236)	AA (3236)	AA (3290)	AA (2800)
rs3006894	54076357		AA (2385)	AA (3236)	AA (3236)	AA (3236)	AA (3290)	AA (2800)
rs2948055	54079808		AA (2385)	AA (3236)	AA (3236)	AA (3236)	AA (3290)	AA (2800)
rs11801290	54079964		BB (2385)	BB (3236)	BB (3236)	BB (3236)	BB (3290)	BB (2800)
rs4927016	54080244		BB (2385)	BB (3236)	BB (3236)	BB (3236)	BB (3290)	BB (2800)
rs4927017	54080347		BB (2385)	BB (3236)	BB (3236)	BB (3236)	BB (3290)	BB (2800)
rs3006897	54083213		AA (2385)	AA (3236)	AA (3236)	AA (3236)	AA (3290)	AA (2800)
rs3006900	54086201		AA (2385)	AA (3236)	AA (3236)	AA (3236)	AA (3290)	AA (2800)
rs12045240	54103375		AA (2385)	AA (3236)	AA (3236)	AA (3236)	AA (3290)	AA (2800)
rs10888802	54108906		AA (2385)	AA (3236)	AA (3236)	AA (3236)	AA (3290)	AA (2800)
rs11206186	54117985		AA (2385)	AA (3236)	AA (3236)	AA (3236)	AA (3290)	AA (2800)
rs7522391	54118849		BB (2385)	BB (3236)	BB (3236)	BB (3236)	BB (3290)	BB (2800)
rs12733756	54120307		BB (2385)	BB (3236)	BB (3236)	BB (3236)	BB (3290)	BB (2800)
rs11590616	54120838		AA (2385)	AA (3236)	AA (3236)	AA (3236)	AA (3290)	AA (2800)
rs2141079	54120873		BB (2385)	BB (3236)	BB (3236)	BB (3236)	BB (3290)	BB (2800)
rs2950241	54129591		BB (2385)	BB (3236)	BB (3236)	BB (3236)	BB (3290)	BB (2800)

rs17109076	54129705	AA (2385)	AA (3236)	AA (3236)	AA (3236)	AA (3290)	AA (2800)
rs2950242	54133294	AA (2385)	AA (3236)	AA (3236)	AA (3236)	AA (3290)	AA (2800)
rs2950243	54135651	BB (2385)	BB (3236)	BB (3236)	BB (3236)	BB (3290)	BB (2800)
rs12564271	54136057	AA (2385)	AA (3236)	AA (3236)	AA (3236)	AA (3290)	AA (2800)
rs11206191	54158102	BB (2385)	BB (3236)	BB (3236)	BB (3236)	BB (3290)	BB (2800)
rs1173586	54171212	AA (2385)	AA (3236)	AA (3236)	AA (3236)	AA (3290)	AA (2800)
rs1173589	54172944	BB (2385)	BB (3236)	BB (3236)	BB (3236)	BB (3290)	BB (2800)
rs1780391	54177708	AA (2385)	AA (3236)	AA (3236)	AA (3236)	AA (3290)	AA (2800)
rs11206205	54177891	AA (2385)	AA (3236)	AA (3236)	AA (3236)	AA (3290)	AA (2800)
rs11206210	54185389	BB (2385)	BB (3236)	BB (3236)	BB (3236)	BB (3290)	BB (2800)
rs11206211	54188096	BB (2385)	BB (3236)	BB (3236)	BB (3236)	BB (3290)	BB (2800)
rs12043385	54189668	AA (2385)	AA (3236)	AA (3236)	AA (3236)	AA (3290)	AA (2800)
rs7547962	54190045	AA (2385)	AA (3236)	AA (3236)	AA (3236)	AA (3290)	AA (2800)
rs11206212	54193922	BB (2385)	BB (3236)	BB (3236)	BB (3236)	BB (3290)	BB (2800)
rs808860	54196929	AA (2385)	AA (3236)	AA (3236)	AA (3236)	AA (3290)	AA (2800)
rs2982662	54203107	AA (2385)	AA (3236)	AA (3236)	AA (3236)	AA (3290)	AA (2800)
rs797896	54208835	BB (2385)	BB (3236)	BB (3236)	BB (3236)	BB (3290)	BB (2800)
rs6588489	54217646	AA (2385)	AA (3236)	AA (3236)	AA (3236)	AA (3290)	AA (2800)
rs1183394	54222571	AA (2385)	AA (3236)	AA (3236)	AA (3236)	AA (3290)	AA (2800)
rs1569783	54247271	AA (2385)	AA (3236)	AA (3236)	AA (3236)	AA (3290)	AA (2800)
rs1181183	54252198	AA (2385)	AA (3236)	AA (3236)	AA (3236)	AA (3290)	AA (2800)
rs17109377	54257376	BB (2385)	BB (3236)	BB (3236)	BB (3236)	BB (3290)	BB (2800)
rs1181177	54266678	AA (2385)	AA (3236)	AA (3236)	AA (3236)	AA (3290)	AA (2800)
rs1181156	54276225	AA (2385)	AA (3236)	AA (3236)	AA (3236)	AA (3290)	AA (2800)
rs1181189	54289215	BB (2385)	BB (3236)	BB (3236)	BB (3236)	BB (3290)	BB (2800)
rs1181185	54292601	AA (2385)	AA (3236)	AA (3236)	AA (3236)	AA (3290)	AA (2800)
rs12070315	54315632	AA (2385)	AA (3236)	AA (3236)	AA (3236)	AA (3290)	AA (2800)
rs1127931	54317485	AA (2385)	AA (3236)	AA (3236)	AA (3236)	AA (3290)	AA (2800)
rs11206227	54323368	AA (2385)	AA (3236)	AA (3236)	AA (3236)	AA (3290)	AA (2800)
rs12022704	54323891	BB (2385)	BB (3236)	BB (3236)	BB (3236)	BB (3290)	BB (2800)
rs6588492	54328025	BB (2385)	BB (3236)	BB (3236)	BB (3236)	BB (3290)	BB (2800)
rs6681549	54328814	BB (2385)	BB (3236)	BB (3236)	BB (3236)	BB (3290)	BB (2800)
rs6588493	54328988	BB (2385)	BB (3236)	BB (3236)	BB (3236)	BB (3290)	BB (2800)
rs7541049	54343294	BB (2385)	BB (3236)	BB (3236)	BB (3236)	BB (3290)	BB (2800)
rs7541170	54343462	BB (2385)	BB (3236)	BB (3236)	BB (3236)	BB (3290)	BB (2800)
rs6680026	54345792	BB (2385)	BB (3236)	BB (3236)	BB (3236)	BB (3290)	BB (2800)
rs6588496	54364809	BB (2385)	BB (3236)	BB (3236)	BB (3236)	BB (3290)	BB (2800)
rs10888818	54365679	AA (2385)	AA (3236)	AA (3236)	AA (3236)	AA (3290)	AA (2800)
rs731828	54368253	BB (2385)	BB (3236)	BB (3236)	BB (3236)	BB (3290)	BB (2800)
rs2294512	54370306	AA (2385)	AA (3236)	AA (3236)	AA (3236)	AA (3290)	AA (2800)
rs11206244	54375701	BB (2385)	BB (3236)	BB (3236)	BB (3236)	BB (3290)	BB (2800)
rs12063777	54381406	AA (2385)	AA (3236)	AA (3236)	AA (3236)	AA (3290)	AA (2800)
rs12072178	54392302	AA (2385)	AA (3236)	AA (3236)	AA (3236)	AA (3290)	AA (2800)
rs11206252	54397240	BB (2385)	BB (3236)	BB (3236)	BB (3236)	BB (3290)	BB (2800)
rs12092723	54422901	AA (2385)	AA (3236)	AA (3236)	AA (3236)	AA (3290)	AA (2800)
rs2294514	54432019	BB (2385)	BB (3236)	BB (3236)	BB (3236)	BB (3290)	BB (2800)
rs7515322	54450901	AA (2385)	AA (3236)	AA (3236)	AA (3236)	AA (3290)	AA (2800)
rs7525809	54451080	AA (2385)	AA (3236)	AA (3236)	AA (3236)	AA (3290)	AA (2800)
rs637590	54461703	BB (2385)	BB (3236)	BB (3236)	BB (3236)	BB (3290)	BB (2800)
rs17109648	54472067	BB (2385)	BB (3236)	BB (3236)	BB (3236)	BB (3290)	BB (2800)

rs928443	54473516	BB (2385)	BB (3236)	BB (3236)	BB (3236)	BB (3290)	BB (2800)
rs928444	54473548	BB (2385)	BB (3236)	BB (3236)	BB (3236)	BB (3290)	BB (2800)
rs17109656	54476147	AA (2385)	AA (3236)	AA (3236)	AA (3236)	AA (3290)	AA (2800)
rs1537323	54482523	BB (2385)	BB (3236)	BB (3236)	BB (3236)	BB (3290)	BB (2800)
rs1999490	54485585	BB (2385)	BB (3236)	BB (3236)	BB (3236)	BB (3290)	BB (2800)
rs12085915	54495254	AA (2385)	AA (3236)	AA (3236)	AA (3236)	AA (3290)	AA (2800)
rs7539402	54500487	AA (2385)	AA (3236)	AA (3236)	AA (3236)	AA (3290)	AA (2800)
rs41331348	54505756	AA (2385)	AA (3236)	AA (3236)	AA (3236)	AA (3290)	AA (2800)
rs41538044	54511200	AA (2385)	AA (3236)	AA (3236)	AA (3236)	AA (3290)	AA (2800)
rs11206279	54519158	AA (2385)	AA (3236)	AA (3236)	AA (3236)	AA (3290)	AA (2800)
rs6694292	54566190	BB (2385)	BB (3236)	BB (3236)	BB (3236)	BB (3290)	BB (2800)
rs6694397	54566310	AA (2385)	AA (3236)	AA (3236)	AA (3236)	AA (3290)	AA (2800)
rs9919295	54578100	AA (2385)	AA (3236)	AA (3236)	AA (3236)	AA (3290)	AA (2800)
rs1329780	54580012	AA (2385)	AA (3236)	AA (3236)	AA (3236)	AA (3290)	AA (2800)
rs17109806	54587121	BB (2385)	BB (3236)	BB (3236)	BB (3236)	BB (3290)	BB (2800)
rs7528864	54588882	BB (2385)	BB (3236)	BB (3236)	BB (3236)	BB (3290)	BB (2800)
rs4483354	54598713	BB (2385)	BB (3236)	BB (3236)	BB (3236)	BB (3290)	BB (2800)
rs3766465	54606804	AA (2385)	AA (3236)	AA (3236)	AA (3236)	AA (3290)	AA (2800)
rs671539	54607157	BB (2385)	BB (3236)	BB (3236)	BB (3236)	BB (3290)	BB (2800)
rs6696001	54614145	AA (2385)	AA (3236)	AA (3236)	AA (3236)	AA (3290)	AA (2800)
rs581554	54614409	BB (2385)	BB (3236)	BB (3236)	BB (3236)	BB (3290)	BB (2800)
rs6667290	54616538	AA (2385)	AA (3236)	AA (3236)	AA (3236)	AA (3290)	AA (2800)
rs1999175	54616753	BB (2385)	BB (3236)	BB (3236)	BB (3236)	BB (3290)	BB (2800)
rs9436480	54617256	AA (2385)	AA (3236)	AA (3236)	AA (3236)	AA (3290)	AA (2800)
rs632961	54617716	BB (2385)	BB (3236)	BB (3236)	BB (3236)	BB (3290)	BB (2800)
rs660514	54625292	BB (2385)	BB (3236)	BB (3236)	BB (3236)	BB (3290)	BB (2800)
rs17109968	54633543	AA (2385)	AA (3236)	AA (3236)	AA (3236)	AA (3290)	AA (2800)
rs17109974	54633630	AA (2385)	AA (3236)	AA (3236)	AA (3236)	AA (3290)	AA (2800)
rs7528869	54645313	AA (2385)	AA (3236)	AA (3236)	AA (3236)	AA (3290)	AA (2800)
rs1890566	54668278	BB (2385)	BB (3236)	BB (3236)	BB (3236)	BB (3290)	BB (2800)
rs17110087	54668562	AA (2385)	AA (3236)	AA (3236)	AA (3236)	AA (3290)	AA (2800)
rs11206304	54679319	AA (2385)	AA (3236)	AA (3236)	AA (3236)	AA (3290)	AA (2800)
rs4927071	54679912	BB (2385)	BB (3236)	BB (3236)	BB (3236)	BB (3290)	BB (2800)
rs10888841	54687065	BB (2385)	BB (3236)	BB (3236)	BB (3236)	BB (3290)	BB (2800)
rs3766453	54702869	BB (2385)	BB (3236)	BB (3236)	BB (3236)	BB (3290)	BB (2800)
rs3766448	54703768	BB (2385)	BB (3236)	BB (3236)	BB (3236)	BB (3290)	BB (2800)
rs3766430	54730651	BB (2385)	BB (3236)	BB (3236)	BB (3236)	BB (3290)	BB (2800)
rs603901	54741767	AA (2385)	AA (3236)	AA (3236)	AA (3236)	AA (3290)	AA (2800)
rs9326028	54750625	BB (2385)	BB (3236)	BB (3236)	BB (3236)	BB (3290)	BB (2800)
rs10788977	54751900	BB (2385)	BB (3236)	BB (3236)	BB (3236)	BB (3290)	BB (2800)
rs623888	54754848	AA (2385)	AA (3236)	AA (3236)	AA (3236)	AA (3290)	AA (2800)
rs17110262	54756080	AA (2385)	AA (3236)	AA (3236)	AA (3236)	AA (3290)	AA (2800)
rs10489809	54757401	BB (2385)	BB (3236)	BB (3236)	BB (3236)	BB (3290)	BB (2800)
rs17392743	54764241	BB (2385)	BB (3236)	BB (3236)	BB (3236)	BB (3290)	BB (2800)
rs4927086	54766101	BB (2385)	BB (3236)	BB (3236)	BB (3236)	BB (3290)	BB (2800)
rs4927088	54775203	AA (2385)	AA (3236)	AA (3236)	AA (3236)	AA (3290)	AA (2800)
rs11206329	54777562	BB (2385)	BB (3236)	BB (3236)	BB (3236)	BB (3290)	BB (2800)
rs12120218	54787409	AA (2385)	AA (3236)	AA (3236)	AA (3236)	AA (3290)	AA (2800)
rs4927089	54788928	BB (2385)	BB (3236)	BB (3236)	BB (3236)	BB (3290)	BB (2800)
rs12404407	54792874	AA (2385)	AA (3236)	AA (3236)	AA (3236)	AA (3290)	AA (2800)

rs12563481	54793683		BB (2385)	BB (3236)	BB (3236)	BB (3236)	BB (3290)	BB (2800)
rs3901222	54808435		AA (2385)	AA (3236)	AA (3236)	AA (3236)	AA (3290)	AA (2800)
rs12092071	54810970		BB (2385)	BB (3236)	BB (3236)	BB (3236)	BB (3290)	BB (2800)
rs4601533	54819977		AA (2385)	AA (3236)	AA (3236)	AA (3236)	AA (3290)	AA (2800)
rs2073109	54827554		BB (2385)	BB (3236)	BB (3236)	BB (3236)	BB (3290)	BB (2800)
rs2073108	54839069		AA (2385)	AA (3236)	AA (3236)	AA (3236)	AA (3290)	AA (2800)
rs17101278	54840496		BB (2385)	BB (3236)	BB (3236)	BB (3236)	BB (3290)	BB (2800)
rs6690450	54854079		AA (2385)	AA (3236)	AA (3236)	AA (3236)	AA (3290)	AA (2800)
rs213490	54854984		BB (2385)	BB (3236)	BB (3236)	BB (3236)	BB (3290)	BB (2800)
rs213481	54861540		BB (2385)	BB (3236)	BB (3236)	BB (3236)	BB (3290)	BB (2800)
rs4927099	54882458		BB (2385)	BB (3236)	BB (3236)	BB (3236)	BB (3290)	BB (2800)
rs4244644	54883163		BB (2385)	BB (3236)	BB (3236)	BB (3236)	BB (3290)	BB (2800)
rs4927107	54888245		AA (2385)	AA (3236)	AA (3236)	AA (3236)	AA (3290)	AA (2800)
rs10888853	54919459		AA (2385)	AA (3236)	AA (3236)	AA (3236)	AA (3290)	AA (2800)
rs2873449	54959294		BB (2385)	BB (3236)	BB (3236)	BB (3236)	BB (3290)	BB (2800)
rs904218	54960989		AA (2385)	AA (3236)	AA (3236)	AA (3236)	AA (3290)	AA (2800)
rs10888854	54961748		BB (2385)	BB (3236)	BB (3236)	BB (3236)	BB (3290)	BB (2800)
rs904219	54962365		AA (2385)	AA (3236)	AA (3236)	AA (3236)	AA (3290)	AA (2800)
rs299895	54962448		AA (2385)	AA (3236)	AA (3236)	AA (3236)	AA (3290)	AA (2800)
rs11206370	54963958		AA (2385)	AA (3236)	AA (3236)	AA (3236)	AA (3290)	AA (2800)
rs7512248	54978948		BB (2385)	BB (3236)	BB (3236)	BB (3236)	BB (3290)	BB (2800)
rs4927136	54982705		BB (2385)	BB (3236)	BB (3236)	BB (3236)	BB (3290)	BB (2800)
rs2133003	55022557		BB (2385)	BB (3236)	BB (3236)	BB (3236)	BB (3290)	BB (2800)
rs537696	55031313		AA (2385)	AA (3236)	AA (3236)	AA (3236)	AA (3290)	AA (2800)
rs41503150	55036884		AA (2385)	AA (3236)	AA (3236)	AA (3236)	AA (3290)	AA (2800)
rs3851855	55038981		BB (2385)	BB (3236)	BB (3236)	BB (3236)	BB (3290)	BB (2800)
rs473806	55042325		BB (2385)	BB (3236)	BB (3236)	BB (3236)	BB (3290)	BB (2800)
rs477366	55042678		BB (2385)	BB (3236)	BB (3236)	BB (3236)	BB (3290)	BB (2800)
rs478225	55042757		BB (2385)	BB (3236)	BB (3236)	BB (3236)	BB (3290)	BB (2800)
rs300271	55046039		BB (2385)	BB (3236)	BB (3236)	BB (3236)	BB (3290)	BB (2800)
rs11811633	55046130		BB (2385)	BB (3236)	BB (3236)	BB (3236)	BB (3290)	BB (2800)
rs7521400	55047801		AA (2385)	AA (3236)	AA (3236)	AA (3236)	AA (3290)	AA (2800)
rs17110749	55048190		AA (2385)	AA (3236)	AA (3236)	AA (3236)	AA (3290)	AA (2800)
rs41351245	55050813		AA (2385)	AA (3236)	AA (3236)	AA (3236)	AA (3290)	AA (2800)
rs12564436	55057663		AA (2385)	AA (3236)	AA (3236)	AA (3236)	AA (3290)	AA (2800)
rs4927139	55061254		AA (2385)	AA (3236)	AA (3236)	AA (3236)	AA (3290)	AA (2800)
rs732509	55067992		AA (2385)	AA (3236)	AA (3236)	AA (3236)	AA (3290)	AA (2800)
rs2289015	55075062		AA (2385)	AA (3236)	AA (3236)	AA (3236)	AA (3290)	AA (2800)
rs10736390	55103945		BB (2385)	BB (3236)	BB (3236)	BB (3236)	BB (3290)	BB (2800)
rs11206403	55106933		BB (2385)	BB (3236)	BB (3236)	BB (3236)	BB (3290)	BB (2800)
rs10888858	55108978		BB (2385)	BB (3236)	BB (3236)	BB (3236)	BB (3290)	BB (2800)
rs17110873	55115117		AA (2385)	AA (3236)	AA (3236)	AA (3236)	AA (3290)	AA (2800)
rs1701999	55115608		AA (2385)	AA (3236)	AA (3236)	AA (3236)	AA (3290)	AA (2800)
rs1655519	55119515		BB (2385)	BB (3236)	BB (3236)	BB (3236)	BB (3290)	BB (2800)
rs1529783	55129564		BB (2385)	BB (3236)	BB (3236)	BB (3236)	BB (3290)	BB (2800)
rs689258	55129742		AA (2385)	AA (3236)	AA (3236)	AA (3236)	AA (3290)	AA (2800)
rs598130	55138294		BB (2385)	BB (3236)	BB (3236)	BB (3236)	BB (3290)	BB (2800)
rs571199	55139618		BB (2385)	BB (3236)	BB (3236)	BB (3236)	BB (3290)	BB (2800)
rs570218	55139741		BB (2385)	BB (3236)	BB (3236)	BB (3236)	BB (3290)	BB (2800)
rs498831	55144100		BB (2385)	BB (3236)	BB (3236)	BB (3236)	BB (3290)	BB (2800)

rs744748	55149254		AA (2385)	AA (3236)	AA (3236)	AA (3236)	AA (3290)	AA (2800)
rs3765018	55168298		BB (2385)	BB (3236)	BB (3236)	BB (3236)	BB (3290)	BB (2800)
rs3765017	55168394		BB (2385)	BB (3236)	BB (3236)	BB (3236)	BB (3290)	BB (2800)
rs1749859	55179005		BB (2385)	BB (3236)	BB (3236)	BB (3236)	BB (3290)	BB (2800)
rs1147990	55182300		AB (2385)	AB (3236)	AB (3236)	AB (3236)	AB (3290)	AB (2800)
rs1180973	55186662		AA (2385)	AA (3236)	AA (3236)	AA (3236)	AA (3290)	AA (2800)
rs10489663	55186686		AA (2385)	AA (3236)	AA (3236)	AA (3236)	AA (3290)	AA (2800)
rs1925653	55193872		AA (2385)	AA (3236)	AA (3236)	AA (3236)	AA (3290)	AA (2800)
rs41528549	55196739		BB (2385)	BB (3236)	BB (3236)	BB (3236)	BB (3290)	BB (2800)
rs3815226	55197149		BB (2385)	BB (3236)	BB (3236)	BB (3236)	BB (3290)	BB (2800)
rs13046	55207754		BB (2385)	BB (3236)	BB (3236)	BB (3236)	BB (3290)	BB (2800)
rs6698255	55212719		AA (2385)	AA (3236)	AA (3236)	AA (3236)	AA (3290)	AA (2800)
rs6683397	55212798		BB (2385)	BB (3236)	BB (3236)	BB (3236)	BB (3290)	BB (2800)
rs17515141	55217307		AA (2385)	AA (3236)	AA (3236)	AA (3236)	AA (3290)	AA (2800)
rs1180967	55217734		AA (2385)	AA (3236)	AA (3236)	AA (3236)	AA (3290)	AA (2800)
rs1180966	55217804		AA (2385)	AA (3236)	AA (3236)	AA (3236)	AA (3290)	AA (2800)
rs17111010	55217972		BB (2385)	BB (3236)	BB (3236)	BB (3236)	BB (3290)	BB (2800)
rs1180949	55228129		AA (2385)	AA (3236)	AA (3236)	AA (3236)	AA (3290)	AA (2800)
rs1180948	55228159		AA (2385)	AA (3236)	AA (3236)	AA (3236)	AA (3290)	AA (2800)
rs1180939	55234520		BB (2385)	BB (3236)	BB (3236)	BB (3236)	BB (3290)	BB (2800)
rs10430114	55234711		BB (2385)	BB (3236)	BB (3236)	BB (3236)	BB (3290)	BB (2800)
rs10888879	55243590		BB (2385)	BB (3236)	BB (3236)	BB (3236)	BB (3290)	BB (2800)
rs1180936	55255583		BB (2385)	BB (3236)	BB (3236)	BB (3236)	BB (3290)	BB (2800)
rs3753402	55260951		AA (2385)	AA (3236)	AA (3236)	AA (3236)	AA (3290)	AA (2800)
rs3753403	55261209		BB (2385)	BB (3236)	BB (3236)	BB (3236)	BB (3290)	BB (2800)
rs649398	55261390		AA (2385)	AA (3236)	AA (3236)	AA (3236)	AA (3290)	AA (2800)
rs4926658	55283076		BB (2385)	BB (3236)	BB (3236)	BB (3236)	BB (3290)	BB (2800)
rs4926659	55294722		AA (2385)	AA (3236)	AA (3236)	AA (3236)	AA (3290)	AA (2800)
rs6681687	55303439		BB (2385)	BB (3236)	BB (3236)	BB (3236)	BB (3290)	BB (2800)
rs12141358	55303782		AA (2385)	AA (3236)	AA (3236)	AA (3236)	AA (3290)	AA (2800)
rs17111092	55304723		BB (2385)	BB (3236)	BB (3236)	BB (3236)	BB (3290)	BB (2800)
rs17575462	55304836		BB (2385)	BB (3236)	BB (3236)	BB (3236)	BB (3290)	BB (2800)
rs10489659	55308609		AA (2385)	AA (3236)	AA (3236)	AA (3236)	AA (3290)	AA (2800)
rs593722	55312478		AA (2385)	AA (3236)	AA (3236)	AA (3236)	AA (3290)	AA (2800)
rs590621	55313161		BB (2385)	BB (3236)	BB (3236)	BB (3236)	BB (3290)	BB (2800)
rs594365	55313355		BB (2385)	BB (3236)	BB (3236)	BB (3236)	BB (3290)	BB (2800)
rs718265	55319902		AA (2385)	AA (3236)	AA (3236)	AA (3236)	AA (3290)	AA (2800)
rs11206455	55325794		BB (2385)	BB (3236)	BB (3236)	BB (3236)	BB (3290)	BB (2800)
rs4927168	55326345		BB (2385)	BB (3236)	BB (3236)	BB (3236)	BB (3290)	BB (2800)
rs11206457	55336097		BB (2385)	BB (3236)	BB (3236)	BB (3236)	BB (3290)	BB (2800)
rs608458	55336950		BB (2385)	BB (3236)	BB (3236)	BB (3236)	BB (3290)	BB (2800)
rs7551288	55338679		BB (2385)	BB (3236)	BB (3236)	BB (3236)	BB (3290)	BB (2800)
rs638944	55341915		BB (2385)	BB (3236)	BB (3236)	BB (3236)	BB (3290)	BB (2800)
rs589244	55342929		AA (2385)	AA (3236)	AA (3236)	AA (3236)	AA (3290)	AA (2800)
rs3170766	55354302		AA (2385)	AA (3236)	AA (3236)	AA (3236)	AA (3290)	AA (2800)
rs4927176	55354335		BB (2385)	BB (3236)	BB (3236)	BB (3236)	BB (3290)	BB (2800)
rs677321	55387362		AA (2385)	AA (3236)	AA (3236)	AA (3236)	AA (3290)	AA (2800)
rs678358	55387637		BB (2385)	BB (3236)	BB (3236)	BB (3236)	BB (3290)	BB (2800)
rs12141717	55388480		BB (2385)	BB (3236)	BB (3236)	BB (3236)	BB (3290)	BB (2800)
rs41517445	55389569		BB (2385)	BB (3236)	BB (3236)	BB (3236)	BB (3290)	BB (2800)

rs582679	55390841	AA (2385)	AA (3236)	AA (3236)	AA (3236)	AA (3290)	AA (2800)
rs35526112	55391268	BB (2385)	BB (3236)	BB (3236)	BB (3236)	BB (3290)	BB (2800)
rs2114580	55394648	AA (2385)	AA (3236)	AA (3236)	AA (3236)	AA (3290)	AA (2800)
rs11206471	55397616	BB (2385)	BB (3236)	BB (3236)	BB (3236)	BB (3290)	BB (2800)
rs11206480	55416827	AA (2385)	AA (3236)	AA (3236)	AA (3236)	AA (3290)	AA (2800)
rs7524689	55416974	BB (2385)	BB (3236)	BB (3236)	BB (3236)	BB (3290)	BB (2800)
rs7530321	55418248	BB (2385)	BB (3236)	BB (3236)	BB (3236)	BB (3290)	BB (2800)
rs7520033	55418263	BB (2385)	BB (3236)	BB (3236)	BB (3236)	BB (3290)	BB (2800)
rs6701789	55418976	AA (2385)	AA (3236)	AA (3236)	AA (3236)	AA (3290)	AA (2800)
rs11206482	55419855	BB (2385)	BB (3236)	BB (3236)	BB (3236)	BB (3290)	BB (2800)
rs7539163	55426441	BB (2385)	BB (3236)	BB (3236)	BB (3236)	BB (3290)	BB (2800)
rs7523658	55428397	AA (2385)	AA (3236)	AA (3236)	AA (3236)	AA (3290)	AA (2800)
rs11206497	55436790	BB (2385)	BB (3236)	BB (3236)	BB (3236)	BB (3290)	BB (2800)
rs17111402	55437512	AA (2385)	AA (3236)	AA (3236)	AA (3236)	AA (3290)	AA (2800)
rs11206499	55438925	AA (2385)	AA (3236)	AA (3236)	AA (3236)	AA (3290)	AA (2800)
rs2433673	55443605	AA (2385)	AA (3236)	AA (3236)	AA (3236)	AA (3290)	AA (2800)
rs1015979	55449273	AA (2385)	AA (3236)	AA (3236)	AA (3236)	AA (3290)	AA (2800)
rs2495516	55449618	BB (2385)	BB (3236)	BB (3236)	BB (3236)	BB (3290)	BB (2800)
rs2495513	55457815	AA (2385)	AA (3236)	AA (3236)	AA (3236)	AA (3290)	AA (2800)
rs9887913	55462380	BB (2385)	BB (3236)	BB (3236)	BB (3236)	BB (3290)	BB (2800)
rs2500340	55464743	AA (2385)	AA (3236)	AA (3236)	AA (3236)	AA (3290)	AA (2800)
rs9661809	55471299	AA (2385)	AA (3236)	AA (3236)	AA (3236)	AA (3290)	AA (2800)
rs6682884	55474325	BB (2385)	BB (3236)	BB (3236)	BB (3236)	BB (3290)	BB (2800)
rs2495505	55484810	AA (2385)	AA (3236)	AA (3236)	AA (3236)	AA (3290)	AA (2800)
rs2479394	55486064	AA (2385)	AA (3236)	AA (3236)	AA (3236)	AA (3290)	AA (2800)
rs2479393	55488369	AA (2385)	AA (3236)	AA (3236)	AA (3236)	AA (3290)	AA (2800)
rs2479417	55495744	BB (2385)	BB (3236)	BB (3236)	BB (3236)	BB (3290)	BB (2800)
rs11206510	55496039	AA (2385)	AA (3236)	AA (3236)	AA (3236)	AA (3290)	AA (2800)
rs17111490	55496176	BB (2385)	BB (3236)	BB (3236)	BB (3236)	-3290	BB (2800)
rs2479409	55504650	BB (2385)	BB (3236)	BB (3236)	BB (3236)	BB (3290)	BB (2800)
rs2495480	55509355	AA (2385)	AA (3236)	AA (3236)	AA (3236)	AA (3290)	AA (2800)
rs12066265	55513169	AA (2385)	AA (3236)	AA (3236)	AA (3236)	AA (3290)	AA (2800)
rs7552841	55518752	BB (2385)	BB (3236)	BB (3236)	BB (3236)	BB (3290)	BB (2800)
rs17111557	55529871	BB (2385)	BB (3236)	BB (3236)	BB (3236)	BB (3290)	BB (2800)
rs13312	55532742	AA (2385)	AA (3236)	AA (3236)	AA (3236)	AA (3290)	AA (2800)
rs17111568	55537198	BB (2385)	BB (3236)	BB (3236)	BB (3236)	BB (3290)	BB (2800)
rs17111573	55541681	BB (2385)	BB (3236)	BB (3236)	BB (3236)	BB (3290)	BB (2800)
rs6694813	55542603	AA (2385)	AA (3236)	AA (3236)	AA (3236)	AA (3290)	AA (2800)
rs683880	55546895	AA (2385)	AA (3236)	AA (3236)	AA (3236)	AA (3290)	AA (2800)
rs679804	55552569	AA (2385)	AA (3236)	AA (3236)	AA (3236)	AA (3290)	AA (2800)
rs17111592	55552636	BB (2385)	BB (3236)	BB (3236)	BB (3236)	-3290	BB (2800)
rs6588546	55553226	BB (2385)	BB (3236)	BB (3236)	BB (3236)	BB (3290)	BB (2800)
rs1165282	55567143	BB (2385)	BB (3236)	BB (3236)	BB (3236)	BB (3290)	BB (2800)
rs615652	55567410	BB (2385)	BB (3236)	BB (3236)	BB (3236)	BB (3290)	BB (2800)
rs594226	55572867	AA (2385)	AA (3236)	AA (3236)	AA (3236)	AA (3290)	AA (2800)
rs730717	55574137	AA (2385)	AA (3236)	AA (3236)	AA (3236)	AA (3290)	AA (2800)
rs640238	55593546	BB (2385)	BB (3236)	BB (3236)	BB (3236)	BB (3290)	BB (2800)
rs1165219	55594836	BB (2385)	BB (3236)	BB (3236)	BB (3236)	BB (3290)	BB (2800)
rs1475702	55596587	BB (2385)	BB (3236)	BB (3236)	BB (3236)	BB (3290)	BB (2800)
rs1475703	55596716	BB (2385)	BB (3236)	BB (3236)	BB (3236)	BB (3290)	BB (2800)

rs1165237	55599219	AA (2385)	AA (3236)	AA (3236)	AA (3236)	AA (3290)	AA (2800)
rs17403634	55627360	BB (2385)	BB (3236)	BB (3236)	BB (3236)	BB (3290)	BB (2800)
rs1759497	55629967	AA (2385)	AA (3236)	AA (3236)	AA (3236)	AA (3290)	AA (2800)
rs287231	55664060	AA (2385)	AA (3236)	AA (3236)	AA (3236)	AA (3290)	AA (2800)
rs287235	55678499	AA (2385)	AA (3236)	AA (3236)	AA (3236)	AA (3290)	AA (2800)
rs17111725	55686616	BB (2385)	BB (3236)	BB (3236)	BB (3236)	BB (3290)	BB (2800)
rs10788998	55699023	BB (2385)	BB (3236)	BB (3236)	BB (3236)	BB (3290)	BB (2800)
rs11206534	55712080	BB (2385)	BB (3236)	BB (3236)	BB (3236)	BB (3290)	BB (2800)
rs11206536	55712335	BB (2385)	BB (3236)	BB (3236)	BB (3236)	BB (3290)	BB (2800)
rs11206537	55712358	BB (2385)	BB (3236)	BB (3236)	BB (3236)	BB (3290)	BB (2800)
rs4927208	55713947	BB (2385)	BB (3236)	BB (3236)	BB (3236)	BB (3290)	BB (2800)
rs11206538	55715412	AA (2385)	AA (3236)	AA (3236)	AA (3236)	AA (3290)	AA (2800)
rs2647287	55715608	BB (2385)	BB (3236)	BB (3236)	BB (3236)	BB (3290)	BB (2800)
rs12239399	55719042	AA (2385)	AA (3236)	AA (3236)	AA (3236)	AA (3290)	AA (2800)
rs4927214	55742322	AA (2385)	AA (3236)	AA (3236)	AA (3236)	AA (3290)	AA (2800)
rs1874774	55743297	BB (2385)	BB (3236)	BB (3236)	BB (3236)	BB (3290)	BB (2800)
rs1874775	55743330	BB (2385)	BB (3236)	BB (3236)	BB (3236)	BB (3290)	BB (2800)
rs17111838	55748993	AA (2385)	AA (3236)	AA (3236)	AA (3236)	AA (3290)	AA (2800)
rs1566209	55749183	AA (2385)	AA (3236)	AA (3236)	AA (3236)	AA (3290)	AA (2800)
rs4927218	55749649	AA (2385)	AA (3236)	AA (3236)	AA (3236)	AA (3290)	AA (2800)
rs17111863	55752896	AA (2385)	AA (3236)	AA (3236)	AA (3236)	AA (3290)	AA (2800)
rs17111875	55757023	BB (2385)	BB (3236)	BB (3236)	BB (3236)	BB (3290)	BB (2800)
rs2864899	55759197	AA (2385)	AA (3236)	AA (3236)	AA (3236)	AA (3290)	AA (2800)
rs1498225	55775050	BB (2385)	BB (3236)	BB (3236)	BB (3236)	BB (3290)	BB (2800)
rs6682708	55779387	AA (2385)	AA (3236)	AA (3236)	AA (3236)	AA (3290)	AA (2800)
rs1807871	55780821	AA (2385)	AA (3236)	AA (3236)	AA (3236)	AA (3290)	AA (2800)
rs952574	55780933	AA (2385)	AA (3236)	AA (3236)	AA (3236)	AA (3290)	AA (2800)
rs17111903	55784526	BB (2385)	BB (3236)	BB (3236)	BB (3236)	BB (3290)	BB (2800)
rs207152	55784929	AA (2385)	AA (3236)	AA (3236)	AA (3236)	AA (3290)	AA (2800)
rs6676932	55785672	BB (2385)	BB (3236)	BB (3236)	BB (3236)	BB (3290)	BB (2800)
rs17111909	55785774	BB (2385)	BB (3236)	BB (3236)	BB (3236)	AB (3290)	BB (2800)
rs726815	55790861	BB (2385)	BB (3236)	BB (3236)	BB (3236)	BB (3290)	BB (2800)
rs7534376	55792816	BB (2385)	BB (3236)	BB (3236)	BB (3236)	BB (3290)	BB (2800)
rs207164	55799770	AA (2385)	AA (3236)	AA (3236)	AA (3236)	AA (3290)	AA (2800)
rs207149	55807259	AA (2385)	AA (3236)	AA (3236)	AA (3236)	AA (3290)	AA (2800)
rs207148	55807501	AA (2385)	AA (3236)	AA (3236)	AA (3236)	AA (3290)	AA (2800)
rs207136	55814164	BB (2385)	BB (3236)	BB (3236)	BB (3236)	BB (3290)	BB (2800)
rs207135	55814244	AA (2385)	AA (3236)	AA (3236)	AA (3236)	AA (3290)	AA (2800)
rs17111989	55818887	AA (2385)	AA (3236)	AA (3236)	AA (3236)	AA (3290)	AA (2800)
rs880385	55820099	BB (2385)	BB (3236)	BB (3236)	BB (3236)	BB (3290)	BB (2800)
rs880386	55820264	BB (2385)	BB (3236)	BB (3236)	BB (3236)	BB (3290)	BB (2800)
rs41438246	55820441	BB (2385)	BB (3236)	BB (3236)	BB (3236)	BB (3290)	BB (2800)
rs2179853	55820713	BB (2385)	BB (3236)	BB (3236)	BB (3236)	BB (3290)	BB (2800)
rs207190	55822285	AA (2385)	AA (3236)	AA (3236)	AA (3236)	AA (3290)	AA (2800)
rs2746688	55831678	AA (2385)	AA (3236)	AA (3236)	AA (3236)	AA (3290)	AA (2800)
rs2802881	55831918	BB (2385)	BB (3236)	BB (3236)	BB (3236)	BB (3290)	BB (2800)
rs910270	55833351	BB (2385)	BB (3236)	BB (3236)	BB (3236)	BB (3290)	BB (2800)
rs2746685	55837603	AA (2385)	AA (3236)	AA (3236)	AA (3236)	AA (3290)	AA (2800)
rs12758665	55839117	AA (2385)	AA (3236)	AA (3236)	AA (3236)	AA (3290)	AA (2800)
rs2746683	55841735	AA (2385)	AA (3236)	AA (3236)	AA (3236)	AA (3290)	AA (2800)

rs2802877	55843810		BB (2385)	BB (3236)	BB (3236)	BB (3236)	BB (3290)	BB (2800)
rs11206577	55860561		BB (2385)	BB (3236)	BB (3236)	BB (3236)	BB (3290)	BB (2800)
rs11580406	55865474		AA (2385)	AA (3236)	AA (3236)	AA (3236)	AA (3290)	AA (2800)
rs12089328	55865582		BB (2385)	BB (3236)	BB (3236)	BB (3236)	BB (3290)	BB (2800)
rs11206579	55884333		AA (2385)	AA (3236)	AA (3236)	AA (3236)	AA (3290)	AA (2800)
rs11206580	55891799		AA (2385)	AA (3236)	AA (3236)	AA (3236)	AA (3290)	AA (2800)
rs17112051	55892471		BB (2385)	BB (3236)	BB (3236)	BB (3236)	BB (3290)	BB (2800)
rs4927240	55920290		AA (2385)	AA (3236)	AA (3236)	AA (3236)	AA (3290)	AA (2800)
rs12068336	55929930		BB (2385)	BB (3236)	BB (3236)	BB (3236)	BB (3290)	BB (2800)
rs11206601	55931747		BB (2385)	BB (3236)	BB (3236)	BB (3236)	BB (3290)	BB (2800)
rs874664	55933344		AA (2385)	AA (3236)	AA (3236)	AA (3236)	AA (3290)	AA (2800)
rs904599	55933491		AB (2385)	AA (3236)	AB (3236)	AB (3236)	AB (3290)	-2800
rs904611	55933759		BB (2385)	BB (3236)	BB (3236)	BB (3236)	BB (3290)	BB (2800)
rs12568687	55934389		AA (2385)	AA (3236)	AA (3236)	AA (3236)	AA (3290)	AA (2800)
rs7532502	55938887		AA (2385)	AA (3236)	AA (3236)	AA (3236)	AA (3290)	AA (2800)
rs1499678	55939994		BB (2385)	BB (3236)	BB (3236)	BB (3236)	BB (3290)	BB (2800)
rs10493199	55940340		BB (2385)	BB (3236)	BB (3236)	BB (3236)	BB (3290)	BB (2800)
rs1392141	55940369		BB (2385)	BB (3236)	BB (3236)	BB (3236)	BB (3290)	BB (2800)
rs1980549	55945134		AA (2385)	AA (3236)	AA (3236)	AA (3236)	AA (3290)	AA (2800)
rs380389	55957272		AA (2385)	AA (3236)	AA (3236)	AA (3236)	AA (3290)	AA (2800)
rs17112188	55959240		AA (2385)	AA (3236)	AA (3236)	AA (3236)	AA (3290)	AA (2800)
rs1780542	55963519		AA (2385)	AA (3236)	AA (3236)	AA (3236)	AA (3290)	AA (2800)
rs1114737	55965413		BB (2385)	BB (3236)	BB (3236)	BB (3236)	BB (3290)	BB (2800)
rs17416166	55966832		BB (2385)	BB (3236)	BB (3236)	BB (3236)	BB (3290)	BB (2800)
rs17112228	55971058		BB (2385)	BB (3236)	BB (3236)	BB (3236)	BB (3290)	BB (2800)
rs17112230	55971620		BB (2385)	BB (3236)	BB (3236)	BB (3236)	BB (3290)	BB (2800)
rs356117	55971759		AA (2385)	AA (3236)	AA (3236)	AA (3236)	AA (3290)	AA (2800)
rs17112231	55971798		BB (2385)	BB (3236)	BB (3236)	BB (3236)	BB (3290)	BB (2800)
rs1740122	55976706		AA (2385)	AA (3236)	AA (3236)	AA (3236)	AA (3290)	AA (2800)
rs1740118	55981250		BB (2385)	BB (3236)	BB (3236)	BB (3236)	BB (3290)	BB (2800)
rs1695960	55983525		BB (2385)	BB (3236)	BB (3236)	BB (3236)	BB (3290)	BB (2800)
rs2133555	55984309		BB (2385)	BB (3236)	BB (3236)	BB (3236)	BB (3290)	BB (2800)
rs390415	55984513		AA (2385)	AA (3236)	AA (3236)	AA (3236)	AA (3290)	AA (2800)
rs12757934	55984933		AA (2385)	AA (3236)	AA (3236)	AA (3236)	AA (3290)	AA (2800)
rs4927246	55985066		AA (2385)	AA (3236)	AA (3236)	AA (3236)	AA (3290)	AA (2800)
rs904610	55989414		AA (2385)	AA (3236)	AA (3236)	AA (3236)	AA (3290)	AA (2800)
rs356081	55989646		AA (2385)	AA (3236)	AA (3236)	AA (3236)	AA (3290)	AA (2800)
rs356085	55992755		BB (2385)	BB (3236)	BB (3236)	BB (3236)	BB (3290)	BB (2800)
rs1979827	55993010		AA (2385)	AA (3236)	AA (3236)	AA (3236)	AA (3290)	AA (2800)
rs6588561	55993095		BB (2385)	BB (3236)	BB (3236)	BB (3236)	BB (3290)	BB (2800)
rs1995229	55995298		AA (2385)	AA (3236)	AA (3236)	AA (3236)	AA (3290)	AA (2800)
rs181453	55995343		BB (2385)	BB (3236)	BB (3236)	BB (3236)	BB (3290)	BB (2800)
rs356098	55998339		AA (2385)	AA (3236)	AA (3236)	AA (3236)	AA (3290)	AA (2800)
rs2649627	55998405		BB (2385)	BB (3236)	BB (3236)	BB (3236)	BB (3290)	BB (2800)
rs168549	55998435		BB (2385)	BB (3236)	BB (3236)	BB (3236)	BB (3290)	BB (2800)
rs356096	55999067		BB (2385)	BB (3236)	BB (3236)	BB (3236)	BB (3290)	BB (2800)
rs7542534	56022895		BB (2385)	BB (3236)	BB (3236)	BB (3236)	BB (3290)	BB (2800)
rs10888934	56033892		AA (2385)	AA (3236)	AA (3236)	AA (3236)	AA (3290)	AA (2800)
rs7538836	56033932		BB (2385)	BB (3236)	BB (3236)	BB (3236)	BB (3290)	BB (2800)
rs10493196	56046405		AA (2385)	AA (3236)	AA (3236)	AA (3236)	AA (3290)	AA (2800)

rs1695945	56050016		AA (2385)	AA (3236)	AA (3236)	AA (3236)	AA (3290)	AA (2800)
rs1695946	56052308		BB (2385)	BB (3236)	BB (3236)	BB (3236)	BB (3290)	BB (2800)
rs1499663	56057990		AA (2385)	AA (3236)	AA (3236)	AA (3236)	AA (3290)	AA (2800)
rs7548272	56059283		BB (2385)	BB (3236)	BB (3236)	BB (3236)	BB (3290)	BB (2800)
rs6588567	56059425		AA (2385)	AA (3236)	AA (3236)	AA (3236)	AA (3290)	AA (2800)
rs2047543	56059913		AA (2385)	AA (3236)	AA (3236)	AA (3236)	AA (3290)	AA (2800)
rs10888935	56060951		AA (2385)	AA (3236)	AA (3236)	AA (3236)	AA (3290)	AA (2800)
rs11206628	56060981		BB (2385)	BB (3236)	BB (3236)	BB (3236)	BB (3290)	BB (2800)
rs1392129	56066714		AA (2385)	AA (3236)	AA (3236)	AA (3236)	AA (3290)	AA (2800)
rs11206633	56069367		BB (2385)	BB (3236)	BB (3236)	BB (3236)	BB (3290)	BB (2800)
rs1499667	56077813		AA (2385)	AA (3236)	AA (3236)	AA (3236)	AA (3290)	AA (2800)
rs1392132	56079739		AA (2385)	AA (3236)	AA (3236)	AA (3236)	AA (3290)	AA (2800)
rs769968	56092175		AA (2385)	AA (3236)	AA (3236)	AA (3236)	AA (3290)	AA (2800)
rs17418151	56102010		BB (2385)	BB (3236)	BB (3236)	BB (3236)	BB (3290)	BB (2800)
rs6693323	56102892		AA (2385)	AA (3236)	AA (3236)	AA (3236)	AA (3290)	AA (2800)
rs1039296	56103659		BB (2385)	BB (3236)	BB (3236)	BB (3236)	BB (3290)	BB (2800)
rs7416182	56104116		AA (2385)	AA (3236)	AA (3236)	AA (3236)	AA (3290)	AA (2800)
rs6669312	56106677		BB (2385)	BB (3236)	BB (3236)	BB (3236)	BB (3290)	BB (2800)
rs12124393	56113365		AA (2385)	AA (3236)	AA (3236)	AA (3236)	AA (3290)	AA (2800)
rs1828802	56115427		BB (2385)	BB (3236)	BB (3236)	BB (3236)	BB (3290)	BB (2800)
rs769977	56115798		BB (2385)	BB (3236)	BB (3236)	BB (3236)	BB (3290)	BB (2800)
rs17112591	56118105		BB (2385)	BB (3236)	BB (3236)	BB (3236)	BB (3290)	BB (2800)
rs6660332	56121180		BB (2385)	BB (3236)	BB (3236)	BB (3236)	BB (3290)	BB (2800)
rs17452876	56122328		AA (2385)	AA (3236)	AA (3236)	AA (3236)	AA (3290)	AA (2800)
rs1514140	56136068		BB (2385)	BB (3236)	BB (3236)	BB (3236)	BB (3290)	BB (2800)
rs6701011	56138676		BB (2385)	BB (3236)	BB (3236)	BB (3236)	BB (3290)	BB (2800)
rs4367813	56138735		AA (2385)	AA (3236)	AA (3236)	AA (3236)	AA (3290)	AA (2800)
rs2767501	56151009		BB (2385)	BB (3236)	BB (3236)	BB (3236)	BB (3290)	BB (2800)
rs2671239	56153246		BB (2385)	BB (3236)	BB (3236)	BB (3236)	BB (3290)	BB (2800)
rs2767515	56165742		AA (2385)	AA (3236)	AA (3236)	AA (3236)	AA (3290)	AA (2800)
rs12239009	56166508		BB (2385)	BB (3236)	BB (3236)	BB (3236)	BB (3290)	BB (2800)
rs17112673	56172574		AA (2385)	AA (3236)	AA (3236)	AA (3236)	AA (3290)	AA (2800)
rs3936673	56183018		BB (2385)	BB (3236)	BB (3236)	BB (3236)	BB (3290)	BB (2800)
rs3862236	56183394		AA (2385)	AA (3236)	AA (3236)	AA (3236)	AA (3290)	AA (2800)
rs4111070	56195659		BB (2385)	BB (3236)	BB (3236)	BB (3236)	BB (3290)	BB (2800)
rs2767499	56201024		AA (2385)	AA (3236)	AA (3236)	AA (3236)	AA (3290)	AA (2800)
rs17112732	56204918		BB (2385)	BB (3236)	BB (3236)	BB (3236)	BB (3290)	BB (2800)
rs9436990	56231008		AA (2385)	AA (3236)	AA (3236)	AA (3236)	AA (3290)	AA (2800)
rs1165515	56231812		BB (2385)	BB (3236)	BB (3236)	BB (3236)	BB (3290)	BB (2800)
rs12061007	56233601		AA (2385)	AA (3236)	AA (3236)	AA (3236)	AA (3290)	AA (2800)
rs41331246	56261172		BB (2385)	BB (3236)	BB (3236)	AB (3236)	-3290	AB (2800)
rs41365145	56266970		BB (2385)	BB (3236)	BB (3236)	BB (3236)	BB (3290)	BB (2800)
rs4412634	56284965		AA (2385)	AA (3236)	AA (3236)	AA (3236)	AA (3290)	AA (2800)
rs4244649	56284988		BB (2385)	BB (3236)	BB (3236)	BB (3236)	BB (3290)	BB (2800)
rs4244650	56290747		AA (2385)	AA (3236)	AA (3236)	AA (3236)	AA (3290)	AA (2800)
rs6659096	56292073		BB (2385)	BB (3236)	BB (3236)	BB (3236)	BB (3290)	BB (2800)
rs11587235	56300900		BB (2385)	BB (3236)	BB (3236)	BB (3236)	BB (3290)	BB (2800)
rs17112932	56320291		AA (2385)	AA (3236)	AA (3236)	AA (3236)	AA (3290)	AA (2800)
rs10493188	56320679		BB (2385)	BB (3236)	BB (3236)	BB (3236)	BB (3290)	BB (2800)
rs7349129	56320873		AA (2385)	AA (3236)	AA (3236)	AA (3236)	AA (3290)	AA (2800)

rs10493187	56320969		AA (2385)	AA (3236)	AA (3236)	AA (3236)	AA (3290)	AA (2800)
rs10493186	56321074		AA (2385)	AA (3236)	AA (3236)	AA (3236)	AA (3290)	AA (2800)
rs6662150	56322699		AA (2385)	AA (3236)	AA (3236)	AA (3236)	AA (3290)	AA (2800)
rs9326038	56326293		BB (2385)	BB (3236)	BB (3236)	BB (3236)	BB (3290)	BB (2800)
rs11206673	56326340		BB (2385)	BB (3236)	BB (3236)	BB (3236)	BB (3290)	BB (2800)
rs4926698	56328596		BB (2385)	BB (3236)	BB (3236)	BB (3236)	BB (3290)	BB (2800)
rs10493182	56335942		AA (2385)	AA (3236)	AA (3236)	AA (3236)	AA (3290)	AA (2800)
rs6690389	56351863		BB (2385)	BB (3236)	BB (3236)	BB (3236)	BB (3290)	BB (2800)
rs17113071	56353786		AA (2385)	AA (3236)	AA (3236)	AA (3236)	AA (3290)	AA (2800)
rs6681658	56359597		AA (2385)	AA (3236)	AA (3236)	AA (3236)	AA (3290)	AA (2800)
rs10888947	56375280		AA (2385)	AA (3236)	AA (3236)	AA (3236)	AA (3290)	AA (2800)
rs11800773	56382975		AA (2385)	AA (3236)	AA (3236)	AA (3236)	AA (3290)	AA (2800)
rs4468206	56397362		AA (2385)	AA (3236)	AA (3236)	AA (3236)	AA (3290)	AA (2800)
rs4526652	56405703		BB (2385)	BB (3236)	BB (3236)	BB (3236)	BB (3290)	BB (2800)
rs4345838	56408458		AA (2385)	AA (3236)	AA (3236)	AA (3236)	AA (3290)	AA (2800)
rs12565147	56408597		AA (2385)	AA (3236)	AA (3236)	AA (3236)	AA (3290)	AA (2800)
rs7526613	56421137		BB (2385)	BB (3236)	BB (3236)	BB (3236)	BB (3290)	BB (2800)
rs7534660	56421196		AA (2385)	AA (3236)	AA (3236)	AA (3236)	AB (3290)	AA (2800)
rs7512875	56421399		AA (2385)	AA (3236)	AA (3236)	AA (3236)	AA (3290)	AA (2800)
rs6694316	56425121		AA (2385)	AA (3236)	AA (3236)	AA (3236)	AA (3290)	AA (2800)
rs10789012	56434711		AA (2385)	AA (3236)	AA (3236)	AA (3236)	AA (3290)	AA (2800)
rs11206697	56434812		BB (2385)	BB (3236)	BB (3236)	BB (3236)	BB (3290)	BB (2800)
rs12032226	56438651		BB (2385)	BB (3236)	BB (3236)	BB (3236)	BB (3290)	BB (2800)
rs6664415	56438761		BB (2385)	BB (3236)	BB (3236)	BB (3236)	BB (3290)	BB (2800)
rs6664825	56439329		BB (2385)	BB (3236)	BB (3236)	BB (3236)	AB (3290)	BB (2800)
rs6664937	56439426		BB (2385)	BB (3236)	BB (3236)	BB (3236)	BB (3290)	BB (2800)
rs10489777	56439909		BB (2385)	BB (3236)	BB (3236)	BB (3236)	BB (3290)	BB (2800)
rs3992634	56446204		BB (2385)	BB (3236)	BB (3236)	BB (3236)	BB (3290)	BB (2800)
rs10749703	56446296		AA (2385)	AA (3236)	AA (3236)	AA (3236)	AA (3290)	AA (2800)
rs10489776	56490778		BB (2385)	BB (3236)	BB (3236)	BB (3236)	BB (3290)	BB (2800)
rs17113283	56494770		AA (2385)	AA (3236)	AA (3236)	AA (3236)	AA (3290)	AA (2800)
rs17113287	56495580		BB (2385)	BB (3236)	BB (3236)	BB (3236)	BB (3290)	BB (2800)
rs11206719	56502240		AA (2385)	AA (3236)	AA (3236)	AA (3236)	AA (3290)	AA (2800)
rs6688604	56502528		AA (2385)	AA (3236)	AA (3236)	AA (3236)	AA (3290)	AA (2800)
rs2110032	56506126		BB (2385)	BB (3236)	BB (3236)	BB (3236)	BB (3290)	BB (2800)
rs2159870	56506262		AA (2385)	AA (3236)	AA (3236)	AA (3236)	AA (3290)	AA (2800)
rs1331853	56518934		AA (2385)	AA (3236)	AA (3236)	AA (3236)	AA (3290)	AA (2800)
rs4278395	56519023		AA (2385)	AA (3236)	AA (3236)	AA (3236)	AA (3290)	AA (2800)
rs41533449	56531423		AA (2385)	AA (3236)	AA (3236)	AA (3236)	AA (3290)	AA (2800)
rs1331855	56531889		BB (2385)	BB (3236)	BB (3236)	BB (3236)	BB (3290)	BB (2800)
rs17113308	56535752		AA (2385)	AA (3236)	AA (3236)	AA (3236)	AA (3290)	AA (2800)
rs1412212	56560116		AA (2385)	AA (3236)	AA (3236)	AA (3236)	AA (3290)	AA (2800)
rs1412216	56565917		AA (2385)	AA (3236)	AA (3236)	AA (3236)	AA (3290)	AA (2800)
rs1159984	56566090		AA (2385)	AA (3236)	AA (3236)	AA (3236)	AA (3290)	AA (2800)
rs4927303	56581972		AA (2385)	AA (3236)	AA (3236)	AA (3236)	AA (3290)	AA (2800)
rs6682819	56593048		AA (2385)	AA (3236)	AA (3236)	AA (3236)	AA (3290)	AA (2800)
rs946197	56602308		AA (2385)	AA (3236)	AA (3236)	AA (3236)	AB (3290)	AA (2800)
rs1890347	56604150		BB (2385)	BB (3236)	BB (3236)	BB (3236)	BB (3290)	BB (2800)
rs1537226	56615004		BB (2385)	BB (3236)	BB (3236)	BB (3236)	BB (3290)	BB (2800)
rs6703713	56616961		AA (2385)	AA (3236)	AA (3236)	AA (3236)	AA (3290)	AA (2800)

rs17113399	56619216		BB (2385)	BB (3236)	BB (3236)	BB (3236)	BB (3290)	BB (2800)
rs778119	56621178		AA (2385)	AA (3236)	AA (3236)	AA (3236)	AA (3290)	AA (2800)
rs778382	56635806		BB (2385)	BB (3236)	BB (3236)	BB (3236)	BB (3290)	BB (2800)
rs6588610	56636829		BB (2385)	BB (3236)	BB (3236)	BB (3236)	BB (3290)	BB (2800)
rs778417	56644567		BB (2385)	BB (3236)	BB (3236)	BB (3236)	BB (3290)	BB (2800)
rs778420	56647914		AA (2385)	AA (3236)	AA (3236)	AA (3236)	AA (3290)	AA (2800)
rs17362438	56654191		AA (2385)	AA (3236)	AA (3236)	AA (3236)	AA (3290)	AA (2800)
rs17362459	56654259		BB (2385)	BB (3236)	BB (3236)	BB (3236)	BB (3290)	BB (2800)
rs778423	56656812		BB (2385)	BB (3236)	BB (3236)	BB (3236)	BB (3290)	BB (2800)
rs1149648	56657399		AA (2385)	AA (3236)	AA (3236)	AA (3236)	AA (3290)	AA (2800)
rs17113474	56660489		AA (2385)	AA (3236)	AA (3236)	AA (3236)	AA (3290)	AA (2800)
rs17362494	56660640		AA (2385)	AA (3236)	AA (3236)	AA (3236)	AA (3290)	AA (2800)
rs778415	56673450		AA (2385)	AA (3236)	AA (3236)	AA (3236)	AA (3290)	AA (2800)
rs706370	56689205		BB (2385)	BB (3236)	BB (3236)	BB (3236)	BB (3290)	BB (2800)
rs947255	56689336		BB (2385)	BB (3236)	BB (3236)	BB (3236)	BB (3290)	BB (2800)
rs697581	56689365		AA (2385)	AA (3236)	AA (3236)	AA (3236)	AA (3290)	AA (2800)
rs706369	56690319		BB (2385)	BB (3236)	BB (3236)	BB (3236)	BB (3290)	BB (2800)
rs1230034	56690722		AA (2385)	AA (3236)	AA (3236)	AA (3236)	AA (3290)	AA (2800)
rs7553732	56698256		BB (2385)	BB (3236)	BB (3236)	BB (3236)	BB (3290)	BB (2800)
rs4927308	56700374		BB (2385)	BB (3236)	BB (3236)	BB (3236)	BB (3290)	BB (2800)
rs778403	56702613		BB (2385)	BB (3236)	BB (3236)	BB (3236)	BB (3290)	BB (2800)
rs17113538	56702767		BB (2385)	BB (3236)	BB (3236)	BB (3236)	BB (3290)	BB (2800)
rs778404	56702785		BB (2385)	BB (3236)	BB (3236)	BB (3236)	BB (3290)	BB (2800)
rs2792773	56703396		BB (2385)	BB (3236)	BB (3236)	BB (3236)	BB (3290)	BB (2800)
rs778405	56703413		BB (2385)	BB (3236)	BB (3236)	BB (3236)	BB (3290)	BB (2800)
rs1417768	56716407		AA (2385)	AA (3236)	AA (3236)	AA (3236)	AA (3290)	AA (2800)
rs1341337	56716426		AA (2385)	AA (3236)	AA (3236)	AA (3236)	AA (3290)	AA (2800)
rs1417769	56716571		BB (2385)	BB (3236)	BB (3236)	BB (3236)	BB (3290)	BB (2800)
rs17113557	56719210		AA (2385)	AA (3236)	AA (3236)	AA (3236)	AA (3290)	AA (2800)
rs2792795	56723851		BB (2385)	BB (3236)	BB (3236)	BB (3236)	BB (3290)	BB (2800)
rs2793661	56724405		BB (2385)	BB (3236)	BB (3236)	BB (3236)	BB (3290)	BB (2800)
rs1341340	56728650		BB (2385)	BB (3236)	BB (3236)	BB (3236)	BB (3290)	BB (2800)
rs1331863	56740692		AA (2385)	AA (3236)	AA (3236)	AA (3236)	AA (3290)	AA (2800)
rs2792788	56740723		AA (2385)	AA (3236)	AA (3236)	AA (3236)	AA (3290)	AA (2800)
rs2793669	56741692		BB (2385)	BB (3236)	BB (3236)	BB (3236)	BB (3290)	BB (2800)
rs1557061	56748182		AA (2385)	AA (3236)	AA (3236)	AA (3236)	AA (3290)	AA (2800)
rs11206774	56748275		BB (2385)	BB (3236)	BB (3236)	BB (3236)	BB (3290)	BB (2800)
rs1539058	56753249		BB (2385)	BB (3236)	BB (3236)	BB (3236)	BB (3290)	BB (2800)
rs2000141	56756491		AA (2385)	AA (3236)	AA (3236)	AA (3236)	AA (3290)	AA (2800)
rs2793675	56757968		AA (2385)	AA (3236)	AA (3236)	AA (3236)	AA (3290)	AA (2800)
rs17424899	56758246		BB (2385)	BB (3236)	BB (3236)	BB (3236)	BB (3290)	BB (2800)
rs1539059	56759268		AA (2385)	AA (3236)	AA (3236)	AA (3236)	AA (3290)	AA (2800)
rs2793684	56761923		BB (2385)	BB (3236)	BB (3236)	BB (3236)	BB (3290)	BB (2800)
rs17459027	56762342		BB (2385)	BB (3236)	BB (3236)	BB (3236)	BB (3290)	BB (2800)
rs2793685	56762443		AA (2385)	AA (3236)	AA (3236)	AA (3236)	AA (3290)	AA (2800)
rs17096767	56766727		BB (2385)	BB (3236)	BB (3236)	BB (3236)	BB (3290)	BB (2800)
rs17425772	56769856		AA (2385)	AA (3236)	AA (3236)	AA (3236)	AA (3290)	AA (2800)
rs10789018	56770155		BB (2385)	BB (3236)	BB (3236)	BB (3236)	BB (3290)	BB (2800)
rs11206781	56770373		BB (2385)	BB (3236)	BB (3236)	BB (3236)	BB (3290)	BB (2800)
rs4291536	56774902		BB (2385)	BB (3236)	BB (3236)	BB (3236)	BB (3290)	BB (2800)

rs12739422	56781163		BB (2385)	BB (3236)	BB (3236)	BB (3236)	BB (3290)	BB (2800)
rs10493203	56782579		AA (2385)	AA (3236)	AA (3236)	AA (3236)	AA (3290)	AA (2800)
rs2994554	56785356		AA (2385)	AA (3236)	AA (3236)	AA (3236)	AA (3290)	AA (2800)
rs3005896	56785831		AA (2385)	AA (3236)	AA (3236)	AA (3236)	AA (3290)	AA (2800)
rs12138574	56790773		AA (2385)	AA (3236)	AA (3236)	AA (3236)	AA (3290)	AA (2800)
rs3005907	56790785		AA (2385)	AA (3236)	AA (3236)	AA (3236)	AA (3290)	AA (2800)
rs6670302	56799260		AA (2385)	AA (3236)	AA (3236)	AA (3236)	AA (3290)	AA (2800)
rs7525535	56799799		AA (2385)	AA (3236)	AA (3236)	AA (3236)	AA (3290)	AA (2800)
rs10493206	56819151		BB (2385)	BB (3236)	BB (3236)	BB (3236)	BB (3290)	BB (2800)
rs10493208	56820651		AA (2385)	AA (3236)	AA (3236)	AA (3236)	AA (3290)	AA (2800)
rs4477326	56821054		AA (2385)	AA (3236)	AA (3236)	AA (3236)	AA (3290)	AA (2800)
rs17113783	56823378		BB (2385)	BB (3236)	BB (3236)	BB (3236)	BB (3290)	BB (2800)
rs4912230	56826300		AA (2385)	AA (3236)	AA (3236)	AA (3236)	AA (3290)	AA (2800)
rs17113792	56829396		AA (2385)	AA (3236)	AA (3236)	AA (3236)	AA (3290)	AA (2800)
rs6684867	56836577		BB (2385)	BB (3236)	BB (3236)	BB (3236)	BB (3290)	BB (2800)
rs4600084	56844621		BB (2385)	BB (3236)	BB (3236)	BB (3236)	BB (3290)	BB (2800)
rs17113799	56844652		BB (2385)	BB (3236)	BB (3236)	BB (3236)	BB (3290)	BB (2800)
rs4353140	56852126		BB (2385)	BB (3236)	BB (3236)	BB (3236)	BB (3290)	BB (2800)
rs10218770	56858351		AA (2385)	AA (3236)	AA (3236)	AA (3236)	AA (3290)	AA (2800)
rs17113828	56858960		BB (2385)	BB (3236)	BB (3236)	BB (3236)	BB (3290)	BB (2800)
rs41335147	56859227		AA (2385)	AA (3236)	AA (3236)	AA (3236)	AA (3290)	AA (2800)
rs10493210	56861009		AA (2385)	AA (3236)	AA (3236)	AA (3236)	AA (3290)	AA (2800)
rs17113842	56862154		BB (2385)	BB (3236)	BB (3236)	BB (3236)	BB (3290)	BB (2800)
rs17428956	56875043		AA (2385)	AA (3236)	AA (3236)	AA (3236)	AA (3290)	AA (2800)
rs4630156	56876498		AA (2385)	AA (3236)	AA (3236)	AA (3236)	AA (3290)	AA (2800)
rs11206803	56877509		AA (2385)	AA (3236)	AA (3236)	AA (3236)	AA (3290)	AA (2800)
rs884277	56881763		BB (2385)	BB (3236)	BB (3236)	BB (3236)	BB (3290)	BB (2800)
rs10789020	56883524		BB (2385)	BB (3236)	BB (3236)	BB (3236)	BB (3290)	BB (2800)
rs11206808	56904902		AA (2385)	AA (3236)	AA (3236)	AA (3236)	AA (3290)	AA (2800)
rs2780293	56905072		BB (2385)	BB (3236)	BB (3236)	BB (3236)	BB (3290)	BB (2800)
rs6421495	56905445		AA (2385)	AA (3236)	AA (3236)	AA (3236)	AA (3290)	AA (2800)
rs7527084	56930365		BB (2385)	BB (3236)	BB (3236)	BB (3236)	BB (3290)	BB (2800)
rs7513178	56930449		BB (2385)	BB (3236)	BB (3236)	BB (3236)	BB (3290)	BB (2800)
rs7535319	56930618		AA (2385)	AA (3236)	AA (3236)	AA (3236)	AA (3290)	AA (2800)
rs12739002	56936282		AA (2385)	AA (3236)	AA (3236)	AA (3236)	AA (3290)	AA (2800)
rs6588635	56940914		BB (2385)	BB (3236)	BB (3236)	BB (3236)	BB (3290)	BB (2800)
rs7532239	56953939		AA (2385)	AA (3236)	AA (3236)	AA (3236)	AA (3290)	AA (2800)
rs17114014	56958208		AA (2385)	AA (3236)	AA (3236)	AA (3236)	AA (3290)	AA (2800)
rs4912314	56968477		BB (2385)	BB (3236)	BB (3236)	BB (3236)	BB (3290)	BB (2800)
rs4912317	56970278		BB (2385)	BB (3236)	BB (3236)	BB (3236)	BB (3290)	BB (2800)
rs7515808	56972400		BB (2385)	BB (3236)	BB (3236)	BB (3236)	BB (3290)	BB (2800)
rs1319274	56974091		AA (2385)	AA (3236)	AA (3236)	AA (3236)	AA (3290)	AA (2800)
rs12406517	56974278		BB (2385)	BB (3236)	BB (3236)	BB (3236)	BB (3290)	BB (2800)
rs11576602	56974816		BB (2385)	BB (3236)	BB (3236)	BB (3236)	BB (3290)	BB (2800)
rs3738570	56977819		AA (2385)	AA (3236)	AA (3236)	AA (3236)	AA (3290)	AA (2800)
rs4532850	56996104		AA (2385)	AA (3236)	AA (3236)	AA (3236)	AA (3290)	AA (2800)
rs11206836	56996741		AA (2385)	AA (3236)	AA (3236)	AA (3236)	AA (3290)	AA (2800)
rs10888979	57010711		AA (2385)	AA (3236)	AA (3236)	AA (3236)	AA (3290)	AA (2800)
rs1930759	57013616		BB (2385)	BB (3236)	BB (3236)	BB (3236)	BB (3290)	BB (2800)
rs11206839	57015508		BB (2385)	BB (3236)	BB (3236)	BB (3236)	BB (3290)	BB (2800)

rs10493211	57016912	AA (2385)	AA (3236)	AA (3236)	AA (3236)	AA (3290)	AA (2800)
rs11810204	57020516	AA (2385)	AA (3236)	AA (3236)	AA (3236)	AA (3290)	AA (2800)
rs10736393	57023932	BB (2385)	BB (3236)	BB (3236)	BB (3236)	BB (3290)	BB (2800)
rs41446844	57031938	BB (2385)	BB (3236)	BB (3236)	BB (3236)	BB (3290)	BB (2800)
rs1777275	57032468	AA (2385)	AA (3236)	AA (3236)	AA (3236)	AA (3290)	AA (2800)
rs1418521	57040945	BB (2385)	BB (3236)	BB (3236)	BB (3236)	BB (3290)	BB (2800)
rs933001	57042335	BB (2385)	BB (3236)	BB (3236)	BB (3236)	BB (3290)	BB (2800)
rs7548361	57063719	AA (2385)	AA (3236)	AA (3236)	AA (3236)	AA (3290)	AA (2800)
rs6686996	57065295	BB (2385)	BB (3236)	BB (3236)	-3236	BB (3290)	BB (2800)
rs2051038	57067422	BB (2385)	BB (3236)	BB (3236)	BB (3236)	BB (3290)	BB (2800)
rs12038246	57069614	BB (2385)	BB (3236)	BB (3236)	BB (3236)	BB (3290)	BB (2800)
rs2404986	57084155	AA (2385)	AA (3236)	AA (3236)	AA (3236)	AA (3290)	AA (2800)
rs11206874	57087808	BB (2385)	BB (3236)	BB (3236)	BB (3236)	BB (3290)	BB (2800)
rs10888995	57094025	BB (2385)	BB (3236)	BB (3236)	BB (3236)	BB (3290)	BB (2800)
rs2404993	57103965	AA (2385)	AA (3236)	AA (3236)	AA (3236)	AA (3290)	AA (2800)
rs2796529	57126875	BB (2385)	BB (3236)	BB (3236)	BB (3236)	BB (3290)	BB (2800)
rs2746347	57126919	AA (2385)	AA (3236)	AA (3236)	AA (3236)	AA (3290)	AA (2800)
rs2796519	57130588	AA (2385)	AA (3236)	AA (3236)	AA (3236)	AA (3290)	AA (2800)
rs10889008	57132994	BB (2385)	BB (3236)	BB (3236)	BB (3236)	BB (3290)	BB (2800)
rs2179761	57157544	BB (2385)	BB (3236)	BB (3236)	BB (3236)	BB (3290)	BB (2800)
rs1342382	57177388	BB (2385)	BB (3236)	BB (3236)	BB (3236)	BB (3290)	BB (2800)
rs1342514	57187360	BB (2385)	BB (3236)	BB (3236)	BB (3236)	BB (3290)	BB (2800)
rs857126	57202008	BB (2385)	BB (3236)	BB (3236)	BB (3236)	BB (3290)	BB (2800)
rs6658790	57203011	AA (2385)	AA (3236)	AA (3236)	AA (3236)	AA (3290)	AA (2800)
rs857123	57205666	AA (2385)	AA (3236)	AA (3236)	AA (3236)	AA (3290)	AA (2800)
rs11206912	57211204	BB (2385)	BB (3236)	BB (3236)	BB (3236)	BB (3290)	BB (2800)
rs857120	57211399	AA (2385)	AA (3236)	AA (3236)	AA (3236)	AA (3290)	AA (2800)
rs6666850	57215026	AA (2385)	AA (3236)	AA (3236)	AA (3236)	AA (3290)	AA (2800)
rs17114246	57215972	AA (2385)	AA (3236)	AA (3236)	AA (3236)	AA (3290)	AA (2800)
rs17114252	57216224	AA (2385)	AA (3236)	AA (3236)	AA (3236)	AA (3290)	AA (2800)
rs2040007	57216327	BB (2385)	BB (3236)	BB (3236)	BB (3236)	BB (3290)	BB (2800)
rs6696458	57216376	BB (2385)	BB (3236)	BB (3236)	BB (3236)	BB (3290)	BB (2800)
rs7555378	57218159	AA (2385)	AA (3236)	AA (3236)	AA (3236)	AA (3290)	AA (2800)
rs857109	57218497	BB (2385)	BB (3236)	BB (3236)	BB (3236)	BB (3290)	BB (2800)
rs857107	57222599	AA (2385)	AA (3236)	AA (3236)	AA (3236)	AA (3290)	AA (2800)
rs10489619	57222633	AA (2385)	AA (3236)	AA (3236)	AA (3236)	AA (3290)	AA (2800)
rs1557145	57241234	BB (2385)	BB (3236)	BB (3236)	BB (3236)	BB (3290)	BB (2800)
rs17114329	57241533	AA (2385)	AA (3236)	AA (3236)	AA (3236)	AA (3290)	AA (2800)
rs17114332	57243285	AA (2385)	AA (3236)	AA (3236)	AA (3236)	AA (3290)	AA (2800)
rs17372114	57243454	BB (2385)	BB (3236)	BB (3236)	BB (3236)	BB (3290)	BB (2800)
rs1738403	57246559	BB (2385)	BB (3236)	BB (3236)	BB (3236)	BB (3290)	BB (2800)
rs1774825	57249558	AA (2385)	AA (3236)	AA (3236)	AA (3236)	AA (3290)	AA (2800)
rs10889017	57259632	BB (2385)	BB (3236)	BB (3236)	BB (3236)	BB (3290)	BB (2800)
rs1891413	57259674	AA (2385)	AA (3236)	AA (3236)	AA (3236)	AA (3290)	AA (2800)
rs1361727	57266676	BB (2385)	BB (3236)	BB (3236)	BB (3236)	BB (3290)	BB (2800)
rs1361729	57267749	BB (2385)	BB (3236)	BB (3236)	BB (3236)	BB (3290)	BB (2800)
rs1361730	57267946	AA (2385)	AA (3236)	AA (3236)	AA (3236)	AA (3290)	AA (2800)
rs1342372	57268278	AA (2385)	AA (3236)	AA (3236)	AA (3236)	AA (3290)	AA (2800)
rs41417144	57268437	AA (2385)	AA (3236)	AA (3236)	AA (3236)	AA (3290)	AA (2800)
rs17114382	57270093	BB (2385)	BB (3236)	BB (3236)	BB (3236)	BB (3290)	BB (2800)

rs17114386	57272376		AA (2385)	AA (3236)	AA (3236)	AA (3236)	AA (3290)	AA (2800)
rs10489622	57275653		AA (2385)	AA (3236)	AA (3236)	AA (3236)	AA (3290)	AA (2800)
rs17114400	57283769		AA (2385)	AA (3236)	AA (3236)	AA (3236)	AA (3290)	AA (2800)
rs17114404	57283889		BB (2385)	BB (3236)	BB (3236)	BB (3236)	BB (3290)	BB (2800)
rs17114421	57291016		BB (2385)	BB (3236)	BB (3236)	BB (3236)	BB (3290)	BB (2800)
rs10789042	57291133		BB (2385)	BB (3236)	BB (3236)	BB (3236)	BB (3290)	BB (2800)
rs10889020	57291371		BB (2385)	BB (3236)	BB (3236)	BB (3236)	BB (3290)	BB (2800)
rs12048846	57291520		AA (2385)	AA (3236)	AA (3236)	AA (3236)	AA (3290)	AA (2800)
rs947636	57291548		BB (2385)	BB (3236)	BB (3236)	BB (3236)	BB (3290)	BB (2800)
rs12032625	57301685		BB (2385)	BB (3236)	BB (3236)	BB (3236)	BB (3290)	BB (2800)
rs737189	57309137		AA (2385)	AA (3236)	AA (3236)	AA (3236)	AA (3290)	AA (2800)
rs668551	57318918		AA (2385)	AA (3236)	AA (3236)	AA (3236)	AA (3290)	AA (2800)
rs664142	57325855		AA (2385)	AA (3236)	AA (3236)	AA (3236)	AA (3290)	AA (2800)
rs6697767	57326073		AA (2385)	AA (3236)	AA (3236)	AA (3236)	AA (3290)	AA (2800)
rs17114468	57326235		BB (2385)	BB (3236)	BB (3236)	BB (3236)	BB (3290)	BB (2800)
rs622299	57328122		AA (2385)	AA (3236)	AA (3236)	AA (3236)	AA (3290)	AA (2800)
rs594105	57343256		BB (2385)	BB (3236)	BB (3236)	BB (3236)	BB (3290)	BB (2800)
rs6588657	57348436		AA (2385)	AA (3236)	AA (3236)	AA (3236)	AA (3290)	AA (2800)
rs6694643	57349407		AA (2385)	AA (3236)	AA (3236)	AA (3236)	AA (3290)	AA (2800)
rs10489624	57349614		AA (2385)	AA (3236)	AA (3236)	AA (3236)	AA (3290)	AA (2800)
rs6683663	57356476		BB (2385)	BB (3236)	BB (3236)	BB (3236)	BB (3290)	BB (2800)
rs17114516	57358793		AA (2385)	AA (3236)	AA (3236)	AA (3236)	AA (3290)	AA (2800)
rs12116668	57371133		BB (2385)	BB (3236)	BB (3236)	BB (3236)	BB (3290)	BB (2800)
rs706479	57372463		AA (2385)	AA (3236)	AA (3236)	AA (3236)	AA (3290)	AA (2800)
rs619545	57372591		AA (2385)	AA (3236)	AA (3236)	AA (3236)	AA (3290)	AA (2800)
rs1741988	57375871		AA (2385)	AA (3236)	AA (3236)	AA (3236)	AA (3290)	AA (2800)
rs17114572	57376050		BB (2385)	BB (3236)	BB (3236)	BB (3236)	BB (3290)	BB (2800)
rs638919	57377172		AA (2385)	AA (3236)	AA (3236)	AA (3236)	AA (3290)	AA (2800)
rs9437011	57377497		BB (2385)	BB (3236)	BB (3236)	BB (3236)	BB (3290)	BB (2800)
rs1774897	57377526		AA (2385)	AA (3236)	AA (3236)	AA (3236)	AA (3290)	AA (2800)
rs687628	57377579		BB (2385)	BB (3236)	BB (3236)	BB (3236)	BB (3290)	BB (2800)
rs1774895	57380032		BB (2385)	BB (3236)	BB (3236)	BB (3236)	BB (3290)	BB (2800)
rs1612309	57380964		BB (2385)	BB (3236)	BB (3236)	BB (3236)	BB (3290)	BB (2800)
rs17114605	57380985		AA (2385)	AA (3236)	AA (3236)	AA (3236)	AA (3290)	AA (2800)
rs17373091	57391310		AA (2385)	AA (3236)	AA (3236)	AA (3236)	AA (3290)	AA (2800)
rs17301090	57391429		BB (2385)	BB (3236)	BB (3236)	BB (3236)	BB (3290)	BB (2800)
rs591730	57396014		AA (2385)	AA (3236)	AA (3236)	AA (3236)	AA (3290)	AA (2800)
rs638423	57396542		AA (2385)	AA (3236)	AA (3236)	AA (3236)	AA (3290)	AA (2800)
rs684844	57397039		AA (2385)	AA (3236)	AA (3236)	AA (3236)	AA (3290)	AA (2800)
rs17301153	57400984		AA (2385)	AA (3236)	AA (3236)	AA (3236)	AA (3290)	AA (2800)
rs649069	57419226		AA (2385)	AA (3236)	AA (3236)	AA (3236)	AA (3290)	AA (2800)
rs41452950	57420627		BB (2385)	BB (3236)	BB (3236)	BB (3236)	BB (3290)	BB (2800)
rs599857	57428589		AA (2385)	AA (3236)	AA (3236)	AA (3236)	AA (3290)	AA (2800)
rs2236217	57430805		AA (2385)	AA (3236)	AA (3236)	AA (3236)	AA (3290)	AA (2800)
rs17114693	57436665		BB (2385)	BB (3236)	BB (3236)	BB (3236)	BB (3290)	BB (2800)
rs17449278	57437251		AA (2385)	AA (3236)	AA (3236)	AA (3236)	AA (3290)	AA (2800)
rs1774918	57437735		BB (2385)	BB (3236)	BB (3236)	BB (3236)	BB (3290)	BB (2800)
rs1754509	57438416		BB (2385)	BB (3236)	BB (3236)	BB (3236)	BB (3290)	BB (2800)
rs1411008	57441625		BB (2385)	BB (3236)	BB (3236)	BB (3236)	BB (3290)	BB (2800)
rs1774915	57445502		AA (2385)	AA (3236)	AA (3236)	AA (3236)	AA (3290)	AA (2800)

rs12124958	57446294	AA (2385)	AA (3236)	AA (3236)	AA (3236)	AA (3290)	AA (2800)
rs12139688	57446318	AA (2385)	AA (3236)	AA (3236)	AA (3236)	AA (3290)	AA (2800)
rs1774909	57447740	BB (2385)	BB (3236)	BB (3236)	BB (3236)	BB (3290)	BB (2800)
rs1774908	57447820	BB (2385)	BB (3236)	BB (3236)	BB (3236)	BB (3290)	BB (2800)
rs1774905	57450300	AA (2385)	AA (3236)	AA (3236)	AA (3236)	AA (3290)	AA (2800)
rs11206947	57450441	AA (2385)	AA (3236)	AA (3236)	AA (3236)	AA (3290)	AA (2800)
rs11206948	57450486	AA (2385)	AA (3236)	AA (3236)	AA (3236)	AA (3290)	AA (2800)
rs6701615	57466885	AA (2385)	AA (3236)	AA (3236)	AA (3236)	AA (3290)	AA (2800)
rs12035887	57468791	BB (2385)	BB (3236)	BB (3236)	BB (3236)	BB (3290)	BB (2800)
rs17416642	57471621	BB (2385)	BB (3236)	BB (3236)	BB (3236)	BB (3290)	BB (2800)
rs1854508	57478856	BB (2385)	BB (3236)	BB (3236)	BB (3236)	BB (3290)	BB (2800)
rs4912423	57511521	BB (2385)	BB (3236)	BB (3236)	BB (3236)	BB (3290)	BB (2800)
rs12133813	57525481	AA (2385)	AA (3236)	AA (3236)	AA (3236)	AA (3290)	AA (2800)
rs17114917	57529790	AA (2385)	AA (3236)	AA (3236)	AA (3236)	AA (3290)	AA (2800)
rs7529536	57531021	BB (2385)	BB (3236)	BB (3236)	BB (3236)	BB (3290)	BB (2800)
rs3738556	57535198	BB (2385)	BB (3236)	BB (3236)	BB (3236)	BB (3290)	BB (2800)
rs6657073	57537652	AA (2385)	AA (3236)	AA (3236)	AA (3236)	AA (3290)	AA (2800)
rs12731670	57540169	BB (2385)	BB (3236)	BB (3236)	BB (3236)	BB (3290)	BB (2800)
rs17114957	57547097	AA (2385)	AA (3236)	AA (3236)	AA (3236)	AA (3290)	AA (2800)
rs3768184	57547703	BB (2385)	BB (3236)	BB (3236)	BB (3236)	BB (3290)	BB (2800)
rs578544	57549156	BB (2385)	BB (3236)	BB (3236)	BB (3236)	BB (3290)	BB (2800)
rs514412	57551569	AA (2385)	AA (3236)	AA (3236)	AA (3236)	AA (3290)	AA (2800)
rs485090	57552423	AA (2385)	AA (3236)	AA (3236)	AA (3236)	AA (3290)	AA (2800)
rs17114981	57558671	BB (2385)	BB (3236)	BB (3236)	BB (3236)	BB (3290)	BB (2800)
rs489049	57575069	BB (2385)	BB (3236)	BB (3236)	BB (3236)	BB (3290)	BB (2800)
rs564575	57576551	BB (2385)	BB (3236)	BB (3236)	BB (3236)	BB (3290)	BB (2800)
rs522532	57580829	AA (2385)	AA (3236)	AA (3236)	AA (3236)	AA (3290)	AA (2800)
rs17115064	57581797	BB (2385)	BB (3236)	BB (3236)	BB (3236)	BB (3290)	BB (2800)
rs534080	57584721	AA (2385)	AA (3236)	AA (3236)	AA (3236)	AA (3290)	AA (2800)
rs553453	57595290	AA (2385)	AA (3236)	AA (3236)	AA (3236)	AA (3290)	AA (2800)
rs556204	57595583	AA (2385)	AA (3236)	AA (3236)	AA (3236)	AA (3290)	AA (2800)
rs12090414	57600403	AA (2385)	AA (3236)	AA (3236)	AA (3236)	AA (3290)	AA (2800)
rs535415	57605889	AA (2385)	AA (3236)	AA (3236)	AA (3236)	AA (3290)	AA (2800)
rs546583	57614330	AA (2385)	AA (3236)	AA (3236)	AA (3236)	AA (3290)	AA (2800)
rs17115220	57615514	AA (2385)	AA (3236)	AA (3236)	AA (3236)	AA (3290)	AA (2800)
rs12059674	57630278	BB (2385)	BB (3236)	BB (3236)	BB (3236)	BB (3290)	BB (2800)
rs155293	57634943	BB (2385)	BB (3236)	BB (3236)	BB (3236)	BB (3290)	BB (2800)
rs6680219	57637298	BB (2385)	BB (3236)	BB (3236)	BB (3236)	BB (3290)	BB (2800)
rs7548633	57647124	BB (2385)	BB (3236)	BB (3236)	BB (3236)	BB (3290)	BB (2800)
rs17115265	57651129	BB (2385)	BB (3236)	BB (3236)	BB (3236)	BB (3290)	BB (2800)
rs17482980	57651809	BB (2385)	BB (3236)	BB (3236)	BB (3236)	BB (3290)	BB (2800)
rs197106	57658204	AA (2385)	AA (3236)	AA (3236)	AA (3236)	AA (3290)	AA (2800)
rs11206994	57661973	AA (2385)	AA (3236)	AA (3236)	AA (3236)	AA (3290)	AA (2800)
rs476466	57663923	BB (2385)	BB (3236)	BB (3236)	BB (3236)	BB (3290)	BB (2800)
rs1053	57671663	AA (2385)	AA (3236)	AA (3236)	AA (3236)	AA (3290)	AA (2800)
rs264036	57671853	AA (2385)	AA (3236)	AA (3236)	AA (3236)	AA (3290)	AA (2800)
rs197610	57672079	BB (2385)	BB (3236)	BB (3236)	BB (3236)	BB (3290)	BB (2800)
rs10489465	57672097	AA (2385)	AA (3236)	AA (3236)	AA (3236)	AA (3290)	AA (2800)
rs17115366	57672426	BB (2385)	BB (3236)	BB (3236)	BB (3236)	BB (3290)	BB (2800)
rs10489466	57677930	BB (2385)	BB (3236)	BB (3236)	BB (3236)	BB (3290)	BB (2800)

rs197605	57679193	BB (2385)	BB (3236)	BB (3236)	BB (3236)	BB (3290)	BB (2800)
rs1504584	57690336	BB (2385)	BB (3236)	BB (3236)	BB (3236)	BB (3290)	BB (2800)
rs1604885	57690668	AA (2385)	AA (3236)	AA (3236)	AA (3236)	AA (3290)	AA (2800)
rs12130571	57698001	BB (2385)	BB (3236)	BB (3236)	BB (3236)	BB (3290)	BB (2800)
rs706379	57698117	BB (2385)	BB (3236)	BB (3236)	BB (3236)	BB (3290)	BB (2800)
rs17115424	57703530	BB (2385)	BB (3236)	BB (3236)	BB (3236)	BB (3290)	BB (2800)
rs2764663	57703570	BB (2385)	BB (3236)	BB (3236)	BB (3236)	BB (3290)	BB (2800)
rs17115441	57707801	BB (2385)	BB (3236)	BB (3236)	BB (3236)	BB (3290)	BB (2800)
rs197626	57709358	BB (2385)	BB (3236)	BB (3236)	BB (3236)	BB (3290)	BB (2800)
rs12066220	57710254	BB (2385)	BB (3236)	BB (3236)	BB (3236)	BB (3290)	BB (2800)
rs614488	57712996	AA (2385)	AA (3236)	AA (3236)	AA (3236)	AA (3290)	AA (2800)
rs542873	57714529	AA (2385)	AA (3236)	AA (3236)	AA (3236)	AA (3290)	AA (2800)
rs12132898	57721731	BB (2385)	BB (3236)	BB (3236)	BB (3236)	BB (3290)	BB (2800)
rs197642	57722753	AA (2385)	AA (3236)	AA (3236)	AA (3236)	AA (3290)	AA (2800)
rs197637	57726159	BB (2385)	BB (3236)	BB (3236)	BB (3236)	BB (3290)	BB (2800)
rs197636	57726537	AA (2385)	AA (3236)	AA (3236)	AA (3236)	AA (3290)	AA (2800)
rs197635	57726749	BB (2385)	BB (3236)	BB (3236)	BB (3236)	BB (3290)	BB (2800)
rs12136930	57727450	AA (2385)	AA (3236)	AA (3236)	AA (3236)	AA (3290)	AA (2800)
rs12085771	57727539	AA (2385)	AA (3236)	AA (3236)	AA (3236)	AA (3290)	AA (2800)
rs17455073	57728262	BB (2385)	BB (3236)	BB (3236)	BB (3236)	BB (3290)	BB (2800)
rs1811700	57733378	AA (2385)	AA (3236)	AA (3236)	AA (3236)	AA (3290)	AA (2800)
rs41515244	57740686	AA (2385)	AA (3236)	AA (3236)	AA (3236)	AA (3290)	AA (2800)
rs2764664	57741163	AA (2385)	AA (3236)	AA (3236)	AA (3236)	AA (3290)	AA (2800)
rs1424473	57741329	BB (2385)	BB (3236)	BB (3236)	BB (3236)	BB (3290)	BB (2800)
rs694060	57743244	AA (2385)	AA (3236)	AA (3236)	AA (3236)	AA (3290)	AA (2800)
rs648607	57747403	AA (2385)	AA (3236)	AA (3236)	AA (3236)	AA (3290)	AA (2800)
rs12562394	57766176	BB (2385)	BB (3236)	BB (3236)	BB (3236)	BB (3290)	BB (2800)
rs622423	57766386	BB (2385)	BB (3236)	BB (3236)	BB (3236)	BB (3290)	BB (2800)
rs545582	57767050	BB (2385)	BB (3236)	BB (3236)	BB (3236)	BB (3290)	BB (2800)
rs17422457	57780247	AA (2385)	AA (3236)	AA (3236)	AA (3236)	AA (3290)	AA (2800)
rs267642	57780985	AA (2385)	AA (3236)	AA (3236)	AA (3236)	AA (3290)	AA (2800)
rs267644	57790529	BB (2385)	BB (3236)	BB (3236)	BB (3236)	BB (3290)	BB (2800)
rs6693181	57799785	BB (2385)	BB (3236)	BB (3236)	BB (3236)	BB (3290)	BB (2800)
rs6587767	57800285	AA (2385)	AA (3236)	AA (3236)	AA (3236)	AA (3290)	AA (2800)
rs17422855	57801345	BB (2385)	BB (3236)	BB (3236)	BB (3236)	BB (3290)	BB (2800)
rs6657926	57801950	BB (2385)	BB (3236)	BB (3236)	BB (3236)	BB (3290)	BB (2800)
rs17456828	57802255	BB (2385)	BB (3236)	BB (3236)	BB (3236)	BB (3290)	BB (2800)
rs12087888	57802759	AA (2385)	AA (3236)	AA (3236)	AA (3236)	AA (3290)	AA (2800)
rs11207010	57802886	BB (2385)	BB (3236)	BB (3236)	BB (3236)	BB (3290)	BB (2800)
rs1077424	57810304	AA (2385)	AA (3236)	AA (3236)	AA (3236)	AA (3290)	AA (2800)
rs1557223	57810618	AA (2385)	AA (3236)	AA (3236)	AA (3236)	AA (3290)	AA (2800)
rs6687842	57815932	AB (0)	AA (3236)	AA (3236)	AA (3236)	AA (3290)	AA (2800)
rs6687934	57816011	AB (0)	AA (3236)	AA (3236)	AA (3236)	AA (3290)	AA (2800)
rs11207016	57816224	AB (0)	AA (3236)	AA (3236)	AA (3236)	AA (3290)	AA (2800)
rs1424467	57816489	AB (0)	BB (3236)	BB (3236)	BB (3236)	BB (3290)	BB (2800)
rs41324751	57826473	AA (1)	AA (3236)	AA (3236)	AA (3236)	AA (3290)	AA (2800)
rs10889038	57826766	AB (0)	AA (3236)	AA (3236)	AA (3236)	AA (3290)	AA (2800)
rs10889039	57828545	AB (0)	AA (3236)	AA (3236)	AA (3236)	AA (3290)	AA (2800)
rs10493218	57847075	AA (7)	AA (3236)	AA (3236)	AA (3236)	AA (3290)	AA (2800)
rs17115713	57850735	BB (7)	BB (3236)	BB (3236)	BB (3236)	BB (3290)	BB (2800)

rs17424691	57851320	AA (7)	AA (3236)	AA (3236)	AA (3236)	AA (3290)	AA (2800)
rs166552	57853748	AA (7)	AA (3236)	AA (3236)	AA (3236)	AA (3290)	AA (2800)
rs3844040	57854451	AA (7)	AA (3236)	AA (3236)	AA (3236)	AA (3290)	AA (2800)
rs17115722	57855219	BB (7)	BB (3236)	BB (3236)	BB (3236)	BB (3290)	BB (2800)
rs507466	57855425	AA (7)	AA (3236)	AA (3236)	AA (3236)	AA (3290)	AA (2800)
rs532926	57855876	AB (0)	BB (3236)	BB (3236)	BB (3236)	BB (3290)	BB (2800)
rs566847	57857310	AB (0)	AA (3236)	AA (3236)	AA (3236)	AA (3290)	AA (2800)
rs567773	57857390	AB (0)	AA (3236)	AA (3236)	AA (3236)	AA (3290)	AA (2800)
rs10493220	57858457	AA (1)	AA (3236)	AA (3236)	AA (3236)	AA (3290)	AA (2800)
rs540431	57859521	AB (0)	BB (3236)	BB (3236)	BB (3236)	-3290	BB (2800)
rs11207026	57860215	AB (0)	AA (3236)	AA (3236)	AA (3236)	AA (3290)	AA (2800)
rs17115739	57863387	BB (3)	BB (3236)	BB (3236)	BB (3236)	BB (3290)	BB (2800)
rs3850553	57865919	AA (3)	AA (3236)	AA (3236)	AA (3236)	AA (3290)	AA (2800)
rs35404211	57866049	AA (3)	AA (3236)	AA (3236)	AA (3236)	AA (3290)	AA (2800)
rs2405994	57871016	AB (0)	BB (3236)	BB (3236)	BB (3236)	BB (3290)	BB (2800)
rs17115761	57872860	AA (1)	AA (3236)	AA (3236)	AA (3236)	AA (3290)	AA (2800)
rs11585751	57872987	AB (0)	AA (3236)	AA (3236)	AA (3236)	AA (3290)	AA (2800)
rs269042	57874048	AB (0)	AA (3236)	AA (3236)	AA (3236)	AA (3290)	AA (2800)
rs17115767	57875690	BB (19)	BB (3236)	BB (3236)	BB (3236)	BB (3290)	BB (2800)
rs12045017	57914548	AA (19)	AA (3236)	AA (3236)	AA (3236)	AA (3290)	AA (2800)
rs17460074	57916599	AA (19)	AA (3236)	AA (3236)	AA (3236)	AA (3290)	AA (2800)
rs17426429	57920416	BB (19)	BB (3236)	BB (3236)	BB (3236)	BB (3290)	BB (2800)
rs17115807	57921505	AA (19)	AA (3236)	AA (3236)	AA (3236)	AA (3290)	AA (2800)
rs12407896	57933955	BB (19)	BB (3236)	BB (3236)	BB (3236)	BB (3290)	BB (2800)
rs10493223	57934034	AA (19)	AA (3236)	AA (3236)	AA (3236)	AA (3290)	AA (2800)
rs17115844	57934206	BB (19)	BB (3236)	BB (3236)	BB (3236)	BB (3290)	BB (2800)
rs17115852	57939359	BB (19)	BB (3236)	BB (3236)	BB (3236)	BB (3290)	BB (2800)
rs2805873	57940990	AA (19)	AA (3236)	AA (3236)	AA (3236)	AA (3290)	AA (2800)
rs2805878	57951580	BB (19)	BB (3236)	BB (3236)	BB (3236)	BB (3290)	BB (2800)
rs2691462	57954416	AA (19)	AA (3236)	AA (3236)	AA (3236)	AA (3290)	AA (2800)
rs17115864	57954677	AA (19)	AA (3236)	AA (3236)	AA (3236)	AA (3290)	AA (2800)
rs17115882	57958048	AA (19)	AA (3236)	AA (3236)	AA (3236)	AA (3290)	AA (2800)
rs2691467	57961554	AA (19)	AA (3236)	AA (3236)	AA (3236)	AA (3290)	AA (2800)
rs1341320	57966528	AA (19)	AA (3236)	AA (3236)	AA (3236)	AA (3290)	AA (2800)
rs1547793	57966812	AA (19)	AA (3236)	AA (3236)	AA (3236)	AA (3290)	AA (2800)
rs2691429	57969583	AA (19)	AA (3236)	AA (3236)	AA (3236)	AA (3290)	AA (2800)
rs2793622	57971846	AA (19)	AA (3236)	AA (3236)	AA (3236)	AA (3290)	AA (2800)
rs10889048	58003035	AB (0)	AA (3236)	AA (3236)	AA (3236)	AA (3290)	AA (2800)
rs1524713	58003131	AB (0)	BB (3236)	BB (3236)	BB (3236)	BB (3290)	BB (2800)
rs1524715	58003409	AB (0)	BB (3236)	BB (3236)	BB (3236)	BB (3290)	BB (2800)
rs11207058	58003804	AB (0)	AA (3236)	AA (3236)	AA (3236)	AA (3290)	AA (2800)
rs1524716	58005178	AB (0)	AA (3236)	AA (3236)	AA (3236)	AA (3290)	AA (2800)
rs2793624	58006318	AA (3)	AA (3236)	AA (3236)	AA (3236)	AA (3290)	AA (2800)
rs2691445	58006649	AA (3)	AA (3236)	AA (3236)	AA (3236)	AA (3290)	AA (2800)
rs2805850	58006699	AA (3)	AA (3236)	AA (3236)	AA (3236)	AA (3290)	AA (2800)
rs2691446	58009249	AB (0)	BB (3236)	BB (3236)	BB (3236)	BB (3290)	BB (2800)
rs1534048	58012616	AA (1)	AA (3236)	AA (3236)	AA (3236)	AA (3290)	AA (2800)
rs1880443	58015312	AB (0)	AA (3236)	AA (3236)	AA (3236)	AA (3290)	AA (2800)
rs7530027	58037504	AA (11)	AA (3236)	AA (3236)	AA (3236)	AA (3290)	AA (2800)
rs1404388	58043416	AA (11)	AA (3236)	AA (3236)	AA (3236)	AA (3290)	AA (2800)

rs1341321	58044168	AA (11)	AA (3236)	AA (3236)	AA (3236)	AA (3290)	AA (2800)
rs10493230	58050139	BB (11)	BB (3236)	BB (3236)	BB (3236)	BB (3290)	BB (2800)
rs17116089	58051878	AA (11)	AA (3236)	AA (3236)	AA (3236)	AA (3290)	AA (2800)
rs7555213	58062496	BB (11)	BB (3236)	BB (3236)	BB (3236)	BB (3290)	BB (2800)
rs7555442	58062765	BB (11)	BB (3236)	BB (3236)	BB (3236)	BB (3290)	BB (2800)
rs1572945	58069523	AA (11)	AA (3236)	AA (3236)	AA (3236)	AA (3290)	AA (2800)
rs1935046	58075285	AA (11)	AA (3236)	AA (3236)	AA (3236)	AA (3290)	AA (2800)
rs1341322	58079143	AA (11)	AA (3236)	AA (3236)	AA (3236)	AA (3290)	AA (2800)
rs1524721	58079584	AA (11)	AA (3236)	AA (3236)	AA (3236)	AA (3290)	AA (2800)
rs10789050	58084162	AB (0)	AA (3236)	AA (3236)	AA (3236)	AA (3290)	AA (2800)
rs12070504	58084901	AA (2)	AA (3236)	AA (3236)	AA (3236)	AA (3290)	AA (2800)
rs17116134	58093163	BB (2)	BB (3236)	BB (3236)	BB (3236)	BB (3290)	BB (2800)
rs11207080	58100195	AB (0)	AA (3236)	AA (3236)	AA (3236)	AA (3290)	AA (2800)
rs1524707	58102540	BB (1)	BB (3236)	BB (3236)	BB (3236)	BB (3290)	BB (2800)
rs11800684	58103010	AB (0)	AA (3236)	AA (3236)	AA (3236)	AA (3290)	AA (2800)
rs17116148	58105811	BB (1)	BB (3236)	BB (3236)	BB (3236)	BB (3290)	BB (2800)
rs4484949	58111045	AB (0)	BB (3236)	BB (3236)	BB (3236)	BB (3290)	BB (2800)
rs6587787	58125050	AB (0)	AA (3236)	AA (3236)	AA (3236)	AA (3290)	AA (2800)
rs4244011	58129636	BB (1)	BB (3236)	BB (3236)	BB (3236)	BB (3290)	BB (2800)
rs3131776	58162616	AB (0)	AA (3236)	AA (3236)	AA (3236)	AA (3290)	AA (2800)
rs3118021	58173935	AA (35)	AA (3236)	AA (3236)	AA (3236)	AA (3290)	AA (2800)
rs1323815	58177003	BB (35)	BB (3236)	BB (3236)	BB (3236)	BB (3290)	BB (2800)
rs3131715	58178278	AA (35)	AA (3236)	AA (3236)	AA (3236)	AA (3290)	AA (2800)
rs716417	58178715	BB (35)	BB (3236)	BB (3236)	BB (3236)	BB (3290)	BB (2800)
rs3131725	58191387	BB (35)	BB (3236)	BB (3236)	BB (3236)	BB (3290)	BB (2800)
rs17431555	58208277	BB (35)	BB (3236)	BB (3236)	BB (3236)	BB (3290)	BB (2800)
rs3131734	58209220	AA (35)	AA (3236)	AA (3236)	AA (3236)	AA (3290)	AA (2800)
rs3118046	58210433	AA (35)	AA (3236)	AA (3236)	AA (3236)	AA (3290)	AA (2800)
rs2406279	58212131	AA (35)	AA (3236)	AA (3236)	AA (3236)	AA (3290)	AA (2800)
rs7534106	58215034	AA (35)	AA (3236)	AA (3236)	AA (3236)	AA (3290)	AA (2800)
rs17472030	58221074	AA (35)	AA (3236)	AA (3236)	AA (3236)	AA (3290)	AA (2800)
rs11207100	58224212	BB (35)	BB (3236)	BB (3236)	BB (3236)	BB (3290)	BB (2800)
rs35438587	58224332	BB (35)	BB (3236)	BB (3236)	BB (3236)	BB (3290)	BB (2800)
rs3118053	58226522	BB (35)	BB (3236)	BB (3236)	BB (3236)	BB (3290)	BB (2800)
rs1323822	58226592	BB (35)	BB (3236)	BB (3236)	BB (3236)	BB (3290)	BB (2800)
rs1323823	58226667	BB (35)	BB (3236)	BB (3236)	BB (3236)	BB (3290)	BB (2800)
rs1323824	58226786	AA (35)	AA (3236)	AA (3236)	AA (3236)	AA (3290)	AA (2800)
rs3131741	58227195	AA (35)	AA (3236)	AA (3236)	AA (3236)	AA (3290)	AA (2800)
rs11207103	58232061	AA (35)	AA (3236)	AA (3236)	AA (3236)	AA (3290)	AA (2800)
rs2406282	58232329	BB (35)	BB (3236)	BB (3236)	BB (3236)	BB (3290)	BB (2800)
rs1323827	58232720	AA (35)	AA (3236)	AA (3236)	AA (3236)	AA (3290)	AA (2800)
rs927612	58234759	BB (35)	BB (3236)	BB (3236)	BB (3236)	BB (3290)	BB (2800)
rs3118059	58235497	BB (35)	BB (3236)	BB (3236)	BB (3236)	BB (3290)	BB (2800)
rs12143653	58238545	BB (35)	BB (3236)	BB (3236)	BB (3236)	BB (3290)	BB (2800)
rs3131745	58238692	BB (35)	BB (3236)	BB (3236)	BB (3236)	BB (3290)	BB (2800)
rs2147077	58244952	AA (35)	AA (3236)	AA (3236)	AA (3236)	AA (3290)	AA (2800)
rs1886143	58258134	AA (35)	AA (3236)	AA (3236)	AA (3236)	AA (3290)	AA (2800)
rs12068895	58259108	BB (35)	BB (3236)	BB (3236)	BB (3236)	BB (3290)	BB (2800)
rs1570595	58259463	AA (35)	AA (3236)	AA (3236)	AA (3236)	AA (3290)	AA (2800)
rs11207108	58259705	BB (35)	BB (3236)	BB (3236)	BB (3236)	BB (3290)	BB (2800)

rs7544285	58263118	AA (35)	AA (3236)	AA (3236)	AA (3236)	AA (3290)	AA (2800)
rs7544843	58263767	BB (35)	BB (3236)	BB (3236)	BB (3236)	BB (3290)	BB (2800)
rs11207110	58271009	BB (35)	BB (3236)	BB (3236)	BB (3236)	BB (3290)	BB (2800)
rs11207111	58271285	AA (35)	AA (3236)	AA (3236)	AA (3236)	AA (3290)	AA (2800)
rs852768	58287622	AA (35)	AA (3236)	AA (3236)	AA (3236)	AA (3290)	AA (2800)
rs852787	58302102	AB (0)	BB (3236)	BB (3236)	BB (3236)	BB (3290)	BB (2800)
